# Qiber3D—an open-source software package for the quantitative analysis of networks from 3D image stacks

**DOI:** 10.1093/gigascience/giab091

**Published:** 2022-02-04

**Authors:** Anna Jaeschke, Hagen Eckert, Laura J Bray

**Affiliations:** Centre for Biomedical Technologies, Queensland University of Technology, 60 Musk Ave, Kelvin Grove, QLD 4059, Australia; School of Mechanical, Medical and Process Engineering, Science and Engineering Faculty, Queensland University of Technology, 2 George St, Brisbane City QLD 4000, Australia; Mechanobiology Institute, National University of Singapore, 5A Engineering Drive 1, Singapore 117411, Singapore; Department of Mechanical Engineering and Materials Science, Duke University, 144 Hudson Hall, Durham, NC 27708, USA; Centre for Biomedical Technologies, Queensland University of Technology, 60 Musk Ave, Kelvin Grove, QLD 4059, Australia; School of Mechanical, Medical and Process Engineering, Science and Engineering Faculty, Queensland University of Technology, 2 George St, Brisbane City QLD 4000, Australia; ARC Training Centre for Cell and Tissue Engineering Technologies, Queensland University of Technology, 60 Musk Ave, Kelvin Grove QLD 4059, Australia

**Keywords:** morphometric quantification, confocal imaging, image processing, vascular networks, fibrous networks, neurons

## Abstract

**Background:**

Optical slice microscopy is commonly used to observe cellular morphology in 3D tissue culture, e.g., the formation of cell-derived networks. The morphometric quantification of these networks is essential to study the cellular phenotype. Commonly, the quantitative measurements are performed on 2D projections of the image stack, resulting in the loss of information in the third dimension. Currently available 3D image analysis tools rely on manual interactions with the software and are therefore not feasible for large datasets.

**Findings:**

Here we present Qiber3D, an open-source image processing toolkit. The software package includes the essential image analysis procedures required for image processing, from the raw image to the quantified data. Optional pre-processing steps can be switched on/off depending on the input data to allow for analyzing networks from a variety of sources. Two reconstruction algorithms are offered to meet the requirements for a wide range of network types. Furthermore, Qiber3D’s rendering capabilities enable the user to inspect each step of the image analysis process interactively to ensure the creation of an optimal workflow for each application.

**Conclusions:**

Qiber3D is implemented as a Python package, and its source code is freely available at https://github.com/theia-dev/Qiber3D. The toolkit was designed using a building block principle to enable the analysis of a variety of structures, such as vascular networks, neuronal structures, or scaffolds from numerous input formats. While Qiber3D can be used interactively in the Python console, it is aimed at unsupervised automation to process large image datasets efficiently.

## Background

The process of angiogenesis, the development of new blood vessels from the existing vasculature, is the center of numerous research questions. Studying the processes involved in vessel formation, maturation, and remodeling is essential for a better understanding of normal development and angiogenesis-related disease stages [1,2]. *In vitro* angiogenesis models aim towards replicating the formation of vascular-like networks in the laboratory [2]. Optical slice microscopy is commonly used to follow vessel formation in *in vitro* angiogenesis models [3]. Thereby, multiple images are acquired across different positions in the *z*-plane throughout the specimen, capturing the cell morphology in 3D  [3]. The vascular phenotype can be assessed by qualitative observation or by morphometric quantification of fiber length, number of fibers, cross-sectional area, or volume, as well as branching [2]. The quantitative characterization of the morphological phenotype is an essential tool to study cellular responses. Currently, most morphometric measurement approaches rely on 2D projections, often maximum-intensity projections, of the 3D images. However, 2D quantification of 3D structures limits the accuracy of data obtained and results in the loss of relevant information in the third dimension [4]. Consequently, there is a need for quantification tools of 3D image files that can be adapted to various areas of research studying networks composed of elongated or fiber-like structures.

Computational approaches exist to visualize and investigate cell morphology in 2D and 3D. Proprietary software, e.g., Amira™ (ThermoFisher Scientific) [5], Imaris (Oxford Instruments), or Metamorph^®^ (Molecular Devices), is capable of 3D, 4D, and 5D image processing and analysis. However, proprietary software packages are often black boxes tailored to machines sold by the same companies. While the documentation usually covers the fundamental methodology of a function, the actual implementation is not revealed. Regularly, these software packages are designed to be stand-alone all-in-one products, making their automated integration into analysis protocols cumbersome. Furthermore, the licensing expenses restrict accessibility to these software packages and therefore significantly limit the transferability and reproducibility of protocols using them. A multitude of open-source image processing software packages capable of 3D image visualization and processing have been developed in response [6–8]. Many of these tools are widely extensible by the use of plugins [6,9]. Thereby, software that was not specifically developed for processing image stacks, such as ImageJ/Fiji [9], can be used for 3D image analysis.

Available 3D quantification protocols often combine existing software packages, and usually require manual handling, at least for parts of the image analysis workflow [10–12]. Besides carrying the risk of user-based subjectivity, this also limits the throughput of samples for experiments with large image datasets. In some cases, switching between multiple existing software packages is necessary [12], making the image processing time- and resource-consuming and, therefore, again, not feasible for large datasets.

Automation, at least for parts of the image analysis workflow, can be achieved through external scripts or, in the case of ImageJ/Fiji [9], by using macros. While this is a feasible route for smaller datasets, the automation of image processing tasks using tools designed primarily for a graphical user interface (GUI) is limited. These limitations become especially obvious if one aims at using high-performance computing (HPC) clusters or cloud computing resources. While the use of these tools on shared computing resources is challenging, running them without a GUI (headless) and unsupervised for a prolonged time requires extensive effort. Overall, it is impractical to design an unsupervised automated workflow that can quantify 3D structures in bulk with the available graphical image analysis tools.

Here we present Qiber3D, an open-source software package for morphometric quantification of networks from 3D image stacks. Qiber3D combines the required tools for a complete analytical workflow, from the raw image to final measured values. The core method of Qiber3D for the 3D reconstruction of networks is based on thinning. While this approach covers many applications, e.g., vascular-like networks or scaffolds, we also offer the kimimaro implementation of the Tree-structure Extraction Algorithm for Accurate and Robust skeletons (TEASAR) [13, 14] as an alternative skeletonization method. With the implementation of two reconstruction modes, Qiber3D is usable for the quantification of a variety of networks from image stacks.

Qiber3D generates a graph representation of a network based on a variety of input formats. The option to inspect the network interactively at each step of the workflow assists in optimizing the image processing parameters. The extracted quantitative morphometric data can be exported in a multitude of options to provide broad compatibility with other software. The implementation as an open-source Python package creates a highly customizable program suitable for image analysis automation and tight integration into existing workflows. By design, Qiber3D is suitable for applying general batch distribution approaches to be used on HPC clusters, enabling high-throughput image analysis for large datasets.

## Findings

### Design principles

Qiber3D is designed to quantify a large number of network image stacks without manual user intervention. To achieve this goal, we realized the toolkit within the Python ecosystem. The access to the wide selection of open-source modules, such as SciPy [15] or scikit-image  [16], enabled us to build upon a well-maintained foundation. Because the Python language is widely used in the scientific community, Qiber3D can be easily included as a building block into new and existing image analysis workflows. Using a Jupyter [17] notebook as an easy platform to develop new workflows directly on a shared computing resource will help to familiarize the user with Qiber3D quickly and enable collaborative work. Moreover, with the growing interest in machine-learning algorithms for computer vision tasks, the straightforward integration with toolkits such as TensorFlow [18] and PyTorch [19] provides an additional advantage. An often-cited drawback of using Python is the speed limitation compared to compiled languages. Python code needs to be interpreted at runtime and is therefore not optimized for the hardware it is running on. Memory usage needs to be considered with large input datasets because the native Python datatypes can be inefficient. These limitations are mitigated by the fact that most scientific routines utilized in Qiber3D are written in C or Fortran and compiled for the CPU architecture.

**Figure 1:**
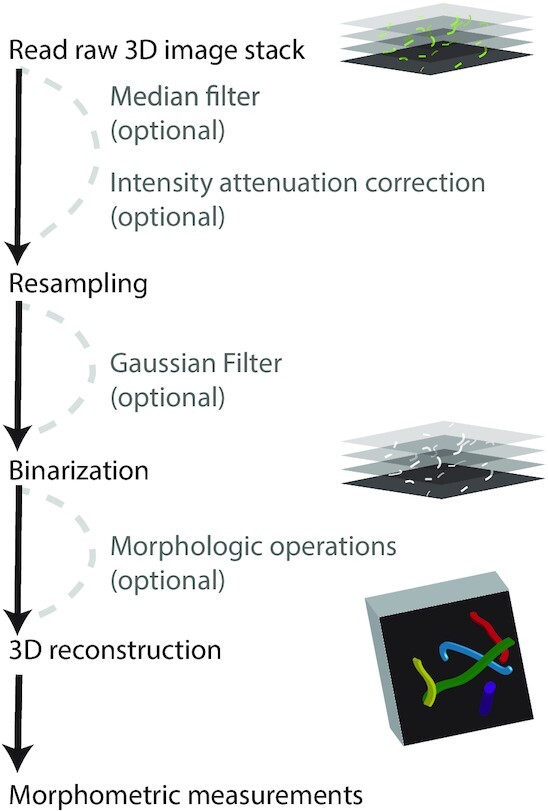
: Qiber3D's pipeline combines the required image processing steps for 3D morphometric quantification of networks. Optional tools are provided to cover a range of image content.

Qiber3D provides the tools for a complete analytical workflow, from the raw image input to the morphometric quantification. Aiming for high customizability, we provide a streamlined way to configure the various parameters used in Qiber3D. Optional steps can be included or excluded from the image processing pipeline (Fig. 1), allowing for Qiber3D to be applied on raw as well as pre-processed images from a variety of sources. We focused the software’s backbone on a selected set of tools that we could test extensively using the datasets available to us. Specific research questions and the nature of the input data may demand custom steps/extensions/algorithms. Because we cannot anticipate such requirements, we choose to design Qiber3D as compactly as possible. Eventually, every image processing protocol should be adapted for the input data and required measurements. While deconvolution and planar illumination correction are commonly used in image processing, they are not included in Qiber3D. During the design and testing of Qiber3D, we concluded that deconvolution was not beneficial for our example dataset and is probably not relevant for many users of this toolkit. Two measures can be influenced by the point-spread function (PSF) of the microscope: fiber radius and position. The point-spread primarily manifests by elongating the objects in the image stacks along the *z*-axis. Because the PSF is uniform over the image stack and the reconstruction functions find the center of the fibers, only a constant shift of the network along the *z*-axis is expected. Such a shift is without consequences for our purposes because we have no outer frame of reference. The radius along the fibers is measured by the shortest distance for each central voxel to the background. As the minimum is used, the *x*/*y*-plane with an often higher resolution becomes the dominant source for the radius definition. The typical PSF of the microscope has nearly no influence on the measured radii because the fibers are assumed to have a circular cross-section. All in all, we think that the effort necessary to generate a high-quality PSF and the time to compute the deconvolution is not required for most use cases. Uneven illumination correction in the *x*/*y*-plane was not suitable for our testing data. Slight changes in the illumination over the plane are already evened out by the binarization step. Moreover, there is a chance of introducing artifacts by correcting uneven illumination on a slice-by-slice basis. In cases where these steps are unavoidable, Qiber3D can be extended, utilizing the many implementations of image processing tasks readily available in Python. Overall, the open-source nature of the software avoids analytical black boxes and allows for researchers to tailor it to their data.

Qiber3D's test-driven design allows for well-structured collaborative development. Because the size of experimental image stacks restricts their use for integrated testing, we included a method to create *synthetic*network images. This method takes a reconstructed network as input and renders it as a layered 3D image that can subsequently be stored in the desired format. This allows for proper unit tests of the source code without the need to download large datasets. All in all, the open-source approach combined with the test-driven design enables the long-term evolution of the project through user contributions.

Qiber3D is developed as a command-line tool, enabling smooth integration into existing workflows, as well as automated, high-throughput image analysis. We are aware that building Qiber3D as a command-line tool results in a higher barrier to entry. Qiber3D and its documentation is designed to ease the transition into using command-line tools. Moreover, visualization using vedo [20] allows the user to interact with the image output at different stages during image processing.

### Implementation

#### Data IO

Because interoperability is an essential goal of the Qiber3D toolkit, a wide variety of import and export options is paramount. Confocal images are usually acquired using commercial imaging platforms, and the image files are saved in a proprietary file format containing the metadata. Qiber3D's support for multi-dimensional image formats is based on PIMS [21]. This choice allows the use of essential image formats like .tiff-stacks, as well as proprietary file formats from microscope vendors like Leica, Nikon, Olympus, and Zeiss, as input. Physical size information (the voxel size) and, for multi-channel images, the channel of interest for network reconstruction is provided upon image loading or set as configuration variable for automated workflows. For some file formats, Qiber3D is able to extract the required metadata directly from the input file. Besides loading 3D image stacks to create the “Network” object, it can be built from files describing the network. Qiber3D supports the MicroVisu3D format .mv3d, traditionally used for vascular networks, as well as the .swc and the .ntr format, popular for neuronal networks.

The internal representation of the Qiber3D network can be stored as a binary file (.qiber) that allows for fast loading of the reconstructed network into the software. Easy visualization in web applications, and the import into specialized rendering software like Blender, is achieved by saving the 3D representation as a collection of truncated cones in the .x3D file format. Moreover, Qiber3D supports several human-readable formats. The spatial data of the reconstructed network can be exported as .mv3d, .swc, and .csv files. When exporting to a .json or Microsoft Excel .xlsx file format using openpyx [22], the complete set of metadata and calculated properties is included. Furthermore, the network can be exported as a 3D .tiff image stack.

#### Image pre-processing

##### Median filter (optional)

The primary purpose of the 3D median filter, also known as the despeckle filter, is the removal of speckles and extrema [23]. The median of its surrounding voxels replaces the value of each voxel. By default, a 3-voxels-wide neighborhood is used. However, this size can be modified in the configuration depending on the noise present in the image.

##### Intensity attenuation correction (optional)

**Figure 2:**
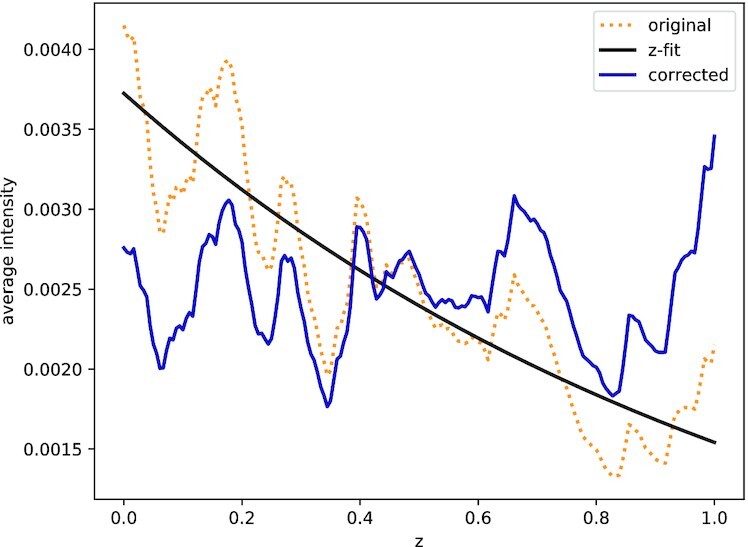
: Intensity attenuation correction in the example image of the microvascular network. Orange indicates original signal; blue, corrected signal; and black, intensity fit.

In 3D confocal images, light absorption can cause a decrease in signal intensity in slices located deeper into the sample. An exponential curve is fitted to the mean intensities *I*_A_ in each of the slices to their physical stack position *z* to correct for this intensity attenuation (Fig. 2).

(1) \begin{eqnarray*}
I_{\text{A}}=a\exp (bz) \end{eqnarray*}

The optimal parameters *a* and *b* for the intensity correction are determined using a non-linear least-squares fit.

##### Resampling to an isotropic voxel size

Commonly, the *x*/*y* resolution of image stacks differs from the resolution along the *z*-axis. As a cubic voxel size is beneficial to optimize the subsequent image processing steps, the *z*-axis or the *x*/*y*-plane of the image is resampled to a uniform resolution using a third-order spline interpolation [24].

##### Gaussian filter (optional)

The image stack is blurred with a Gaussian filter simultaneously in all three dimensions to minimize the effect of noise on the image segmentation by reducing sharp differences between neighboring pixels. Applying a Gaussian filter reduces the noise level and imaging artifacts significantly. Because the values now change smoothly from the outside to the inside of a structure, a border created by a cut-off will be more consistent and less rough.

#### Image segmentation

##### Binarization

The gray-scale image is reduced to a binary representation to locate the structures’ boundaries and label the segments. All voxels equal to or greater than a threshold are set to “True” and all others to “False.” A dynamic threshold calculation for each stack is performed, permitting an automated workflow. By default, Otsu thresholding, an unsupervised, nonparametric method that tries to maximize the separability of the resultant classes (exactly 2 in the binary image), by utilizing the zeroth- and first-order moments of the histogram [25], is applied. Other thresholding algorithms can be selected, depending on the image. Alternatively, the threshold can be set directly as a percentage value of the signal intensity.

##### Morphological operations (optional)

The obtained structures in the binarized image stack might not be perfectly solid, depending on the quality of the input data. A combination of dilation steps followed by an equal number of erosion steps fills small holes and compacts the segments’ surface. The number of steps is configurable. In this section, small islands caused by imaging artifacts can also be removed on the basis of a threshold set by the user.

#### Network reconstruction

##### Reconstruction by thinning (default)

The default network reconstruction approach is based on thinning, a morphological operation to remove selected foreground pixels from binary images. Initially, the image stack is distance transformed and every foreground (“True”) voxel in the stack is assigned the shortest Euclidean distance to a background (“False”) voxel. Subsequently, the Lee-Kashyap algorithm [26] is applied to extract the medial axis, and the binary image is reduced to its skeleton. The remaining foreground voxels, the skeleton, are modeled as a graph using NetworkX [27], defined by vertices that are connected by edges. Each foreground voxel represents a vertex, and connecting edges are formed between neighboring voxels. A radius is assigned to each vertex on the basis of the earlier distance transformation. To form “Segments” (see below for details), the graph is reduced to contain only vertices representing end and branch points.

**Figure 3:**
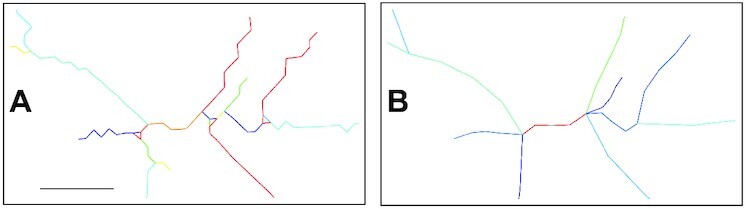
: Network optimization. After thinning (A), the network is optimized by replacing tiny segments with more extensive structures and smoothing out voxel artifacts (B). Scale bar: 12.3 μm (10  voxels).

Distinctive edges are often formed along with branch points, sharp bends, or on the network’s rim. Such edges occur between vertices that are direct neighbors, and the resulting path is particularly jagged (Fig. 3B). This resolution artifact results in an overestimation of the fiber length and volume and an inflated branch point count. To mitigate these drawbacks, edges shorter than 6 voxels are merged with larger neighbors or removed if isolated and each edge is interpolated using a cubic spline (Fig. 3B). New points are generated by default at a rate of ∼1 point every 10 voxels. All edges are fit to a spline with ≥5 points.

##### Reconstruction with TEASAR (alternative)

Initially, the TEASAR method aimed to generate organ centerlines from 3D imaging generated by MRI, or CT scans [13,14]. It has since been used in a variety of applications, from pore networks in clay rocks [28,29] to neuronal networks [30,31]. Qiber3D incorporates the kimimaro [32] implementation of the TEASAR algorithm that was developed to skeletonize neurons. For processing networks that resemble neuronal structures, i.e., branching of structures (dendrites) from a cell body (soma), the use of this method is recommended over the thinning-based reconstruction. The output of the skeletonization step is a connected graph, from which we extract the quantitative measurements of the network.

#### Morphometric measurement

**Figure 4:**
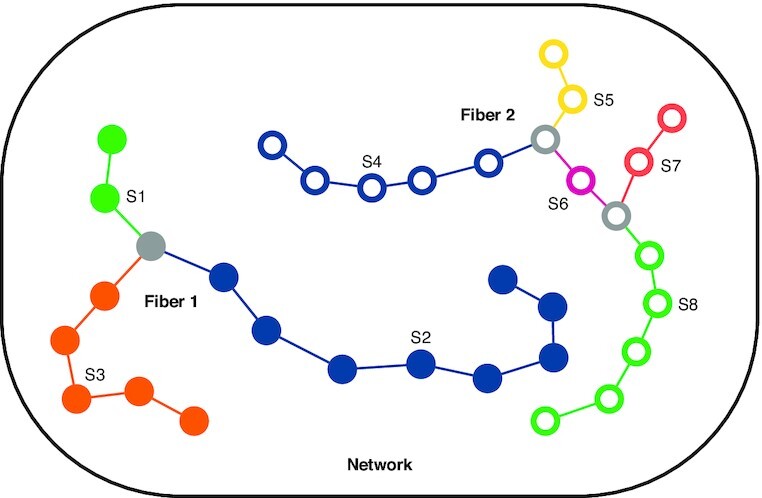
: Qiber3D’s hierarchical structure. Segments S1–S3 generate Fiber 1 (filled points), and segments S4–S8, Fiber 2 (hollow points), forming the Network. Branch points are colored in gray.

In Qiber3D the reconstructed network is represented in a hierarchical structure (Fig. 4). We use the terms “Network,” “Fiber,” and “Segments” to describe the components of the reconstruction. Note that these expressions are purely used conceptually to label Qiber3D’s output and that the terms might not refer to the actual structure. A Fiber might be a real fiber, an elongated cell, or another object depending on the application.

The largest entity is the Network, which represents the entirety of the structure. It is composed of a collection of Fibers formed by connected Segments, the smallest elements. A Segment is described by a collection of sorted points stored along the corresponding radius. The vertices between the points are interpreted as truncated cones. Segments end when they reach a branch point (gray points, Fig. 4). Therefore, Segments themselves are never branched. A branch point belongs to all Segments that it connects.

Each element, on the different hierarchical levels, is defined by a unique identifier and several quantitative properties, e.g., the volume or the mean radius. The mean radius can be misleading considering that the distance between the points forming an element can be non-uniform, resulting in a skewed measurement. Therefore, we included the notion of a length-weighted cylindrical radius and return the radius of a cylinder with the same volume and length as the element of interest. While modeling the volume as overlapping truncated cones is sufficient in most cases, an improved volume estimation can be obtained from the rasterized network. As the start and end points of a Fiber within a given 3D image stack are interchangeable, the directional data are analyzed on the basis of the assumption that all Fibers are pointing upwards (positive *z*-axis). Depending on the application, Fibers can be convoluted and the orientation of the Segments can be more meaningful in some cases. In both cases, the orientation of each element is described using the azimuth and altitude regarding a half-sphere.

For the Network additional measurements, such as the number of Fibers, Segments, and branch points, or the bounding box volume, are provided. The Network object also stores the relevant metadata of the input.

#### Visualization

Qiber3D uses vedo, a lightweight Python module, that is based on VTK [33] and numpy [34], to visualize the network in 3D. The embedded rendering capability allows the users to quickly inspect a network by rotating the camera view and zooming into regions of interest. A linked view of the different reconstruction steps and the resulting skeleton enables the user to examine them in relation to each other. The network’s color can be customized to represent different network properties, such as fiber length, volume, or mean radius. In addition to the interactive visualization, 3D views can be exported as static images or animations.

### Results

To provide a comprehensive overview of the features, Qiber3D was applied to the synthetic example image, as well as two experimental datasets, an *in vitro* microvascular network, and a neuron that was reconstructed elsewhere.

#### Synthetic example image

**Figure 5:**
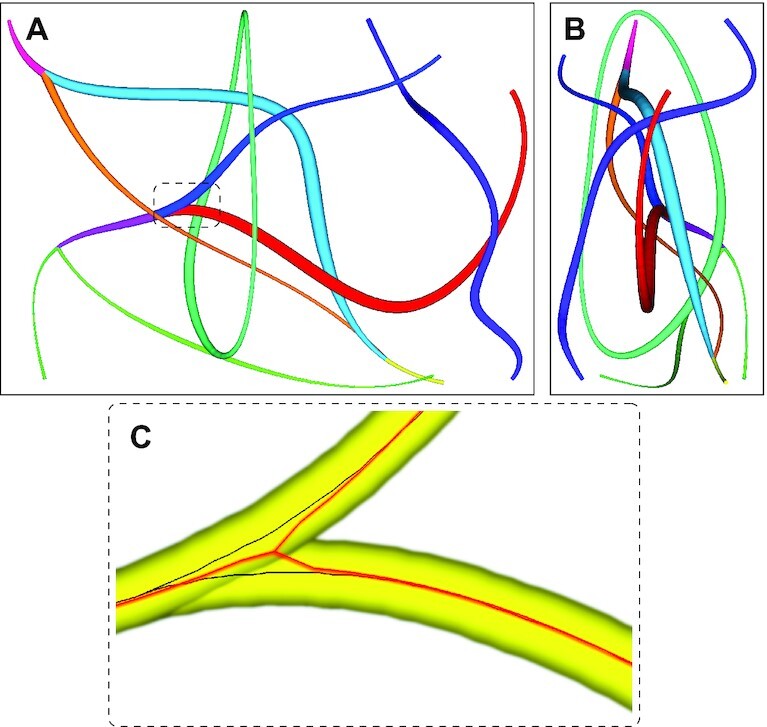
: Synthetic network example with (A) view on the *x*/*y*-plane and (B) view on the *z*/*y*-plane. (C) A branch point of the synthetic network with the original (black) and reconstructed (red) centerlines.

The output of the synthetic example image is presented in Fig. 5 and Supplementary Movie S1. The synthetic example network was visualized in 3D and the segments composing the fibers were observed (Fig. 5A). The measurements of the synthetic network reconstructed with Qiber3D were in agreement with the input data (Table 1). Interestingly, the branch points of the fibers were slightly displaced (Fig. 5C) without affecting the measured total volume of the synthetic network (Table 1). This discrepancy is due to the thickness of the fibers concealing the original merging points during reconstruction.

**Table 1.** Comparison of the synthetic network with the output of Qiber3D after reconstruction

Parameter	Synthetic network	Qiber3D output
No. of fibers	4	4
Total length (μm)	1,141.44	1,120.84
Total volume (μm^3^)	4,688.67	4,665.62
Mean radius (μm)	0.94	0.96
Cylinder radius (μm)	1.14	1.15

#### Microvascular network

**Figure 6:**
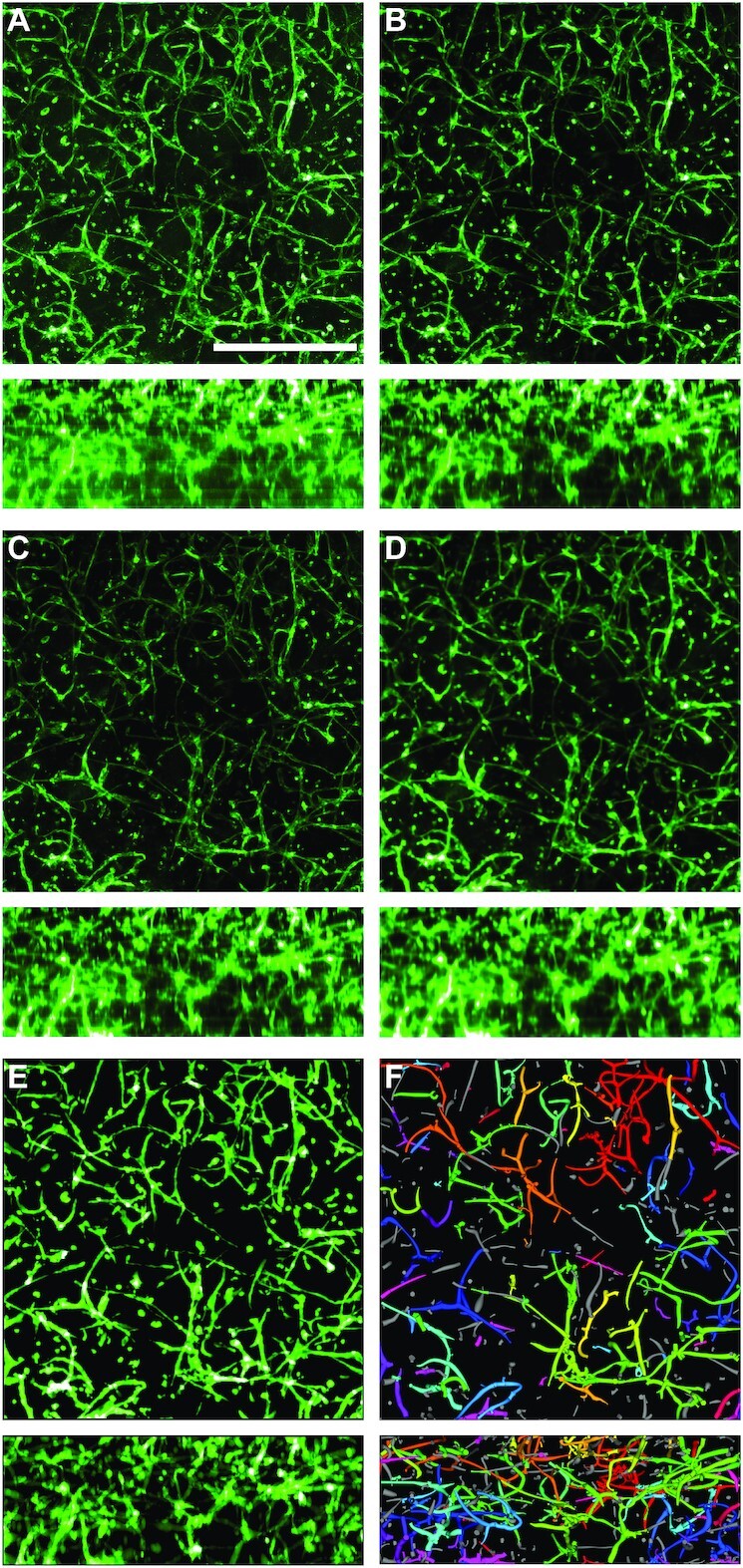
: Qiber3D's image processing workflow. An image of each step is shown as a mean intensity projection along the *z*-axis (upper panels) and along the *x*-axis (lower panels). (A) Raw image. Green indicates AlexaFluor 488-staining of CD31, a surface marker specific for endothelial cells. Scale bar: 500 μm. (B) Image after median filter. (C) Image corrected for intensity attenuation (*z*-drop correction). (D) Image after Gaussian blur and surface compacting. (E) Binarized image. (F) Reconstructed microvascular network.

Qiber3D was used to analyze a confocal image of a cellular network derived from microvascular cells grown *in vitro* (Fig. 6A).

The analysis was performed, including all optional procedures of the workflow (Fig. 6). The application of the median filter resulted in a clearer image with fewer extrema (Fig. 6B). Upon correction of the intensity attenuation, the signal distribution was found more even along the *z*-axis (compare Fig. 6B and C, lower panels). The quantitative observation was confirmed by the distribution of the mean signal intensity slice along the *z*-axis before (Fig. 2, orange line) and after (Fig. 2, blue line) the correction step. If the *z*-drop correction was switched off, the vessels in the lower part of the image were lost after reconstruction of the microvascular network (Supplementary Fig. S1B, D–F). Following the intensity attenuation correction, application of a Gaussian filter resulted in noise reduction and smoothing of the boundaries (Fig. 6D). After pre-processing the image using the optional filters, image segmentation was performed. Morphological operations, in the form of a combination of dilation and erosion (each with 5 iterations) and the removal of islands smaller than 100 μm^3^, were applied to the binary image (Fig. 6E). Omitting the morphological operations prior to reconstruction resulted in the presence of numerous small particles that were not connected to the microvascular network (“islands”) (Supplementary Fig. S1C–E). Finally, the skeleton of the microvascular network was successfully reconstructed from the 3D image stack (Fig. 6F, Supplementary Movie S2). Each step was visualized interactively while processing the input image and compared together afterwards (Supplementary Movie S3). Removing the optional filter steps for the image of the microvascular-like network led to artifacts in the reconstructed network (Supplementary Fig. S1B, E, and F).

**Figure 7:**
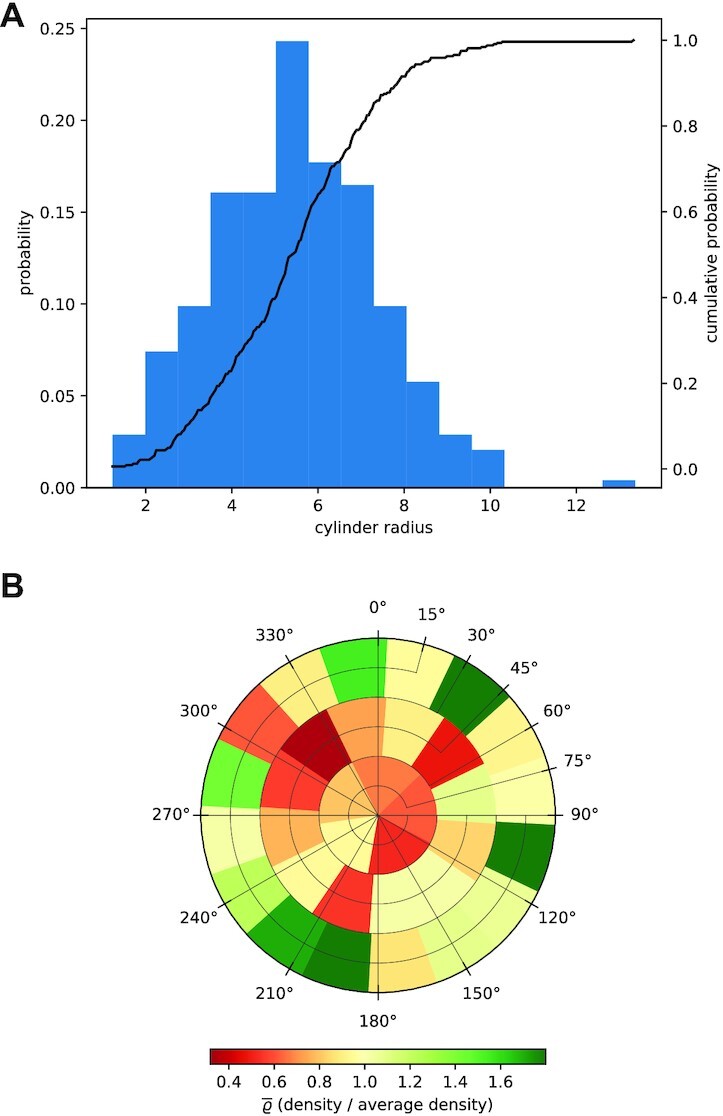
: Graphical output of quantitative data from the microvascular network in Qiber3D. (A) Distribution of the cylinder radius of the fibers within the network. (B) Orientation distribution of the fibers in 3D.

The distribution of network attributes can be visualized in Qiber3D in the form of a histogram. In Fig. 7A the distribution of the cylinder radius in the cellular network is presented as an example. The fiber radii followed a normal distribution between 1 and 10 μm, with a mean at 6.2 μm. To visualize the directional distribution in 3D, we introduced a spherical histogram. In Fig. 7B every bin represents a part of a half-sphere. The start point for every network fiber was considered to be at the center of the half-sphere. The segments of each fiber were averaged into a single vector that captures the fiber’s dominant direction. Because the surface areas of the different bins of a half-sphere are not perfectly equal, the number of intersecting vectors was divided by the bin’s surface area. Furthermore, the fiber density of each bin was scaled using the mean fiber density over the half-sphere to allow for streamlined comparisons between multiple networks. The color scale indicates the scaled fiber density. For the microvascular network, the majority of fibers are located parallel to the *x*/*y*-axis (Fig. 7B).

Processing a 1-GB nd2 file with Qiber3D on an Intel Core i7-6700 machine with 16 GB RAM running a Windows 10 (64-bit) operating system took ∼7.5 minutes. Manually analyzing a similar image takes ∼8.5 min, not considering the time to switch between various software packages [12]. While this is a slight decrease in processing time of 1 image, Qiber3D can be applied to numerous images without user interaction, making it suitable for analyzing large datasets. Because Qiber3D is designed to run on a single CPU, running multiple processes of Qiber3D in parallel will accelerate the mean image processing time for large datasets significantly. The use of built-in multiprocessing tools in Python enables straightforward implementation of parallel processing. For larger deployments on HPC clusters, task management using MPI for Python enables the analysis of vast image datasets. The implementation of Qiber3D as a Python package enables smooth integration with other Python libraries to build customized tools that meet the requirements of varying computational environments, e.g., different HPC centers.

#### Neuron morphology

**Figure 8:**
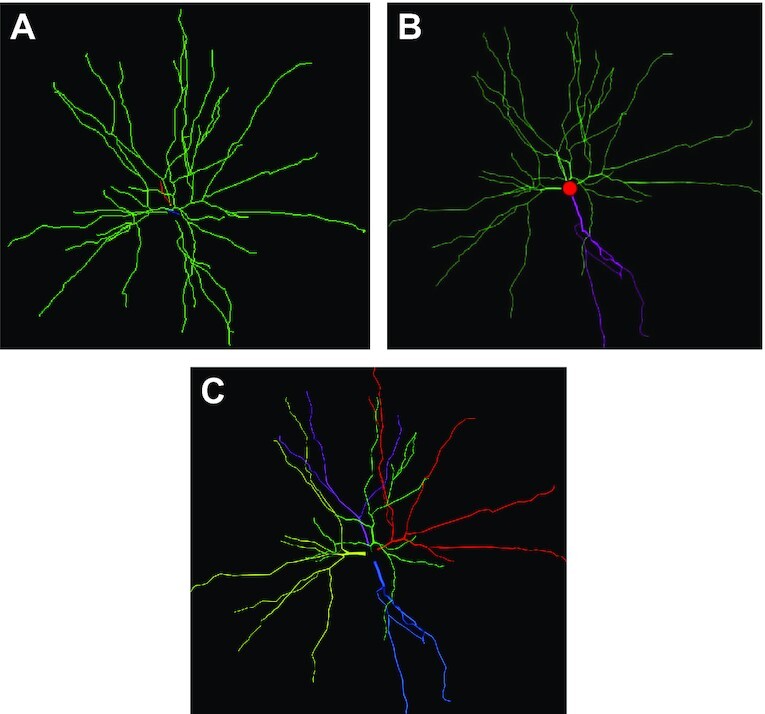
: Visualization of the reconstructed neuron (A) in NLMorphology Viewer, (B) on NeuroMorpho.org, and (C) with Qiber3D. Note that the single neuron in this example represents exactly 1 fiber in Qiber3D.

We used Qiber3D to visualize and measure a reconstructed neuron from a red-necked wallaby [35]. The published .swc file was obtained from NeuroMorpho.org[36]. We compared the 3D rendering of the neuron in Qiber3D with 2 other methods. The thickness of the structures was clearly visible in the Qiber3D visualization (Fig. 8C, Supplementary Movie S4), similar to the image on NeuroMorpho.org (Fig. 8B). In contrast, in the rendering with NLMorphology Viewer, a commonly used software tool to visualize neuron morphology, all fibers were displayed with the same diameter (Fig. 8B). The measurements from Qiber3D were in agreement with the published data on the NeuroMorpho.org website, as well as the output from NLMorphology Viewer (Table 2). The quantification of the total length in Qiber3D excludes the soma of the neuron, resulting in a slightly lowered output compared to the measurements with the other tools.

**Table 2.** Comparison of the quantitative output from the NeuroMorpho.org website, NLMorphology Viewer software, and Qiber3D

Parameter	NeuroMorpho.org	NLMorphology Viewer	Qiber3D
Branch points	30	30	30
Mean diameter (μm)	1.09	NA	1.38
Total length (μm)	5,097.48	5,046.92	4,991.83
Total volume (μm^3^)	6,362.05	6,347.60	6,288.30

NA: Not applicable.

## Conclusion

Here we present Qiber3D, a toolkit to visualize, reconstruct, and quantitatively analyze networks from 3D image stacks. Qiber3D combines the tools for a complete analytical workflow, from the raw image input to the morphometric quantification, within a highly configurable ecosystem. However, it can also be used in conjunction with other software packages and integrated into existing analysis pipelines. By applying a building block principle, Qiber3D is developed to be highly customizable and adaptable for a variety of input datasets. By default, Qiber3D offers 2 skeletonization algorithms to cover a variety of input network types. The thinning-based core method of this software package is suitable for reconstructing cell-derived as well as artificial fibrous networks. Additionally, 3D reconstruction based on the kimimaro implementation of the TEASAR algorithm [13,14] is possible in Qiber3D. The embedded visualization capability allows for the inspection of each image processing step to aid optimization of the image processing workflow. While the overall processing time is similar to manual processing, Qiber3D is designed to be used entirely hands-off to automate image analysis of large datasets. Running Qiber3D-based analysis on HPC clusters makes it suitable for high-throughput processing. Qiber3D’s test-driven design within the Python ecosystem allows for long-term evolution of the project. For example, integration with TensorFlow and PyTorch will be of interest in the future to apply machine-learning algorithms for computer vision tasks. In summary, Qiber3D is a versatile 3D image analysis toolkit that is accessible for a wide range of research questions.

## Methods

### Cell culture

Prostate microvascular cells (PrMECs) were obtained from ScienCell™ (Australian Biosearch, Wangara, WA, Australia) and expanded in endothelial cell medium (ECM) (Australian Biosearch, Wangara, WA, Australia). Cancer-associated fibroblasts (CAFs) were kindly provided by the Prostate Cancer Research Group, Department of Anatomy and Developmental Biology, Monash University [37]. The fibroblasts were cultured in RPMI 1640 medium (no phenol red) (Gibco, ThermoFisher Scientific, Scoresby, VIC, Australia) supplemented with 10% fetal bovine serum (Gibco, ThermoFisher Scientific, Scoresby, VIC, Australia), 1 nM testosterone (Sigma-Aldrich, CastleHill, NSW, Australia), 10 ng mL^−1^ human fibroblast growth factor 2 (FGF-2) (Miltenyi Biotec, Macquarie Park, NSW, Australia), 100 U penicillin, and 100 μg mL^−1^ streptomycin (Gibco, ThermoFisherScientific, Scoresby, VIC, Australia). All cells were maintained at 37°C in a humidified incubator containing 5% CO_2_, with media changes every 2–3 days.

### Preparation of hydrogel cultures

The 3D co-cultures were obtained using hydrogels composed of synthetic starPEG and maleimide-functionalized heparin as described previously [38,39]. Briefly, PrMECs and CAFs were seeded into hydrogels at a density of 6 × 10^6^ and 6 × 10^5^, respectively. Vascular endothelial growth factor (VEGF) (Peprotech, Lonza, MountWaverly, VIC, Australia), FGF-2, and stromal cell-derived factor 1 (SDF-1) (Miltenyi Biotec, Macquarie Park, NSW, Australia) were included into the gel at a concentration of 5 μg mL^−1^ each. Additionally, 2 mol of RGD-SP (H2N-GCWGGRGDSP-CONH2) were added to the gel. A molar ration of starPEG to heparin-maleimide of 1:0.75 was used to obtain a stiffness of ∼500 Pa (storage modulus). The starPEG-heparin hydrogels were maintained in ECM for 7 days at 37°C in a humidified incubator containing 5% CO_2_.

### Immunofluorescence of hydrogels

The cell-containing hydrogels were fixed in 4% (v/v) paraformaldehyde (Sigma-Aldrich, Castle Hill, NSW, Australia) for 45 min. Blocking and permeabilization were achieved by incubation with 5% goat serum (Gibco, ThermoFisher Scientific, Scoresby, VIC, Australia) and 0.1% Triton-X100 (Merck Millipore, Bayswater, VIC, Australia) in phosphate-buffered saline (PBS) for 2 h at room temperature. Primary antibody staining against the endothelial marker CD31 (cat No. bba7, R&D Systems; 1:200 in 1% goat serum) was performed overnight at 4°C. Subsequently, the samples were washed in 1% goat serum in PBS for 8 h with 3 changes of the washing buffer. Polyclonal goat anti-mouse IgG conjugated to Alexa-Fluor 488 (cat No. A11001, Invitrogen, ThermoFisher Scientific, Scoresby, VIC, Australia; 1:300) secondary antibody, Alexa-Fluor 633 conjugated Phalloidin (Invitrogen, ThermoFisher Scientific, Scoresby, VIC, Australia; 1:100), and 5 μg mL^−1^ 4′, 6-diamidino-2-phenylindole (DAPI) in 1% goat serum/PBS were applied overnight at 4°C. Images were acquired on a Nikon A1R inverted confocal microscope (Nikon Instruments Inc.; 10x, 1.32 × 1.32 μm px^−1^, *z*-step size 2.5 μm × 181). Image analysis was performed on the AlexaFlour-488 (green) channel of the acquired images to analyze the networks formed by the microvascular endothelial cells.

## Availability of Source Code and Requirements

- Project name: Qiber3D
- Project home page: https://github.com/theia-dev/Qiber3D
- Operating system(s): Platform independent
- Programming language: Python
- Other requirements: Python ≥3.7; for a list of required Python libraries, refer to the project’s requirements.txt
- License: MIT
- biotoolsID: qiber3D
- RRID:SCR_021790

## Data Availability

The raw images of the microvascular-like network are available as nd2 and tiff files at [40]. Supplementary Movies S1–S4 are also available on FigShare under [41], [42], [43] and [44] respectively. Snapshots of our code and other supporting data are openly available in the *GigaScience* repository, GigaDB [45].

## Additional Files

**Supplementary Figure S1**. Qiber3D’s image processing workflow with various combinations of optional steps in comparison to Fig. 6 in the main manuscript.

**Supplementary Movie S1**. Synthetic network

**Supplementary Movie S2**. Microvascular network

**Supplementary Movie S3**. Compare extraction steps

**Supplementary Movie S4**. Neuronal network

giab091_GIGA-D-21-00182_Original_Submission

giab091_GIGA-D-21-00182_Revision_1

giab091_GIGA-D-21-00182_Revision_2

giab091_GIGA-D-21-00182_Revision_3

giab091_Response_to_Reviewer_Comments_Revision_1

giab091_Reviewer_1_Report_Original_Submission Chris Armit -- 7/16/2021 Reviewed

giab091_Reviewer_2_Report_Original_Submission Lucas Daniel Lo Vercio, Ph.D. -- 7/19/2021 Reviewed

giab091_Reviewer_2_Report_Revision_1 Lucas Daniel Lo Vercio, Ph.D. -- 11/9/2021 Reviewed

giab091_Reviewer_3_Report_Original_Submission Alexandr Kalinin -- 7/26/2021 Reviewed

giab091_Reviewer_3_Report_Revision_1 Alexandr Kalinin -- 11/2/2021 Reviewed

giab091_Supplemental_File

## Abbreviations

CAF: cancer-associated fibroblasts; CPU: central processing unit; CT: computed tomography; DAPI: 6-diamidino-2-phenylindole; ECM: endothelial cell medium; FGF-2: human fibroblast growth factor 2; GUI: graphical user interface; HPC: high-performance computing; MPI: Message Passing Interface; MRI: magnetic resonance imaging; PBS: phosphate-buffered saline; PIMS: Python Image Sequence; PrMEC: prostate microvascular cell; PSF: point-spread function; QUT: Queensland University of Technology; RAM: random access memory; SDF-1: stromal cell-derived factor 1; TEASAR: Tree-structure Extraction Algorithm for Accurate and Robust skeletons; VEGF: vascular endothelial growth factor.

## Ethical Approval

All experiments involving human cells were approved by the Queensland University of Technology (QUT) Human Research Ethics Committee (Approval No. 1800000502).

## Competing Interests

The authors declare that they have no competing interests.

## Funding

A.J. was supported by a Postgraduate Research Award (International), QUT. L.B. was supported by a grant from the National Breast Cancer Foundation (PF-16-004) and acknowledges the support of grant 1159637 awarded through the 2018 Priority-driven Collaborative Cancer Research Scheme and co-funded by Cancer Australia and Leukemia Foundation of Australia. Some of the data reported in this work were obtained at the Central Analytical Research Facility (CARF) operated by the Institute for Future Environments, QUT. Access to CARF was supported by the Science and Engineering Faculty, QUT.

## Authors' Contributions

A.J. performed the experiments. H.E. and A.J. developed the toolkit. A.J. and H.E. analyzed and interpreted the data and wrote the manuscript. All authors read, edited, and approved the final manuscript.
