# Supplementary material for: Qiber3D—an open-source software package for the quantitative analysis of networks from 3D image stacks
Source: Gigascience. 2022 Feb 4;11:giab091. doi: 10.1093/gigascience/giab091 (PMC8848317; doi:10.1093/gigascience/giab091)
Supplement: giab091_GIGA-D-21-00182_Revision_1 [file giab091_giga-d-21-00182_revision_1.pdf]

GigaScience

Qiber3D - an open source software package for the quantitative analysis of networks from 3D image stacks

--Manuscript Draft--

Manuscript Number:	GIGA-D-21-00182R1
Full Title:	Qiber3D - an open source software package for the quantitative analysis of networks from 3D image stacks
Article Type:	Technical Note
Funding Information:	national breast cancer foundation (PF-16-004) — Dr Laura J Bray<br>cancer australia (1159637) — Dr Laura J Bray<br>leukemia foundation of australia (1159637) — Dr Laura J Bray
Abstract:	Background: Optical slice microscopy is commonly used to observe cellular morphology in 3D tissue culture, for example, the formation of cell-derived networks. The morphometric quantification of these networks is essential to study the cellular phenotype. Commonly, the quantitative measurements are performed on 2D projections of the image stack resulting in the loss of information in the third dimension. Currently available 3D image analysis tools rely on manual interactions with the software and are therefore not feasible for large datasets. Findings: Here we present Qiber3D, an open-source image processing toolkit. The software package includes the essential image analysis procedures required for image processing, from the raw image to the quantified data. Optional pre-processing steps can be switch on/off depending on the input data to allow for analyzing networks from a variety of sources. Two reconstruction algorithms are offered to meet the requirements for a wide range of network types. Furthermore, Qiber3D's rendering capabilities enable the user to inspect each step of the image analysis process interactively to ensure the creation of an optimal workflow for each application. Conclusions: Qiber3D is implemented as a Python package and its source code is freely available at https://github.com/theia-dev/Qiber3D . The toolkit was designed using a building block principle to enable the analysis a variety of structures, such as vascular networks, neuronal structures, or scaffolds from numerous input formats. While Qiber3D can be used interactively in the Python console, it is aimed at unsupervised automation to process large image datasets efficiently.
Corresponding Author:	Anna Jaeschke<br>Queensland University of Technology<br>Kelvin Grove, QLD AUSTRALIA
Corresponding Author Secondary Information:	
Corresponding Author's Institution:	Queensland University of Technology
Corresponding Author's Secondary Institution:	
First Author:	Anna Jaeschke
First Author Secondary Information:	
Order of Authors:	Anna Jaeschke<br>Hagen Eckert<br>Laura J Bray
Order of Authors Secondary Information:	
Response to Reviewers:	Dear Dr Nogoy.<br><br>We thank you once again for giving us the opportunity to revise and resubmit our

Powered by Editorial Manager® and ProduXion Manager® from Aries Systems Corporation

manuscript entitled: 'Qiber3D - an open source software package for the quantitative analysis of networks from 3D image stacks'. We have considered all the comments and have revised our manuscript accordingly. Please find below a detailed point-by-point response to the reviewers' comments and concerns. Moreover, we added the biotools and RRID identifiers to the manuscript to the 'Availability of source code and requirements' section. We look forward to your response to the revised manuscript at your earliest convenience.
Kind regards,
Dr Anna Jaeschke

Response to Reviewers

Reviewer #1
~~~~~~~~~~~

COMMENT: This interesting Technical Note describes the Qiber3D software tool, which is used for image analysis of 3D image stacks. There is a need to identify contiguous morphological components, such as alveolar airspaces and vascular networks, in 3D confocal image data and the Qiber3D toolkit was developed to address this issue. Importantly, the authors provide illustrative use case scenarios that include: 1) a synthetic example network; 2) a 3D reconstruction of the skeleton of a microvascular network from a confocal image stack of in vitro cultured mouse vascular cells, and; 3) a 3D reconstruction of a marsupial gigantopyramidal neuron. The software is publicly available from GitHub (https://github.com/theia-dev/Qiber3D) where it has been ascribed an Open Source Initiative-approved MIT license. In addition, test confocal image data of a 1GB vascular network used by Qiber3D is available on FigShare (https://doi.org/10.6084/m9.figshare.13655606.v1). These image data are of high quality and the Nikon ND2 files can be read using Fiji / ImageJ with the Bio-Formats plugin. Movies of the 3D reconstructions can be viewed using private sharing links.*

RESPONSE: We thank the reviewer for their helpful suggestions to clarify the processing steps.

COMMENT: Can the authors please describe the morphological operations used by Qiber3D? Specifically, I wish to know which morphological operations (erosion, dilation, etc.) were used by Qiber3D to improve segmentation of the contiguous vascular networks in the image stack of in vitro microvasculature that is shown in Figure 6 and Supplementary Figure S1.

RESPONSE: The section on *Morphological operations* (P4 L200) was rewritten for clarity. In the results section (P5 L326), we clarified the used morphological operations. We added a note to the supplementary Fig. S1 that it directly compares to Fig. 6 in the main manuscript.

COMMENT: I request that the movies of the 3D reconstructions are made publicly available on FigShare. The DOIs for the four movies should be included in the revised GigaScience manuscript.

RESPONSE: The videos are now referred to and linked using their public DOIs in the manuscript and the supplementary.

Reviewer #2
~~~~~~~~~~~

COMMENT: The authors are proposing an open software package for segmenting and quantifying networks in the biology domain. The article is well organized and they present two examples of usage.

RESPONSE: We thank the reviewer for their comments and helpful suggestions to improve the manuscript.

Powered by Editorial Manager® and ProduXion Manager® from Aries Systems Corporation

COMMENT: The authors state that the method works for different kind of biological networks. However, sometimes it is confusing in the text when they are describing the general problem and the particular problems. I recommend to carefully read and re-write for clarity. Maybe using a acronym when referring to general networks?

RESPONSE: We have re-read the manuscript and made sure to specify the type of network we were talking about. General networks of all kinds are referred to as “network”, special networks such as the microvascular, neuronal or - more general - cellular networks are now labelled as such.

COMMENT: Figure 1 is comprehensive to understand the general workflow. However, as the article is presenting a software tool, it must present a diagram that shows the architecture of the library, and how the existing libraries cooperate. An UML-like diagram like class diagram or component diagram is suggested

RESPONSE: The details of the inner workings/connections of Qiber3D are not suitable to be presented in a compact UML. We would argue that a figure representing a full UML representation of the toolkit goes beyond the scope of this article. We agree that the architecture needs to be well documented for Qiber3D to be used by the community. Therefore, we provide a detailed `source documentation <https://qiber3d.readthedocs.io/en/latest/documentation.html>` enabling users to integrate chosen parts of the toolkit tightly into their code.

COMMENT: In the GitHub repository, I only found pre-defined networks as examples. I strongly suggest to add an example with a multi-page tiff (or other 3D format), due to that is the output of confocal images (among other flurorescene microscopy methods). With processed and/synthetic networks, it is not possible to assess the capacity of the library to deal with this data.

RESPONSE: We added a multi-page TIFF variant of the sample file to figshare. As these images are large binary data files, we refrained from adding the files to the git repository. To simplify the interaction with the images, we extended the `in- and output documentation <https://qiber3d.readthedocs.io/en/latest/load_export.html>` of Qiber3D. Besides, we implemented a new *Example* class that simplifies the download of the different image variations. We also explain in the documentation how to directly download the files using the command line.

COMMENT: Line 93: “We included a method to create synthetic network images” Which method? Is an existing method?*

RESPONSE: The mentioned methode can convert a reconstructed network back in a layered image representation. We clarified this in the text (P2 L123)

COMMENT: Line 113: The statement about the median filtering is correct. However, a reference must be included to support it. For example: Loizou and Pattichis. Despeckle Filtering Algorithms and Software for Ultrasound Imaging .2008 3. Line 125: Add a citation to support that method to have an isotropic volume.*

RESPONSE: We thank the reviewer for the recommendation. The proposed reference (`10.2200/s00116ed1v01y200805ase001 <https://doi.org/10.2200/s00116ed1v01y200805ase001>`) is now included in the manuscript (P3 L167). We added also a citation (`10.1016/B978-012077790-7/50030-8 <https://doi.org/10.1016/B978-012077790-7/50030-8>`) that explains the use of cubic splines in image interpolations. (P3 L178)

COMMENT: Line 130: The gaussian filter homogenize neighbouring pixels. However, it diffuses the edges. The expression “more consistent boundaries” should be clarified.*

RESPONSE: The consistent boundaries referred to the binarisation step. The sentence was rewritten to reflect this better (P3 L188).

COMMENT: Line 145: it says “erosion and dilation […] to fill small holes”. I think that first a dilation and then the erosion is the correct order to perform that filling.*

Powered by Editorial Manager® and ProduXion Manager® from Aries Systems Corporation

RESPONSE: The section on *Morphological operations* (P4 L200) was rewritten to clarify the order of the operations.

COMMENT: Figure 6: The caption should clarify what image is being shown in the figure. I understand that are the microvascular cells.

RESPONSE: The caption for Fig. 6 was amended to include more detailed information on the shown network. (P5)

COMMENT: Line 365: I am confused here. Are the Proste microvascular cells or the Cancer-associated fibroblasts used in this article? I only found that the first one are shown.*

RESPONSE: While the image processing software is focused on analysing the networks form by the microvascular cells, the cell culture setup is a co-culture between microvascular cells and cancer-associated fibroblasts. The addition of fibroblasts to the cultures supports networks formation. However, the analysis was performed on images of cell stained for CD31, a marker specific for endothelial cells. Therefore, the fibroblasts are not visible in the sample image. The caption of Fig. 6 was rewritten to clarify this. (P5) Moreover, in the methods section the choice of the channel was clarified: "Image analysis was performed on the AlexaFlour-488 (green) channel of the acquired images to analyse the networks formed by the microvascular endothelial cells." (P7 L467)

COMMENT: Line 522: Year is missing in the reference.

RESPONSE: We added the year to the reference.

Reviewer #3
~~~~~~~~~~~

COMMENT: The paper presents Qiber3D, a new Python toolkit for the quantitative analysis of fiber-like structures in 3D microscopic images. The library includes a number of image preprocessing operations, two different methods for the network reconstruction, hierarchical feature extraction, as well as input/output (IO) and visualization capabilities. Provided examples include applications of Qiber3D to both synthetic and real-world data, demonstrating the ability of the toolbox to extract meaningful representations of different types of networks (microvascular, neuron morphology) and extract features that are close to previously known measures.*

While reviewing existing solutions for network quantification, the authors identified a few disadvantages: (1) most of these tools rely on 2D representations of 3D networks, which is not optimal; (2) most of the tools that support 3D data are often focused on the visualization aspect; (3) methods for processing and analysis steps are often available in different software packages, which makes building high-performance workflows for analyzing large datasets harder. The proposed solution is claimed to address these limitations.

The proposed solution is mostly implemented as a glue code for a number of Python libraries, including scikit-image for image processing, PIMS for IO, kimimaro for alternative skeletonization, NetworkX for building a graph representation of the network, etc. Therefore, the main contribution of this work is rather of an engineering type, as there are no contributions of novel methods or algorithms. However, in my opinion, the proposed toolbox does provide value for network analysis from 3D images in Python. The proposed solution enables straightforward construction of end-to-end pipelines from raw 3D image stack to tables of extracted features and visualizations.

While the paper is overall well-written and the methodological component of the approach is technically sound, the manuscript can be improved to strengthen the support for the claims made in the text.

RESPONSE: We thank the reviewer for their helpful advice to improve the software package and refine the manuscript.

Powered by Editorial Manager® and ProduXion Manager® from Aries Systems Corporation
~~~~~~~~~~~

COMMENT: While reviewing relevant tools and literature, the authors talk about disadvantages of proprietary tools or 3D open source packages that focus on visualization and then cite Fiji. It should be clarified that Fiji does not primarily focus on 3D visualization—in fact, it supports (almost) all image processing operations provided in Qiber3D and supports skeletonization and skeleton analysis via plugins [3]. It seems like almost the whole Qiber3D can be implemented in Fiji and automated via macros for high-performance processing of large datasets. Similarly, CellProfiler also supports (almost) all of these operations, including analyzing skeletons [4] and running workflows in a parallel mode (Distributed CellProfiler). It would be helpful to stress some benefits of building a network quantification pipeline using Qiber3D instead of one of these tools. For example, the authors could discuss in more detail the issues with Java/Fiji and HPC systems, or the rapidly growing ecosystem of Python tools for image analysis, an ability to run examples
in the cloud with a Jupyter notebook, or better accessibility of GPU-enabled libraries if further speed up is needed. This will help to
better identify the existing gap that Qiber3D is aiming to bridge and make its contributions to the community more clear.

RESPONSE: We revised the introduction to identify the existing gap and clarify the need for Qiber3D. (P1 L55)

COMMENT: Generally, when the main contribution of the paper is software, it is helpful if the authors identify some core design principles that they followed when developing this toolbox (e.g. flexibility vs. speed vs. ease-of-use, etc.). It helps the reader to follow the authors' thinking—how they dealt with the trade-offs they faced and design decisions they made during the process. For example, see discussions like this in [1] and [2].

RESPONSE: We included a section to introduce the design principles and goals of Qiber3D. (P2 L94)

COMMENT: The Findings (Implementation) section could benefit from better structure, because it reads more like a technical documentation when all operations are provided as a list. I suggest the authors group individual functions into topical subheading, e.g. "Image input", "Image pre-processing", "Segmentation", "Network reconstruction", etc. For example, it's confusing that the median filter and the Gaussian filter are separated by other operations.

RESPONSE: We thank the reviewer for their suggestion and changed the structure of the Findings section.

COMMENT: For available operations that have some parameters fixed, it would be great to provide justification for the chosen parameter values. For example, why rescaling to isotropic voxels support only upscaling in Z, but not downscaling in XY (to the larger voxel size, which can be useful for processing speed up when dealing with large structures that are well resolved). For filter operations, it'd be helpful to mention the choice of the filter shape (cube/ball) or the reasoning behind choosing parameters for merging jagged segments in the network optimization (e.g. do those depend on the size of the network?). Similarly for binarization, scikit-image supports at least 6 different methods for thresholding out-of-the-box (of which Li's and Triangle are often used for cell segmentation), so it is unclear why the authors only provide Otsu's method. I think making such decisions is fine, but the reasoning behind them should be made clear to the reader.

RESPONSE: In general, Qiber3D's is designed to be as flexible as possible for the user by providing the ability to configure parameters as required. We thank the reviewer for the suggestions to improve this aspect of the toolkit further.
Following the reviewer's argument, the target for the resampling can now be chosen to be either adjusting the Z-axis or the X/Y-plane resolution. We considered just a cubic footprint for the median filter, as for our typical small-sized filter (3x3x3), the shape was not significant. To make Qiber3D more customizable, the shape of the median filter can now be a cuboid or an arbitrary shape defined by a 3D NumPy array. The parameters for the raw network optimizations can now be altered in the configuration. This change

Powered by Editorial Manager® and ProduXion Manager® from Aries Systems Corporation

enables the adaption of the smoothing depending on the resolution of the images (see `documentation <https://qiber3d.readthedocs.io/en/latest/config.html#Qiber3D.config.extract.thinning>`). The Otsu thresholding method was chosen due to its good results with our research subject. We agree with the reviewer that this choice would not be optimal for some use cases. Therefore, the automatic threshold method can now be changed (see `documentation <https://qiber3d.readthedocs.io/en/latest/config.html#Qiber3D.config.extract.binary>`).

COMMENT: It is great that besides the thinning algorithm, the authors also provide TEASAR as an alternative. It would be great to see both of these algorithms applied to the same data to illustrate the difference between them. It is also not clear whether TEASAR was used for reconstruction in the Neuron morphology application.

RESPONSE: The microvascular and synthetic examples shown in the article are based on the thinning algorithms. We argue that the use of TEASAR on our example data would not be a fair comparison, as TEASAR is not optimised for cases that include loop structures. The neuron example is chosen to demonstrate the visualisation and analytics capacity of Qiber3D. Unfortunately, we were unable to access the original raw images that where used to create the network itself.

COMMENT: Although confocal microscopy eliminates most of the out-of-focus light, images still exhibit background noise and spherical aberrations. Axial smearing directly affects morphological analysis of 3D structures, from their relative locations to their volumes. Assessment of these effects can be made by computing FFT of the image or modeling the PSF of the microscope. As the authors note in their own recently published STAR Protocols paper [5], image deconvolution is an important pre-processing step to combat these artifacts. Therefore, I am surprised to not find the deconvolution module in Qiber3D, especially since open source Python implementations of the common deconvolution algorithms are available, e.g. in scikit-image (which is already a dependency) or in FlowDec [6]. If the authors have their reasons to not include deconvolution in Qiber3D, this decision should be discussed in the main text.

RESPONSE: After extensive testing while building Qiber3D, we concluded that deconvolution was not beneficial for our example data set and is probably not relevant for many users of this toolkit.

Two measures could be influenced by the PSF function of the microscope - fiber radius and position. The point-spread primarily manifests by elongating the objects in the image stacks along the z-axis. As the PSF is uniform over the image stack and the reconstruction functions find the center of the fibers, only a constant shift of the network along the x-axis is expected. Such a shift is without consequences for our purposes, as we have no outer frame of reference. The shortest distance for each object voxel to the background is measured to reconstruct the radius along the fibers. As the minimum is used, the x/y-plane with an often higher resolution becomes the dominant source for the radius definition. As the fibers are assumed to have a round cross-section, the
typical PSF function of the microscope has nearly no influence on the measured radii.

All in all, we think that the effort necessary to generate a high-quality PSF and the time to compute the deconvolution is not
required for most use cases. However, Qiber3D is built modularly and a deconvolution step can be added, using one of the many implementations available for Python.

COMMENT: Similarly, other popular methods for preprocessing include background removal and/or uneven illumination correction [7]. The authors may consider including them in the toolbox or mention why these are not often needed in network analysis pipelines.*

RESPONSE: Uneven illumination correction in the x/y plane was found to not be feasible for our dataset. The binarization step will remove changes in illumination over the frame. Moreover, with optical slicing, there is a chance to introduce artefacts by correcting uneven illumination in a slice-by-slice basis. In cases where this step is

Powered by Editorial Manager® and ProduXion Manager® from Aries Systems Corporation

	necessary, the user can make use of the Qiber3D's extensible nature and include uneven illumination correction as required for their dataset.<br>As with the deconvolution, we designed Qiber3D's backbone along our example data set and as minimal as possible. Qiber3D is extensible where required, but we think that every extra step/extension/algorithm needs to fit the input data. Therefore, we focused on a smaller selection of tools that we could test. Eventually, every image processing protocol should be adapted for the input data and required measurements.<br><br>**COMMENT:** It'd be great to have some example Jupyter notebooks that could be run in the cloud (with inline visualizations instead of opening a separate window).<br><br>**RESPONSE:** Qiber3D was extended to be compatible with Jupyter notebooks and to provide inline visualization. This functionality is now used to provide interactive sample sessions to introduce the capabilities of the toolkit. The notebooks can be directly run with the<br>help of the`MyBinder.org <https://mybinder.org/v2/gh/theia-dev/Qiber3D_jupyter/main?urlpath=git-pull%3Frepo%3Dhttps%253A%252F%252Fgithub.com%252Ftheia-dev%252FQiber3D%26urlpath%3Dtree%252FQiber3D%252Fdocs%252Fjupyter%252Findex.ipynb>`service. While it is an excellent way to start with Qiber3D, the strict resource limitations will make a local installation necessary for most applications.<br><br>**COMMENT:** Finally, I strongly encourage the authors to cite other open source tools that they used in Qiber3D [8]. They typically have the corresponding info on their GitHub page or in the documentation.<br><br>**RESPONSE:** We have added additional references to used software packages, either as a link in a footnote to the source code repository or to an appropriate scientific publication if available.<br><br>References: [1] Paszke, Adam, et al. "Pytorch: An imperative style, high-performance deep learning library." Advances in neural information processing systems. 2019. [2] Buslaev, Alexander, et al. "Albumentations: fast and flexible image augmentations." Information 11.2 (2020): 125. [3] https://imagej.net/plugins/analyze-skeleton/ [4] https://cellprofiler-manual.s3.amazonaws.com/CellProfiler-4.0.4/modules/measurement.html?highlight=skeleton*measureimageskeleton [5] Bonda, Ulrich, et al. "3D Quantification of Vascular-Like Structures in z Stack Confocal Images." STAR protocols 1.3 (2020): 100180. [6] https://github.com/hammerlab/flowdec [7] Singh, Shantanu, et al. "Pipeline for illumination correction of images for high-throughput microscopy." Journal of microscopy 256.3 (2014): 231-236. [8] https://ilovesymposia.com/2019/05/02/why-you-should-cite-open-source-tools/
**Additional Information:**	
**Question**	**Response**
Are you submitting this manuscript to a special series or article collection?	No
**Experimental design and statistics**<br><br>Full details of the experimental design and statistical methods used should be given in the Methods section, as detailed in our Minimum Standards Reporting Checklist. Information essential to interpreting the data presented should be made available in the figure legends.	Yes

Have you included all the information requested in your manuscript?	
**Resources**<br><br>A description of all resources used, including antibodies, cell lines, animals and software tools, with enough information to allow them to be uniquely identified, should be included in the Methods section. Authors are strongly encouraged to cite Research Resource Identifiers (RRIDs) for antibodies, model organisms and tools, where possible.<br><br>Have you included the information requested as detailed in our Minimum Standards Reporting Checklist?	Yes
**Availability of data and materials**<br><br>All datasets and code on which the conclusions of the paper rely must be either included in your submission or deposited in publicly available repositories (where available and ethically appropriate), referencing such data using a unique identifier in the references and in the "Availability of Data and Materials" section of your manuscript.<br><br>Have you have met the above requirement as detailed in our Minimum Standards Reporting Checklist?	Yes

Manuscript Click here to access/download;Manuscript;2020_Qiber3D.pdf

Placeholder for OUP logo oup.pdf

GigaScience, 2017, 1–9

doi: xx.xxxx/xxxx
Manuscript in Preparation
Technical Note

TECHNICAL NOTE

Qiber3D - an open source software package for the quantitative analysis of networks from 3D image stacks

Anna Jaeschke[1,2,3*,†], Hagen Eckert[4,†] and Laura J. Bray[1,2,5]

[1]Centre for Biomedical Technologies, Queensland University of Technology (QUT), Kelvin Grove, Australia and [2]School of Mechanical, Medical and Process Engineering, Science and Engineering Faculty, Queensland University of Technology (QUT), Brisbane, Australia and [3]Mechanobiology Institute, National University of Singapore, Singapore and [4]Department of Mechanical Engineering and Materials Science, Duke University, Durham, NC, USA and [5]ARC Training Centre for Cell and Tissue Engineering Technologies, Queensland University of Technology (QUT), Kelvin Grove, Australia

*anna.jaeschke@connect.qut.edu.au
†Contributed equally.

Abstract

Background: Optical slice microscopy is commonly used to observe cellular morphology in 3D tissue culture, for example, the formation of cell-derived networks. The morphometric quantification of these networks is essential to study the cellular phenotype. Commonly, the quantitative measurements are performed on 2D projections of the image stack resulting in the loss of information in the third dimension. Currently available 3D image analysis tools rely on manual interactions with the software and are therefore not feasible for large datasets. **Findings:** Here we present `Qiber3D`, an open-source image processing toolkit. The software package includes the essential image analysis procedures required for image processing, from the raw image to the quantified data. Optional pre-processing steps can be switch on/off depending on the input data to allow for analyzing networks from a variety of sources. Two reconstruction algorithms are offered to meet the requirements for a wide range of network types. Furthermore, `Qiber3D`'s rendering capabilities enable the user to inspect each step of the image analysis process interactively to ensure the creation of an optimal workflow for each application. **Conclusions:** `Qiber3D` is implemented as a Python package and its source code is freely available at https://github.com/theia-dev/Qiber3D. The toolkit was designed using a building block principle to enable the analysis a variety of structures, such as vascular networks, neuronal structures, or scaffolds from numerous input formats. While `Qiber3D` can be used interactively in the Python console, it is aimed at unsupervised automation to process large image datasets efficiently.

Key words: morphometric quantification; confocal imaging; image processing; vascular networks; fibrous networks; neurons

Background

The process of angiogenesis, the development of new blood vessels from the existing vasculature, is the center of numerous research questions. Studying the processes involved in vessel formation, maturation and remodeling is essential for a better understanding of normal development and angiogenesis-related disease stages [1, 2]. *In vitro* angiogenesis models aim towards replicating the formation of vascular-like networks in the laboratory [2]. Optical slice microscopy is commonly used to follow vessel formation in *in vitro* angiogenesis models [3]. Thereby, multiple images are acquired across different positions in the z-plane throughout the specimen capturing the cell morphology in 3D [3]. The vascular phenotype can be assessed by qualitative observation or by morphometric quantification of fiber length, number of fibers, cross-sectional area or volume as well as branching [2]. The quantitative characterization of the morphological phenotype is an essential tool to study cellular responses. Currently, most

Compiled on: October 30, 2021.
Draft manuscript prepared by the author.

morphometric measurement approaches rely on 2D projections, often maximum intensity projections, of the 3D images. However, 2D quantification of 3D structures limits the accuracy of data obtained and results in the loss of relevant information in the third dimension [4]. Consequently, there is a need for quantification tools of 3D image files that can be adapted to various areas of research studying networks composed of elongated or fiber-like structures.

Computational approaches exist to visualize and investigate cell morphology in 2D and 3D. Proprietary software, for example Amira™ (ThermoFisher Scientific) [5], Imaris (Oxford Instruments) or Metamorph® (Molecular Devices) is capable of 3D, 4D and 5D image processing and analysis. However, proprietary software packages are often black boxes tailored to machines sold by the same companies. While the documentation usually covers the fundamental methodology of a function, the actual implementation is not revealed. Regularly, these software packages are designed to be standalone all-in-one products, making their automated integration into analysis protocols cumbersome. Furthermore, the licensing expenses restrict accessibility to these software packages and therefore significantly limit the transferability and reproducibility of protocols using them. A multitude of open-source image processing software packages, that are capable of 3D image visualization and processing, have been developed in response [6, 7, 8]. Many of these tools are widely extensible by the use of plugins [6, 9]. Thereby, software that was not specifically developed for processing image stacks, such as ImageJ/Fiji [9], can be utilized for 3D image analysis.

Available 3D quantification protocols often combine existing software packages, and usually require manual handling, at least for parts of the image analysis workflow [10, 11, 12]. Besides carrying the risk of user-based subjectivity, it also limits the throughput of samples for experiments with large image datasets. In some cases, switching between multiple existing software packages is necessary [12], making the image processing time- and resource-consuming and therefore, again, not feasible for large datasets.

Automation, at least for parts of the image analysis workflow, can be achieved through external scripts or, in the case of ImageJ/Fiji [9], by using macros. While this is a feasible route for smaller datasets, the automation of image processing tasks using tools designed primarily for a graphical user interface (GUI) is limited. These limitations become especially obvious if one aims at utilizing high-performance computing (HPC) clusters or cloud computing resources. While the use of these tools on shared computing resources is challenging, running them without a GUI (headless) and unsupervised for a prolonged time requires extensive effort. Overall, it is impractical to design an unsupervised automated workflow that can quantify 3D structures in bulk with the available graphical image analysis tools.

Here we present `Qiber3D` an open-source software package for morphometric quantification of networks from 3D image stacks. `Qiber3D` combines the required tools for a complete analytical workflow, from the raw image to final measured values. The core method of `Qiber3D` for the 3D reconstruction of networks is based on thinning. While this approach covers many applications, for example vascular-like networks or scaffolds, we also offer the kimimaro implementation of the Tree-structure Extraction Algorithm for Accurate and Robust skeletons (TEASAR) [13, 14] as an alternative skeletonization method. With the implementation of two reconstruction modes, `Qiber3D` is usable for the quantification of a variety of networks from image stacks.

`Qiber3D` generates a graph representation of a network based on a variety of input formats. Interactive inspection of the network at each step of the workflow assists with the optimization of image processing parameters. The extracted quantitative morphometric data can be exported in a multitude of options to provide broad compatibility with other software. The implementation as an open-source Python package creates a highly customizable program that is suitable for image analysis automation and tight integration into existing workflows. By design, `Qiber3D` is suitable for applying general batch distribution approaches to be used on HPC clusters enabling high-throughput image analysis for large datasets.

Findings

Design Principles

`Qiber3D` is designed to quantify a large number of network image stacks without manual user intervention. To achieve this goal, we realized the toolkit within the Python ecosystem. The access to the wide selection of open-source modules, such as *SciPy* [15] or *scikit-image* [16], enabled us to build upon a well-maintained foundation. As the Python language is widely used in the scientific community, `Qiber3D` can be easily included as a building block into new and existing image analysis workflows. Using a Jupyter [17] notebook as an easy platform to develop new workflows directly on a shared computing resource, will help to familiarize with `Qiber3D` quickly and enable collaborative work. We provide example Jupyter notebooks[1] as part of the documentation. Moreover, with the growing interest in machine-learning algorithms for computer vision tasks, the straightforward integration with toolkits such as TensorFlow [18] and PyTorch [19] provides an additional advantage.

`Qiber3D` provides the tools for a complete analytical workflow, from the raw image input to the morphometric quantification. Aiming for high customizability, we provide a streamlined way to configure the various parameters used in `Qiber3D`. Optional steps can be included or excluded from the image processing pipeline (Fig.1) allowing for `Qiber3D` to be applied on raw as well as preprocessed images from a variety of sources. The open-source nature of the software allows for researchers to taylor it to the requirements of their datasets if necessary. Furthermore, open access to the source code avoids analytical blackboxes and enables long-term evolution of the project.

`Qiber3D`'s test-driven design allows for well-structured collaborative development. As the size of experimental image stacks restricts their usage for integrated testing, we included a method to create *synthetic* network images. This method takes a reconstructed network as input and renders it as a layered 3D image that can subsequently be stored in the desired format. This allows for proper unit tests of the source code without the need to download large datasets.

`Qiber3D` is developed as a command line tool enabling smooth integration into existing workflows as well as automated, high-throughput images analysis. However, visualization is achieved using `vedo`[2], allowing the user to interact with the image output at different stages during image processing.

Implementation

Data IO

As interoperability is an essential goal of the `Qiber3D` toolkit, a wide variety of import and export options is paramount. Confocal images are usually acquired using commercial imaging platforms and the image files are saved in a proprietary file format, containing the metadata. `Qiber3D`s support for multi-dimensional image formats is based on `PIMS`[3] (Python Image Sequence). This choice allows the use of essential image formats like `.tiff`-stacks as well as proprietary file formats from microscope vendors like Leica, Nikon,

1 https://github.com/theia-dev/Qiber3D_jupyter
2 https://github.com/marcomusy/vedo
3 https://github.com/soft-matter/pims

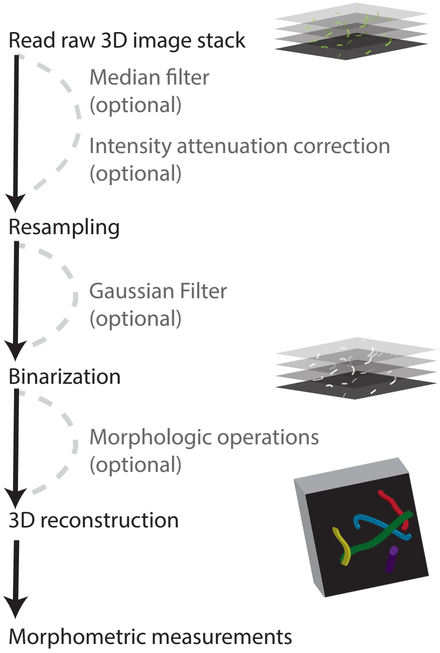

Figure 1. `Qiber3D`'s pipeline combines the required image processing steps for 3D morphometric quantification of networks. Optional tools are provided to cover a range of image content.

Olympus, and Zeiss as input. Physical size information (the voxel size) and, for multi-channel images, the channel of interest for network reconstruction is provided upon image loading or set as configuration variable for automated workflows. For some file formats, `Qiber3D` is able to extract the required metadata directly from the input file. Besides loading 3D image stacks to create the *Network* object, it can be built from files describing the network. `Qiber3D` supports the MicroVisu3D format `.mv3d`, traditionally used for vascular networks, as well as the `.swc` and the `.ntr` format, popular for neuronal networks.

The internal representation of the `Qiber3D` network can be stored as a binary file (`.qiber`) that allows for fast loading of the reconstructed network into the software. Easy visualization in web applications, and the import into specialized rendering software like `Blender` is achieved by saving the 3D representation as a collection of truncated cones in the `.x3D` file format. Moreover, `Qiber3D` supports several human-readable formats. The spatial data of the reconstructed network can be exported as `.mv3d`, `.swc` and `.csv` files. When exporting to a `.json` or Microsoft Excel `.xlsx` file format using *openpyxl*[4], the complete set of metadata and calculated properties is included. Furthermore, the network can be exported as a 3D `.tiff` image stack.

Image pre-processing

Median filter (optional). The primary purpose of the 3D median filter, also known as the despeckle filter, is the removal of speckles and extrema [20]. The value of each voxel is replaced by the median of its surrounding voxels. By default, a three voxels wide neighborhood is used. However, this size can be modified in the configuration depending on the noise present in the image.

Intensity attenuation correction (optional). In 3D confocal images, light absorption can cause a decrease in signal intensity in slices located deeper into the sample. An exponential curve is fitted to the average intensities I_A in each of the slices to their physical stack position z to correct for this intensity attenuation (Fig. 2).

$$I_A = a\exp(bz) \tag{1}$$

The optimal parameters a and b for the intensity correction are determined using a non-linear least-squares fit.

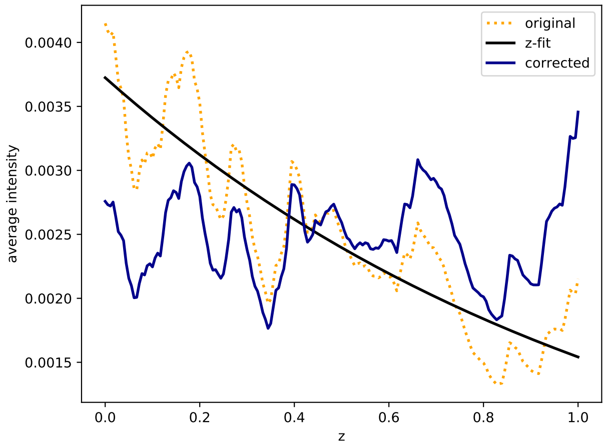

Figure 2. Intensity attenuation correction in the example image of the microvascular network. Yellow - original signal. Blue - corrected signal. Black - intensity fit.

Resampling to an isotropic voxel size. Commonly, the x/y resolution of image stacks differs from the resolution along the z-axis. As a cubic voxel size is beneficial to optimize the subsequent image processing steps, the z-axis or the x/y-plane of the image is resampled to a uniform resolution using a third-order spline interpolation [21].

Gaussian filter (optional). The image stack is blurred with a Gaussian filter simultaneously in all three dimensions to minimize the effect of noise on the image segmentation by reducing sharp differences between neighboring pixels. Applying a Gaussian filter reduces the noise level and imaging artifacts significantly. As the values now change smoothly from the outside to the inside of a structure, a border created by a cutoff will be more consistent and less rough.

Image segmentation

Binarization. The grayscale image is reduced to a binary representation to locate the boundaries of the structures and to label the segments. All voxels that are equal to or greater than a threshold are set to True and all others to False. A dynamic threshold calculation for each stack is performed permitting an automated workflow. By default, Otsu thresholding, an unsupervised, nonparametric method that tries to maximize the separability of the resultant classes (exactly two in the binary image), by utilizing the zeroth- and first-order moments of the histogram [22], is applied. Other thresholding algorithms can be selected, depending on the image. Alternatively, the threshold can be set directly as a percentage value of the signal intensity.

Morphological operations (optional). The obtained structures in the binarized image stack might not be perfectly solid, depending on the quality of the input data. A combination of dilation steps followed by an equal number of erosion steps fills small holes and compacts the segments' surface. The number of steps is configurable. In this section, small islands caused by imaging artifacts can also be removed based on a threshold set by the user.

4 https://foss.heptapod.net/openpyxl/openpyxl

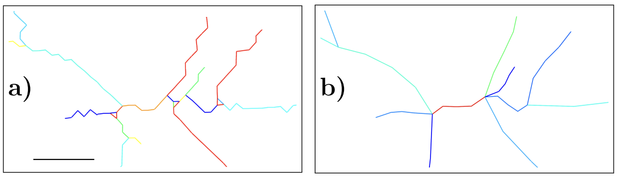

Figure 3. Network optimization. After thinning (a), the network is optimized by replacing tiny segments with more extensive structures and smoothing out voxel artifacts (b). Scale bar: 12.3 µm (10 voxel)

Network reconstruction

Reconstruction by thinning (default). The default network reconstruction approach is based on thinning, a morphological operation to remove selected foreground pixels from binary images. Initially, the image stack is distance transformed and every foreground (*True*) voxel in the stack is assigned the shortest Euclidean distance to a background (*False*) voxel. Subsequently, the Lee-Kashyap algorithm [23] is applied to extract the medial axis, and the binary image is reduced to its skeleton. The remaining foreground voxels, the skeleton, are modeled as a graph using *NetworkX* [24], defined by vertices that are connected by edges. Each foreground voxel represents a vertex, and connecting edges are formed between neighboring voxel. A radius is assigned to each vertex based on the earlier distance transformation. To form *Segments* (see below for details), the graph is reduced to contain only vertices that represent end and branch points.

Distinctive edges are often formed along with branch points, sharp bends, or on the network's rim. Such edges occur between vertices that are direct neighbors and the resulting path is particularly jagged (Fig 3 a). This resolution artifact results in an overestimation of the fiber length and volume and an inflated branch point count. To mitigate these drawbacks, edges that are shorter than six voxel are merged with larger neighbors or removed if isolated and each edge is interpolated using a cubic spline (Fig. 3 b). New points are generated by default at a rate of approximately one point every ten voxel. All edges are fit to a spline with at least five points.

Reconstruction with TEASAR (alternative). Initially, the TEASAR method aimed to generate organ centerlines from 3D imaging generated by MRI, or CT scans [13, 14]. It has been used in a variety of applications, from pore networks in clay rocks [25, 26], to neuronal networks [27, 28] since. `Qiber3D` incorporates the kimimaro[5] implementation of the TEASAR algorithm that was developed to skeletonize neurons. For processing networks that resemble neuronal structures, that is branching of structures (dendrites) from a cell body (soma), the use of this method is recommended over the thinning-based reconstruction. The output of the skeletonization step is a connected graph, from which we extract the quantitative measurements of the network.

Morphometric measurement

In `Qiber3D` the reconstructed network is represented in a hierarchical structure (Fig. 4). We use the terms *Network*, *Fiber*, and *Segments* to describe the components of the reconstruction. Note that these expressions are purely used conceptually to label `Qiber3D`'s output and that the terms might not refer to the actual structure. A *Fiber* might be a real fiber, an elongated cell, or another object depending on the application.

The largest entity is the *Network*, which represents the entirety of the structure. It is composed of a collection of *Fibers*, that are formed by connected *Segments*, the smallest elements. A *Segment* is described by a collection of sorted points stored along the corresponding radius. The vertices between the points are interpreted as truncated cones. *Segments* end when they reach a branch point (grey points, Fig. 4). Therefore, *Segments* themselves are never branched. A branch point belongs to all *Segments* that it connects.

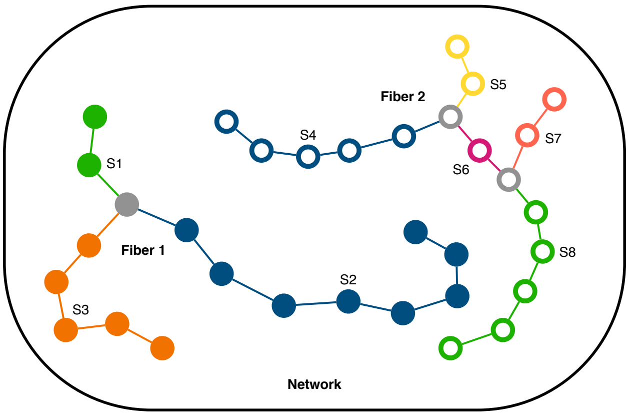

Figure 4. Qiber3D's hierarchical structure. *Segments* S1-S3 generate *Fiber 1* (filled points) and segments S4-S8 *Fiber 2* (hollow points), forming the *Network*. Branch points are colored in gray.

Each element, on the different hierarchical levels, is defined by a unique identifier and several quantitative properties, for example, the volume or the average radius. The average radius can be misleading considering that the distance between the points forming an element can be non-uniform resulting in a skewed measurement. Therefore, we included the notion of a length-weighted cylindrical radius and return the radius of a cylinder with the same volume and length as the element of interest. While the modelling of the volume as overlapping truncated cones is sufficient in most cases, an improved volume estimation can be obtained from the rasterized network. As the start and endpoint of a *Fiber* within a given 3D image stack is interchangeable, the directional data is analyzed based on the assumption that all *Fibers* are pointing upwards (positive z-axis). Depending on the application, *Fibers* can be convoluted and the orientation of the *Segments* can be more meaningful in some cases. In both cases, the orientation of each element is described using the azimuth and altitude regarding a half-sphere.

For the *Network* additional measurements, like the number of *Fibers*, *Segments* and branch points, or the bounding box volume, are provided. The *Network* object also stores the relevant metadata of the input.

Visualization

`Qiber3D` uses `vedo`, a lightweight python module, that is based on `VTK` [29] and `numpy` [30], to visualize the network in 3D. The embedded rendering capability allows the users to quickly inspect a network by rotating the camera view and zoom into regions of interest. A linked view of the different reconstruction steps and the resulting skeleton enables the user to examine them in relation to each other. The network's color can be customized to represent different properties of the network, such as fiber length, volume, or average radius. In addition to the interactive visualization, 3D views can be exported as static images or animations.

Results

To provide a comprehensive overview of the features, `Qiber3D` was applied to the synthetic example image as well as two experimental datasets, an *in vitro* microvascular network and a neuron that was reconstructed elsewhere.

5 `https://github.com/seung-lab/kimimaro`

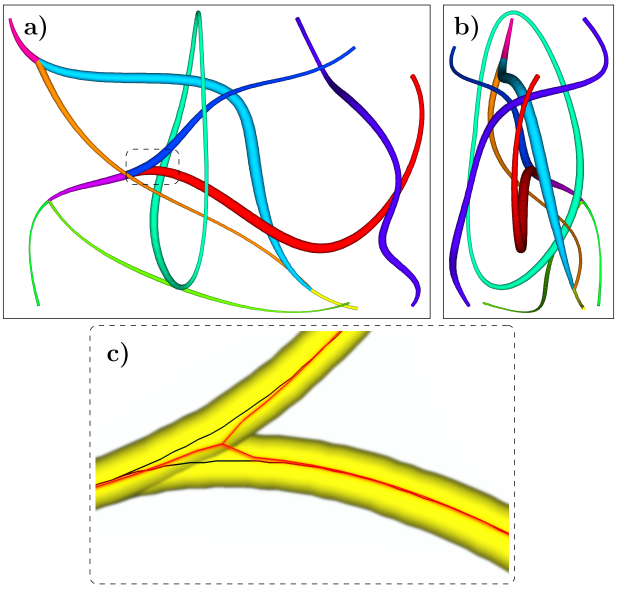

Figure 5. Synthetic network example with a) view on the x/y-plane and b) view on the z/y-plane. c) A branch point of the synthetic network with the original (black) and reconstructed (red) centerlines.

Synthetic example image

The output of the synthetic example image is presented in Fig. 5 and Suppl. Movie 1[6]. The synthetic example network was visualized in 3D and the segments composing the fibers were observed (Fig. 5 a). The measurements of the synthetic network reconstructed with `Qiber3D` were in agreement with the input data (Tab. 1). Interestingly, the branch points of the fibers were slightly displaced (Fig. 5 c) without affecting the measured total volume of the synthetic network (Tab. 1). This discrepancy is due to the thickness of the fibers concealing the original merging points during reconstruction.

Table 1. Comparison of the synthetic network with the output of Qiber3D after reconstruction.

	synthetic network	`Qiber3D` output
Number of fibers	4	4
Total length [µm]	1141.44	1120.84
Total Volume [μm^3]	4688.67	4665.62
Average radius [µm]	0.94	0.96
Cylinder radius [µm]	1.14	1.15

Microvascular network

`Qiber3D` was used to analyze a confocal image of a cellular network derived from microvascular cells grown *in vitro* (Fig. 6 a).

The analysis was performed including all optional procedures of the workflow (Fig. 6). The application of the median filter resulted in a clearer image with fewer extrema (Fig. 6 b). Upon correction of the intensity attenuation, the signal distribution was found more equal along the z axis (compare Fig. 6 b and c, lower panels). The quantitative observation was confirmed by the distribution of the mean signal intensity slice along the z axis before (Fig. 2, blue line) and after (Fig. 2, orange line) the correction step. If the z-drop correction was switched off, the vessels in the lower part of the image were lost after reconstruction of the microvascular network (Suppl. Fig. 1 b, d-f). Following the intensity attenuation correction, a Gaussian filter resulted in noise reduction and smoothing of the

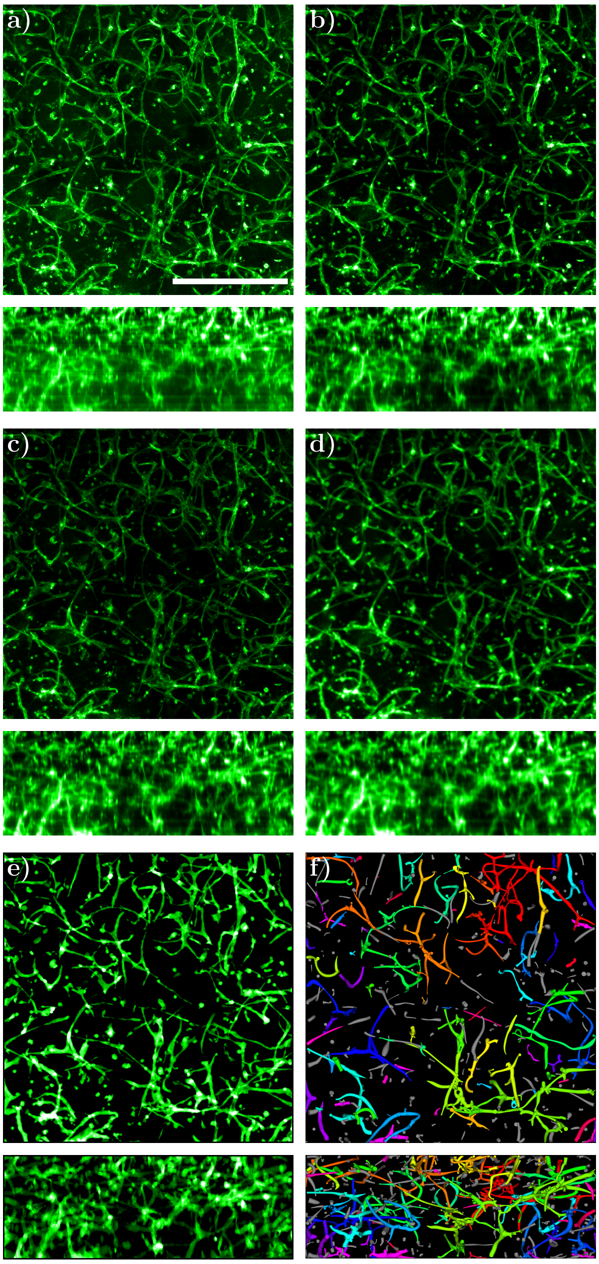

Figure 6. `Qiber3D`'s image processing workflow. An image of each step is shown as a average intensity projection along the z-axis (upper panels) and along the x-axis (lower panels). a) Raw image. Green: AlexaFluor 488-staining of CD31, a surface marker specific for endothelial cells. Scale bar: 500 µm. b) Image after median filter. c) Image corrected for intensity attenuation (z-drop correction). d) Image after Gaussian blur and surface compacting. e) Binarized image. f) Reconstructed microvascular network.

boundaries (Fig. 6 d). After pre-processing the image using the optional filters, image segmentation was performed. Morphological operations, in the form of a combination of dilation and erosion (each with five iterations) and the removal of islands smaller than 100 μm^3, were applied to the binary image (Fig. 6 e). Ommiting the morphological operations prior to reconstruction, resulted in the presence of numerous small particles that were not connected to the microvascular network ('islands') (Suppl. Fig. 1 c-e). Finally, the skeleton of the microvascular network was successfully reconstructed from the 3D image stack (Fig. 6 f, Suppl. Movie 2[7]). Each step was visualized interactively while processing the input image

6 Suppl. Movie 1 - Synthetic Network 10.6084/m9.figshare.13633802

7 Suppl. Movie 2 - Microvascular network 10.6084/m9.figshare.13633805

and compared together afterwards (Suppl. Movie 3[8]). Removing the optional filter steps for the image of the microvascular-like network led to artifacts in the reconstructed network (Suppl. Fig. 1 b, e-f).

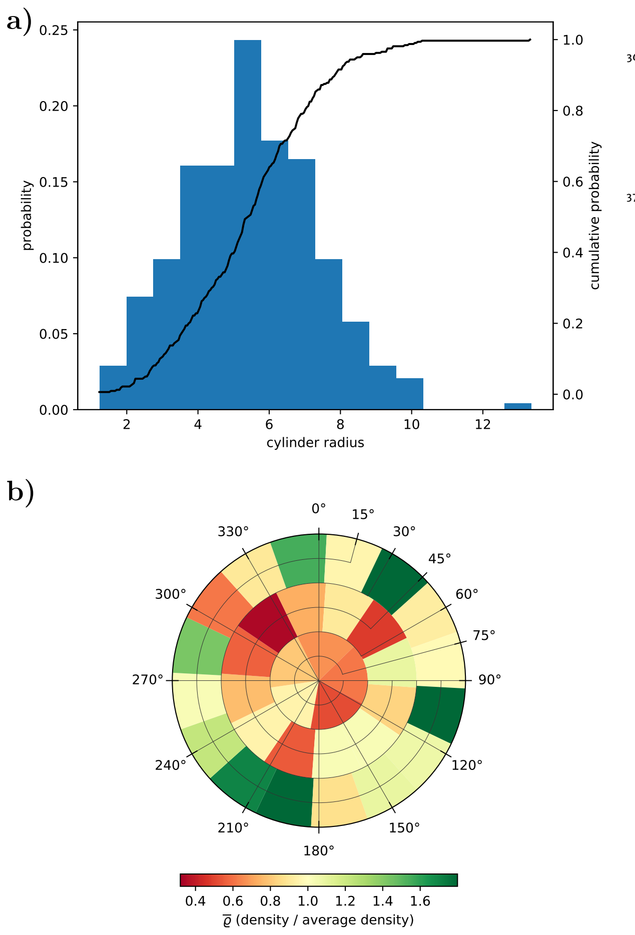

Figure 7. Graphical output of quantitative data from the microvascular network in Qiber3D. a) Distribution of the cylinder radius of the fibers within the network. b) Orientation distribution of the fibers in 3D.

The distribution of network attributes can be visualized in `Qiber3D` in the form of a histogram. In Fig. 7 a the distribution of the cylinder radius in the cellular network is presented as an example. The fiber radii were normally distributed between 1 and 10 µm with an average at 6.2 µm. To visualize the directional distribution in 3D, we introduced a spherical histogram. In Fig. 7 b every bin represents a part of a half-sphere. The start point for every network fiber was considered to be at the center of the half-sphere. The segments of each fiber were averaged into a single vector that captures the fiber's dominant direction. As the surface area of the different bins of a half-sphere are not perfectly equal, the number of intersecting vectors were divided by the surface area of the bin. Furthermore, the fiber density of each bin was scaled using the average fiber density over the half-sphere to allow for a streamlined comparisons between multiple networks. The color scale indicates the scaled fiber density. For the microvascular network, the majority of fibers are located parallel to the x/y-axis (Fig. 7 b).

Processing a 1 GB nd2 file with `Qiber3D` on an Intel Core i7-6700 machine with 16 GB RAM running a Windows 10 (64-bit) operation system took approximately 7.5 minutes. Manual analyzing a similar image takes approximately 8.5 min, not considering the time to switch between various software packages [12]. While this is a slight decrease in processing time of one image, `Qiber3D` can be applied to numerous images without user interaction making it suitable to analyze large datasets. As `Qiber3D` is designed to run on a single CPU, running multiple processes of `Qiber3D` in parallel will accelerate the average image processing time for large datasets significantly. The use of build-in multiprocessing tools in Python enables straightforward implementation of parallel processing. For larger deployments on HPC clusters, task management using Message Passing Interface (MPI) for Python enables the analysis of vast image datasets. The implementation of `Qiber3D` as a Python package enables the smooth integration with other Python libraries to build customized tools that meet the requirements of varying computational environments, e.g. different HPC centers.

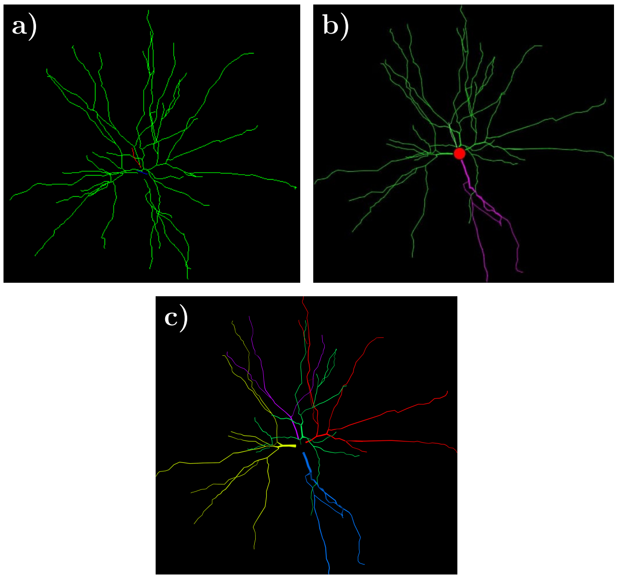

Figure 8. Visualization of the reconstructed neuron in a) NLMorphology Viewer, on b) NeuroMorph.org and c) with Qiber3D. Note, that the single neuron in this example represents exactly one fiber in Qiber3D.

Neuron morphology

We used `Qiber3D` to visualize and measure a reconstructed neuron from a red-necked wallaby [31]. The published `.swc` file was obtained from NeuroMorph.org. We compared the 3D rendering of the neuron in `Qiber3D` with two other methods. The thickness of the structures was clearly visible in the `Qiber3D` visualization (Fig. 8 c, Suppl. Movie 4[9]) similar to the image on NeuroMorph.org (Fig. 8 b). In contrast, in the rendering with NLMorphology Viewer, a commonly used software tool to visualize neuron morphology, all fibers were displayed with the same diameter (Fig. 8 b). The measurements from `Qiber3D` were in agreement with the published data on the NeuroMorph.org website as well as the output from NLMorphology Viewer (Tab. 2). The quantification of the total length in `Qiber3D` excludes the soma of the neuron resulting in a slightly lowered output compared to the measurements with the other tools.

Conclusion

Here we present `Qiber3D`, a toolkit to visualize, reconstruct and quantitatively analyze networks from 3D image stacks. `Qiber3D`

8 Suppl. Movie 3 - Compare extraction steps 10.6084/m9.figshare.13633799

9 Suppl. Movie 4 - Neuronal network 10.6084/m9.figshare.13633823

Table 2. Comparison of the quantitative output from the NeuroMorph.org website, NLMorphology Viewer software, and Qiber3D.

	NeuroMorph	NLMorphology Viewer	`Qiber3D`
Branch points	30	30	30
Average Diameter [µm]	1.09	na	1.38
Total length [µm]	5097.48	5046.92	4991.83
Total Volume [µm^3]	6362.05	6347.60	6288.30

combines the tools for a complete analytical workflow, from the raw image input to the morphometric quantification, within a highly configurable ecosystem. However, it can also be used in conjunction with other software packages, and integrated into existing analysis pipelines. By applying a building block principle, `Qiber3D` is developed to be highly customizable and adaptable for a variety of input datasets. By default, `Qiber3D` offers two skeletonization algorithms to cover a variety of input network types. The thinning-based core method of this software package is suitable for reconstructing cell-derived as well as artificial fibrous networks. Additionally, 3D reconstruction based on the kimimaro implementation of the TEASAR algorithm [13, 14] is possible in `Qiber3D`. The embedded visualization capability allows for the inspection of each image processing step to aid optimization of the image processing workflow. While the overall processing time is similar to manual processing, `Qiber3D` is designed to be used fully hands-off to automate image analysis of large datasets. Running `Qiber3D`-based analysis on high performance computing clusters makes it suitable for high-throughput processing. `Qiber3D`'s test-driven design within the Python ecosystem allows for long-term evolution of the project. For example, integration with TensorFlow and PyTorch will be of interest in the future to apply machine-learning algorithms for computer vision tasks. In summary, `Qiber3D` is a versatile 3D image analysis toolkit that is accessible for a wide range of research questions.

Methods

Cell culture

Prostate microvascular cells (PrMECs) were obtained from SciencCell™ (Australian Biosearch, Wangara, WA, Australia) and expanded in endothelial cell medium (ECM) (Australian Biosearch, Wangara, WA, Australia). Cancer-associated fibroblasts (CAFs) were kindly provided by the Prostate Cancer Research Group, Department of Anatomy and Developmental Biology, Monash University [32]. The fibroblasts were cultured in RPMI 1640 media (no phenol red) (Gibco, ThermoFisherScientific, Scoresby, VIC, Australia) supplemented with 10 % fetal bovine serum (FBS) (Gibco, ThermoFisherScientific, Scoresby, VIC, Australia), 1 nM testosterone (Sigma-Aldrich, CastleHill, NSW, Australia), 10 ng mL^{-1} FGF-2 (MiltenyiBiotec, MacquariePark, NSW, Australia), 100 U penicillin, and 100 µg mL^{-1} streptomycin (Gibco, ThermoFisherScientific, Scoresby, VIC, Australia). All cells were maintained at 37 °C in a humidified incubator containing 5 % CO_2, with media changes every 2-3 days.

Preparation of hydrogel cultures

3D co-cultures were obtained using hydrogels comprised of synthetic starPEG and maleimide-functionalised heparin as described previously [33, 34]. Briefly, PrMECs and CAFs were seeded into hydrogels at a density of 6x10^6 and 6x10^5, respectively. Vascular endothelial growth factor (VEGF) (Peprotech, Lonza, MountWaverly, VIC, Australia), human fibroblast growth factor 2 (FGF-2), and stromal cell-derived factor 1 (SDF-1) (MiltenyiBiotec, MacquariePark, NSW, Australia) were included into the gel at a concentration of 5 µg mL^{-1} each. Additionally, 2 mol of RGD-SP (H2N-GCWGGRGDSP-CONH2) were added to the gel. A molar ration of starPEG to heparin-maleimide of 1:0.75 was used to obtain a stiffness of approximately 500 Pa (storage modulus). The starPEG-heparin hydrogels were maintained in ECM for 7 days at 37 °C in a humidified incubator containing 5 % CO^2.

Immunofluorescence of hydrogels

The cell-containing hydrogels were fixed in 4 % (v/v) paraformaldehyde (PFA) (Sigma-Aldrich, CastleHill, NSW, Australia) for 45 min. Blocking and permeabilisation was achieved by incubation with 5 % goat serum (Gibco, ThermoFisherScientific, Scoresby, VIC, Australia) and 0.1 % Triton-X100 (MerckMillipore, Bayswater, VIC, Australia) in phosphate-buffered-saline (PBS) for 2 h at room temperature. Primary antibody staining against the endothelial marker CD31 (cat no. bba7, R&D Systems; 1:200 in 1 % goat serum) was performed overnight at 4 °C. Subsequently, the samples were washed in 1 % goat serum in PBS for 8 h with three changes of the washing buffer. Polyclonal goat anti-mouse IgG conjugated to Alexa-Fluor 488 (cat no. A11001, Invitrogen, ThermoFisherScientific, Scoresby, VIC, Australia; 1:300) secondary antibody, Alexa-Fluor 633 conjugated Phalloidin (Invitrogen, ThermoFisherScientific, Scoresby, VIC, Australia; 1:100), and 5 µg mL^{-1} 4',6-diamidino-2-phenylindole (DAPI) in 1 % goat serum/PBS were applied overnight at 4 °C. Images were acquired on a Nikon A1R inverted confocal microscope (Nikon Instruments Inc.; 10x, 1.32 µm px^{-1} x 1.32 µm px^{-1}, z-step size 2.5 µm x 181). Image analysis was performed on the AlexaFlour-488 (green) channel of the acquired images to analyze the networks formed by the microvascular endothelial cells.

Availability of source code and requirements

- Project name: Qiber3D
- Project home page: https://github.com/theia-dev/Qiber3D
- Operating system(s): Platform independent
- Programming language: Python
- Other requirements: Python $\geq$ 3.7, for a list of required Python libraries, refer to the project's requirements.txt
- License: MIT
- biotoolsID: qiber3D
- RRID: SCR_021790

Availability of supporting data and materials

The raw images of the microvascular-like network is available as `nd2` and `tif` files at doi:10.6084/m9.figshare.13655606.

Declarations

List of abbreviations

CAF Cancer-associated fibroblasts
CT computed tomography
DAPI 6-diamidino-2-phenylindole
ECM endothelial cell medium
FBS fetal bovine serum
FGF-2 human fibroblast growth factor 2
GUI graphical user interface

HPC high-performance computing
MPI Message Passing Interface
MRI Magnetic resonance imaging
PBS Phosphate-buffered saline
PFA paraformaldehyde
SDF-1 stromal cell-derived factor 1
TEASAR Tree-structure Extraction Algorithm for Accurate and Robust skeletons
VEGF Vascular endothelial growth factor

Ethical Approval (optional)

All experiments involving human cells were approved by the Queensland University of Technology Human Research Ethics Committee (Approval number: 1800000502).

Consent for publication

not applicable

Competing Interests

The authors declare that they have no competing interests.

Funding

AJ was supported by a Postgraduate Research Award (International), QUT. LB was supported by a grant from the National Breast Cancer Foundation (PF-16-004) and acknowledges the support of grant 1159637 awarded through the 2018 Priority-driven Collaborative Cancer Research Scheme and co-funded by Cancer Australia and Leukemia Foundation of Australia. Some of the data reported in this work were obtained at the Central Analytical Research Facility (CARF) operated by the Institute for Future Environments, QUT. Access to CARF was supported by the Science and Engineering Faculty, QUT.

Author's Contributions

AJ performed the experiments. HE and AJ developed the toolkit. AJ and HE analyzed and interpreted the data, and wrote the manuscript. AJ, HE and LJB read, edited and approved the final manuscript.

Acknowledgements

HE acknowledges the Duke University, Center for Autonomous Materials Design, for computational support and Prof. Stefano Curtarolo for fruitful discussions.

References

1. Carmeliet P, Jain RK. Angiogenesis in Cancer and Other Diseases. Nature 2000;407(6801):249–257.
2. Staton CA, Reed MWR, Brown NJ. A Critical Analysis of Current in Vitro and in Vivo Angiogenesis Assays. International Journal of Experimental Pathology 2009;90(3):195–221.
3. Conchello JA, Lichtman JW. Optical Sectioning Microscopy. Nature Methods 2005;2(12):920–931.
4. Rytlewski JA, Geuss LR, Anyaeji CI, Lewis EW, Suggs LJ. Three-Dimensional Image Quantification as a New Morphometry Method for Tissue Engineering. Tissue Engineering Part C, Methods 2012;18(7):507.
5. Stalling D, Westerhoff M, Hege HC. Amira: A Highly Interactive System for Visual Data Analysis. In: Visualization Handbook Elsevier; 2005.p. 749–767.
6. Peng H, Ruan Z, Long F, Simpson JH, Myers EW. V3D Enables Real-Time 3D Visualization and Quantitative Analysis of Large-Scale Biological Image Data Sets. Nature Biotechnology 2010;28(4):348–353.
7. Fedorov A, Beichel R, Kalpathy-Cramer J, Finet J, Fillion-Robin JC, Pujol S, et al. 3D Slicer as an Image Computing Platform for the Quantitative Imaging Network. Magnetic resonance imaging 2012;30(9):1323–1341.
8. Eliceiri KW, Berthold MR, Goldberg IG, Ibáñez L, Manjunath BS, Martone ME, et al. Biological Imaging Software Tools. Nature Methods 2012;9(7):697–710.
9. Schindelin J, Arganda-Carreras I, Frise E, Kaynig V, Longair M, Pietzsch T, et al. Fiji: An Open-Source Platform for Biological-Image Analysis. Nature Methods 2012;9(7):676–682.
10. Lee E, Takahashi H, Pauty J, Kobayashi M, Kato K, Kabara M, et al. A 3D in Vitro Pericyte-Supported Microvessel Model: Visualisation and Quantitative Characterisation of Multistep Angiogenesis. Journal of Materials Chemistry B 2018;6(7):1085–1094.
11. Nishiguchi A, Matsusaki M, Kano MR, Nishihara H, Okano D, Asano Y, et al. In Vitro 3D Blood/Lymph-Vascularized Human Stromal Tissues for Preclinical Assays of Cancer Metastasis. Biomaterials 2018;179:144–155.
12. Bonda U, Jaeschke A, Lighterness A, Baldwin J, Werner C, De-Juan-Pardo EM, et al. 3D Quantification of Vascular-Like Structures in z Stack Confocal Images. STAR Protocols 2020;p. 100180.
13. Sato M, Bitter I, Bender MA, Kaufman AE, Nakajima M. TEASAR: Tree-Structure Extraction Algorithm for Accurate and Robust Skeletons. In: Proceedings the Eighth Pacific Conference on Computer Graphics and Applications IEEE Comput. Soc; 2000. p. 281–449.
14. Bitter I, Kaufman AE, Sato M. Penalized-distance volumetric skeleton algorithm. IEEE Transactions on Visualization and Computer Graphics 2001;7(3):195–206.
15. Virtanen P, Gommers R, Oliphant TE, Haberland M, Reddy T, Cournapeau D, et al. SciPy 1.0: Fundamental Algorithms for Scientific Computing in Python. Nature Methods 2020;17:261–272.
16. van der Walt S, Schönberger JL, Nunez-Iglesias J, Boulogne F, Warner JD, Yager N, et al. scikit-image: image processing in Python. PeerJ 2014;2:e453.
17. Kluyver T, Ragan-Kelley B, Pérez F, Granger B, Bussonnier M, Frederic J, et al. Jupyter Notebooks - a publishing format for reproducible computational workflows. In: Positioning and Power in Academic Publishing: Players, Agents and Agendas IOS Press; 2016.p. 87–90.
18. Abadi M, Barham P, Chen J, Chen Z, Davis A, Dean J, et al. TensorFlow: A System for Large-Scale Machine Learning. In: Proceedings of the 12th USENIX Conference on Operating Systems Design and Implementation USENIX Association; 2016. p. 265–283.
19. Paszke A, Gross S, Massa F, Lerer A, Bradbury J, Chanan G, et al. PyTorch: An Imperative Style, High-Performance Deep Learning Library. In: Wallach H, Larochelle H, Beygelzimer A, d'Alché-Buc F, Fox E, Garnett R, editors. Advances in Neural Information Processing Systems 32 Curran Associates, Inc.; 2019.p. 8024–8035.
20. Loizou CP, Pattichis CS. Despeckle Filtering Algorithms and Software for Ultrasound Imaging. In: Synth. Lect. Algorithms Softw. Eng., vol. 1; 2008.p. 1–166.
21. Thévenaz P, Blu T, Unser M. Image Interpolation and Resampling. In: Handbook of Medical Imaging Elsevier; 2000.p. 393–420.
22. Otsu N. A Threshold Selection Method from Gray-Level His-

tograms. IEEE Transactions on Systems, Man, and Cybernetics 1979;9(1):62–66.
23. Lee TC, Kashyap RL, Chu CN. Building Skeleton Models via 3-D Medial Surface Axis Thinning Algorithms. CVGIP: Graphical Models and Image Processing 1994;56(6):462–478.
24. Hagberg AA, Schult DA, Swart PJ. Exploring Network Structure, Dynamics, and Function using NetworkX. In: Proceedings of the 7th Python in Science Conference; 2008. p. 11 – 15.
25. Keller LM, Holzer L, Wepf R, Gasser P. 3D geometry and topology of pore pathways in Opalinus clay: Implications for mass transport. Applied Clay Science 2011;52(1-2):85–95.
26. Song Y, Davy CA, Troadec D, Blanchenet AM, Skoczylas F, Talandier J, et al. Multi-scale pore structure of COx claystone: Towards the prediction of fluid transport. Marine and Petroleum Geology 2015;65:63–82. Cited By 46.
27. Evers JF, Schmitt S, Sibila M, Duch C. Progress in Functional Neuroanatomy: Precise Automatic Geometric Reconstruction of Neuronal Morphology From Confocal Image Stacks. Journal of Neurophysiology 2005;93(4):2331–2342.
28. ya Takemura S, Bharioke A, Lu Z, Nern A, Vitaladevuni S, Rivlin PK, et al. A visual motion detection circuit suggested by Drosophila connectomics. Nature 2013;500(7461):175–181.
29. Schroeder W, Martin K, Lorensen B, Kitware I. The Visualization Toolkit: An Object-oriented Approach to 3D Graphics. Kitware; 2006.
30. Harris CR, Millman KJ, van der Walt SJ, Gommers R, Virtanen P, Cournapeau D, et al. Array programming with NumPy. Nature 2020;585(7825):357–362.
31. Jacobs B, Garcia ME, Shea-Shumsky NB, Tennison ME, Schall M, Saviano MS, et al. Comparative morphology of gigantopyramidal neurons in primary motor cortex across mammals. Journal of Comparative Neurology 2018;526(3):496–536.
32. Lawrence MG, Taylor RA, Toivanen R, Pedersen J, Norden S, Pook DW, et al. A Preclinical Xenograft Model of Prostate Cancer Using Human Tumors. Nature Protocols 2013;8(5):836–848.
33. Tsurkan MV, Chwalek K, Prokoph S, Zieris A, Levental KR, Freudenberg U, et al. Defined Polymer-Peptide Conjugates to Form Cell-Instructive starPEG-Heparin Matrices In Situ. Advanced Materials 2013;25(18):2606–2610.
34. Bray LJ, Binner M, Holzheu A, Friedrichs J, Freudenberg U, Hutmacher DW, et al. Multi-Parametric Hydrogels Support 3D in Vitro Bioengineered Microenvironment Models of Tumour Angiogenesis. Biomaterials 2015;53:609–620.

Click here to access/download
Supplementary Material
2020_Qiber3D_suppl.pdf
