# Supplementary material for: Qiber3D—an open-source software package for the quantitative analysis of networks from 3D image stacks
Source: Gigascience. 2022 Feb 4;11:giab091. doi: 10.1093/gigascience/giab091 (PMC8848317; doi:10.1093/gigascience/giab091)
Supplement: giab091_GIGA-D-21-00182_Revision_3 [file giab091_giga-d-21-00182_revision_3.pdf]

GigaScience

Qiber3D - an open-source software package for the quantitative analysis of networks from 3D image stacks

--Manuscript Draft--

**Manuscript Number:**	GIGA-D-21-00182R3
**Full Title:**	Qiber3D - an open-source software package for the quantitative analysis of networks from 3D image stacks
**Article Type:**	Technical Note
**Funding Information:**	national breast cancer foundation (PF-16-004) — Dr Laura J Bray<br>cancer australia (1159637) — Dr Laura J Bray<br>leukemia foundation of australia (1159637) — Dr Laura J Bray
**Abstract:**	Background: Optical slice microscopy is commonly used to observe cellular morphology in 3D tissue culture, for example, the formation of cell-derived networks. The morphometric quantification of these networks is essential to study the cellular phenotype. Commonly, the quantitative measurements are performed on 2D projections of the image stack resulting in the loss of information in the third dimension. Currently available 3D image analysis tools rely on manual interactions with the software and are therefore not feasible for large datasets. Findings: Here we present Qiber3D, an open-source image processing toolkit. The software package includes the essential image analysis procedures required for image processing, from the raw image to the quantified data. Optional pre-processing steps can be switched on/off depending on the input data to allow for analyzing networks from a variety of sources. Two reconstruction algorithms are offered to meet the requirements for a wide range of network types. Furthermore, Qiber3D's rendering capabilities enable the user to inspect each step of the image analysis process interactively to ensure the creation of an optimal workflow for each application. Conclusions: Qiber3D is implemented as a Python package and its source code is freely available at https://github.com/theia-dev/Qiber3D . The toolkit was designed using a building block principle to enable the analysis a variety of structures, such as vascular networks, neuronal structures, or scaffolds from numerous input formats. While Qiber3D can be used interactively in the Python console, it is aimed at unsupervised automation to process large image datasets efficiently.
**Corresponding Author:**	Anna Jaeschke<br>Queensland University of Technology<br>Kelvin Grove, QLD AUSTRALIA
**Corresponding Author Secondary Information:**	
**Corresponding Author's Institution:**	Queensland University of Technology
**Corresponding Author's Secondary Institution:**	
**First Author:**	Anna Jaeschke
**First Author Secondary Information:**	
**Order of Authors:**	Anna Jaeschke<br>Hagen Eckert<br>Laura J Bray
**Order of Authors Secondary Information:**	
**Response to Reviewers:**	The requested changes were made (reference included)
**Additional Information:**	

Powered by Editorial Manager® and ProduXion Manager® from Aries Systems Corporation

Question	Response
Are you submitting this manuscript to a special series or article collection?	No
**Experimental design and statistics**<br><br>Full details of the experimental design and statistical methods used should be given in the Methods section, as detailed in our Minimum Standards Reporting Checklist. Information essential to interpreting the data presented should be made available in the figure legends.<br><br>Have you included all the information requested in your manuscript?	Yes
**Resources**<br><br>A description of all resources used, including antibodies, cell lines, animals and software tools, with enough information to allow them to be uniquely identified, should be included in the Methods section. Authors are strongly encouraged to cite Research Resource Identifiers (RRIDs) for antibodies, model organisms and tools, where possible.<br><br>Have you included the information requested as detailed in our Minimum Standards Reporting Checklist?	Yes
**Availability of data and materials**<br><br>All datasets and code on which the conclusions of the paper rely must be either included in your submission or deposited in publicly available repositories (where available and ethically appropriate), referencing such data using a unique identifier in the references and in the "Availability of Data and Materials" section of your manuscript.	Yes

Powered by Editorial Manager® and ProduXion Manager® from Aries Systems Corporation

Have you have met the above requirement as detailed in our Minimum Standards Reporting Checklist?	

Manuscript Click here to access/download;Manuscript;2020_Qiber3D.pdf

GigaScience, 2017, 1–9

doi: xx.xxxx/xxxx
Manuscript in Preparation
Technical Note

TECHNICAL NOTE

Qiber3D - an open-source software package for the quantitative analysis of networks from 3D image stacks

Anna Jaeschke[1,2,3*,†], Hagen Eckert[4,†] and Laura J. Bray[1,2,5]

[1]Centre for Biomedical Technologies, Queensland University of Technology (QUT), Kelvin Grove, Australia and [2]School of Mechanical, Medical and Process Engineering, Science and Engineering Faculty, Queensland University of Technology (QUT), Brisbane, Australia and [3]Mechanobiology Institute, National University of Singapore, Singapore and [4]Department of Mechanical Engineering and Materials Science, Duke University, Durham, NC, USA and [5]ARC Training Centre for Cell and Tissue Engineering Technologies, Queensland University of Technology (QUT), Kelvin Grove, Australia

[*]anna.jaeschke@connect.qut.edu.au
[†]Contributed equally.

Abstract

Background: Optical slice microscopy is commonly used to observe cellular morphology in 3D tissue culture, for example, the formation of cell-derived networks. The morphometric quantification of these networks is essential to study the cellular phenotype. Commonly, the quantitative measurements are performed on 2D projections of the image stack resulting in the loss of information in the third dimension. Currently available 3D image analysis tools rely on manual interactions with the software and are therefore not feasible for large datasets. **Findings:** Here we present `Qiber3D`, an open-source image processing toolkit. The software package includes the essential image analysis procedures required for image processing, from the raw image to the quantified data. Optional pre-processing steps can be switched on/off depending on the input data to allow for analyzing networks from a variety of sources. Two reconstruction algorithms are offered to meet the requirements for a wide range of network types. Furthermore, `Qiber3D`'s rendering capabilities enable the user to inspect each step of the image analysis process interactively to ensure the creation of an optimal workflow for each application. **Conclusions:** `Qiber3D` is implemented as a Python package and its source code is freely available at `https://github.com/theia-dev/Qiber3D`. The toolkit was designed using a building block principle to enable the analysis a variety of structures, such as vascular networks, neuronal structures, or scaffolds from numerous input formats. While `Qiber3D` can be used interactively in the Python console, it is aimed at unsupervised automation to process large image datasets efficiently.

Key words: morphometric quantification; confocal imaging; image processing; vascular networks; fibrous networks; neurons

Background

The process of angiogenesis, the development of new blood vessels from the existing vasculature, is the center of numerous research questions. Studying the processes involved in vessel formation, maturation and remodeling is essential for a better understanding of normal development and angiogenesis-related disease stages [1, 2]. *In vitro* angiogenesis models aim towards replicating the formation of vascular-like networks in the laboratory [2]. Optical slice microscopy is commonly used to follow vessel formation in *in vitro* angiogenesis models [3]. Thereby, multiple images are acquired across different positions in the z-plane throughout the specimen capturing the cell morphology in 3D [3]. The vascular phenotype can be assessed by qualitative observation or by morphometric quantification of fiber length, number of fibers, cross-sectional area or volume, as well as branching [2]. The quantitative characterization of the morphological phenotype is an essential tool to study cellular responses. Currently, most

Compiled on: December 3, 2021.
Draft manuscript prepared by the author.

morphometric measurement approaches rely on 2D projections, often maximum intensity projections, of the 3D images. However, 2D quantification of 3D structures limits the accuracy of data obtained and results in the loss of relevant information in the third dimension [4]. Consequently, there is a need for quantification tools of 3D image files that can be adapted to various areas of research studying networks composed of elongated or fiber-like structures.

Computational approaches exist to visualize and investigate cell morphology in 2D and 3D. Proprietary software, for example Amira™ (ThermoFisher Scientific) [5], Imaris (Oxford Instruments) or Metamorph® (Molecular Devices) is capable of 3D, 4D and 5D image processing and analysis. However, proprietary software packages are often black boxes tailored to machines sold by the same companies. While the documentation usually covers the fundamental methodology of a function, the actual implementation is not revealed. Regularly, these software packages are designed to be standalone all-in-one products, making their automated integration into analysis protocols cumbersome. Furthermore, the licensing expenses restrict accessibility to these software packages and therefore significantly limit the transferability and reproducibility of protocols using them. A multitude of open-source image processing software packages capable of 3D image visualization and processing, have been developed in response [6, 7, 8]. Many of these tools are widely extensible by the use of plugins [6, 9]. Thereby, software that was not specifically developed for processing image stacks, such as ImageJ/Fiji [9], can be utilized for 3D image analysis.

Available 3D quantification protocols often combine existing software packages, and usually require manual handling, at least for parts of the image analysis workflow [10, 11, 12]. Besides carrying the risk of user-based subjectivity, it also limits the throughput of samples for experiments with large image datasets. In some cases, switching between multiple existing software packages is necessary [12], making the image processing time- and resource-consuming and, therefore, again, not feasible for large datasets.

Automation, at least for parts of the image analysis workflow, can be achieved through external scripts or, in the case of ImageJ/Fiji [9], by using macros. While this is a feasible route for smaller datasets, the automation of image processing tasks using tools designed primarily for a graphical user interface (GUI) is limited. These limitations become especially obvious if one aims at utilizing high-performance computing (HPC) clusters or cloud computing resources. While the use of these tools on shared computing resources is challenging, running them without a GUI (headless) and unsupervised for a prolonged time requires extensive effort. Overall, it is impractical to design an unsupervised automated workflow that can quantify 3D structures in bulk with the available graphical image analysis tools.

Here we present `Qiber3D` an open-source software package for morphometric quantification of networks from 3D image stacks. `Qiber3D` combines the required tools for a complete analytical workflow, from the raw image to final measured values. The core method of `Qiber3D` for the 3D reconstruction of networks is based on thinning. While this approach covers many applications, for example, vascular-like networks or scaffolds, we also offer the kimimaro implementation of the Tree-structure Extraction Algorithm for Accurate and Robust skeletons (TEASAR) [13, 14] as an alternative skeletonization method. With the implementation of two reconstruction modes, `Qiber3D` is usable for the quantification of a variety of networks from image stacks.

`Qiber3D` generates a graph representation of a network based on a variety of input formats. The option to inspect the network interactively at each step of the workflow assists in optimizing the image processing parameters. The extracted quantitative morphometric data can be exported in a multitude of options to provide broad compatibility with other software. The implementation as an open-source Python package creates a highly customizable program suitable for image analysis automation and tight integration into existing workflows. By design, `Qiber3D` is suitable for applying general batch distribution approaches to be used on HPC clusters enabling high-throughput image analysis for large datasets.

Findings

Design Principles

`Qiber3D` is designed to quantify a large number of network image stacks without manual user intervention. To achieve this goal, we realized the toolkit within the Python ecosystem. The access to the wide selection of open-source modules, such as *SciPy* [15] or *scikit-image* [16], enabled us to build upon a well-maintained foundation. As the Python language is widely used in the scientific community, `Qiber3D` can be easily included as a building block into new and existing image analysis workflows. Using a Jupyter [17] notebook as an easy platform to develop new workflows directly on a shared computing resource, will help to familiarize with `Qiber3D` quickly and enable collaborative work. Moreover, with the growing interest in machine-learning algorithms for computer vision tasks, the straightforward integration with toolkits such as TensorFlow [18] and PyTorch [19] provides an additional advantage. An often-cited drawback of using Python is the speed limitation compared to compiled languages. Python code needs to be interpreted at runtime and is therefore not optimized for the hardware it is running on. Memory usage needs to be considered with large input data sets, as the native Python datatypes can be inefficient. These limitations are mitigated by the fact that most scientific routines utilized in `Qiber3D` are written in C or Fortran and compiled for the CPU architecture.

`Qiber3D` provides the tools for a complete analytical workflow, from the raw image input to the morphometric quantification. Aiming for high customizability, we provide a streamlined way to configure the various parameters used in `Qiber3D`. Optional steps can be included or excluded from the image processing pipeline (Fig.1) allowing for `Qiber3D` to be applied on raw as well as preprocessed images from a variety of sources. We focused the software's backbone on a selected set of tools that we could test extensively using the data sets available to us. Specific research questions and the nature of the input data may demand custom steps/extensions/algorithms. As we cannot anticipate such requirements, we choose to design `Qiber3D` as compact as possible. Eventually, every image processing protocol should be adapted for the input data and required measurements. While deconvolution and planar illumination correction are commonly used in image processing, they are not included in `Qiber3D`. During the design and testing of `Qiber3D`, we concluded that deconvolution was not beneficial for our example data set and is probably not relevant for many users of this toolkit. Two measures can be influenced by the point-spread function (PSF) of the microscope - fiber radius and position. The point-spread primarily manifests by elongating the objects in the image stacks along the z-axis. As the PSF is uniform over the image stack and the reconstruction functions find the center of the fibers, only a constant shift of the network along the x-axis is expected. Such a shift is without consequences for our purposes, as we have no outer frame of reference. The radius along the fibers is measured by the shortest distance for each central voxel to the background. As the minimum is used, the x/y-plane with an often higher resolution becomes the dominant source for the radius definition. The typical PSF of the microscope has nearly no influence on the measured radii as the fibers are assumed to have a circular cross-section. All in all, we think that the effort necessary to generate a high-quality PSF and the time to compute the deconvolution is not required for most use cases. Uneven illumination correction in the x/y-plane was not suitable for our testing data. Slight changes in the illumination over

the plane are evened out by the binarization step already. Moreover, there is a chance to introduce artifacts by correcting uneven illumination on a slice-by-slice basis. In cases where these steps are unavoidable, `Qiber3D` can be extended utilizing the many implementations of image processing tasks readily available in Python. Overall, the open-source nature of the software avoids analytical blackboxes and allows for researchers to tailor it to their data.

`Qiber3D`'s test-driven design allows for well-structured collaborative development. As the size of experimental image stacks restricts their usage for integrated testing, we included a method to create *synthetic* network images. This method takes a reconstructed network as input and renders it as a layered 3D image that can subsequently be stored in the desired format. This allows for proper unit tests of the source code without the need to download large datasets. All in all, the open-source approach combined with the test-driven design enables the long-term evolution of the project through user contributions.

`Qiber3D` is developed as a command-line tool enabling smooth integration into existing workflows as well as automated, high-throughput images analysis. We are aware that building `Qiber3D` as a command-line tool results in a higher barrier to entry. `Qiber3D` and its documentation is designed to ease the transition into using command-line tools. Moreover, visualization using `vedo`[1], allows the user to interact with the image output at different stages during image processing.

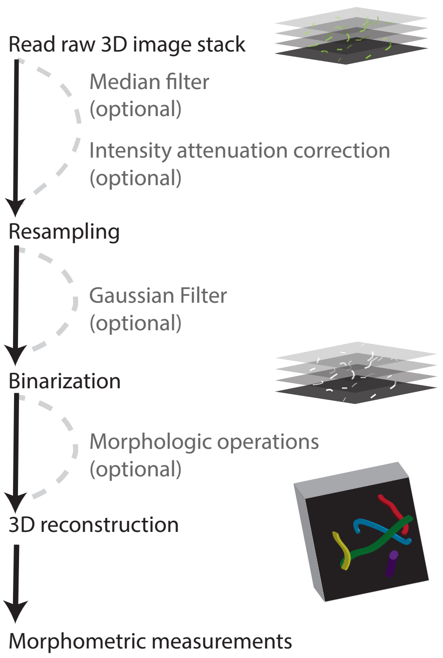

Figure 1. `Qiber3D`'s pipeline combines the required image processing steps for 3D morphometric quantification of networks. Optional tools are provided to cover a range of image content.

Implementation

Data IO

As interoperability is an essential goal of the `Qiber3D` toolkit, a wide variety of import and export options is paramount. Confocal images are usually acquired using commercial imaging platforms and the image files are saved in a proprietary file format containing the metadata. `Qiber3D`s support for multi-dimensional image formats is based on `PIMS`[2] (Python Image Sequence). This choice allows the use of essential image formats like `.tiff`-stacks as well as proprietary file formats from microscope vendors like Leica, Nikon, Olympus, and Zeiss as input. Physical size information (the voxel size) and, for multi-channel images, the channel of interest for network reconstruction is provided upon image loading or set as configuration variable for automated workflows. For some file formats, `Qiber3D` is able to extract the required metadata directly from the input file. Besides loading 3D image stacks to create the *Network* object, it can be built from files describing the network. `Qiber3D` supports the MicroVisu3D format `.mv3d`, traditionally used for vascular networks, as well as the `.swc` and the `.ntr` format, popular for neuronal networks.

The internal representation of the `Qiber3D` network can be stored as a binary file (`.qiber`) that allows for fast loading of the reconstructed network into the software. Easy visualization in web applications, and the import into specialized rendering software like `Blender` is achieved by saving the 3D representation as a collection of truncated cones in the `.x3D` file format. Moreover, `Qiber3D` supports several human-readable formats. The spatial data of the reconstructed network can be exported as `.mv3d`, `.swc` and `.csv` files. When exporting to a `.json` or Microsoft Excel `.xlsx` file format using *openpyxl*[3], the complete set of metadata and calculated properties is included. Furthermore, the network can be exported as a 3D `.tiff` image stack.

Image pre-processing

Median filter (optional). The primary purpose of the 3D median filter, also known as the despeckle filter, is the removal of speckles and extrema [20]. The median of its surrounding voxels replaces the value of each voxel. By default, a three voxels-wide neighborhood is used. However, this size can be modified in the configuration depending on the noise present in the image.

Intensity attenuation correction (optional). In 3D confocal images, light absorption can cause a decrease in signal intensity in slices located deeper into the sample. An exponential curve is fitted to the average intensities I_A in each of the slices to their physical stack position z to correct for this intensity attenuation (Fig. 2).

$$I_A = a \exp(bz) \tag{1}$$

The optimal parameters a and b for the intensity correction are determined using a non-linear least-squares fit.

Resampling to an isotropic voxel size. Commonly, the x/y resolution of image stacks differs from the resolution along the z-axis. As a cubic voxel size is beneficial to optimize the subsequent image processing steps, the z-axis or the x/y-plane of the image is resampled to a uniform resolution using a third-order spline interpolation [21].

Gaussian filter (optional). The image stack is blurred with a Gaussian filter simultaneously in all three dimensions to minimize the effect of noise on the image segmentation by reducing sharp differences between neighboring pixels. Applying a Gaussian filter reduces the noise level and imaging artifacts significantly. As the values now change smoothly from the outside to the inside of a structure, a border created by a cutoff will be more consistent and less rough.

Image segmentation

Binarization. The grayscale image is reduced to a binary representation to locate the structures' boundaries and label the segments.

1 https://github.com/marcomusy/vedo

2 https://github.com/soft-matter/pims

3 https://foss.heptapod.net/openpyxl/openpyxl

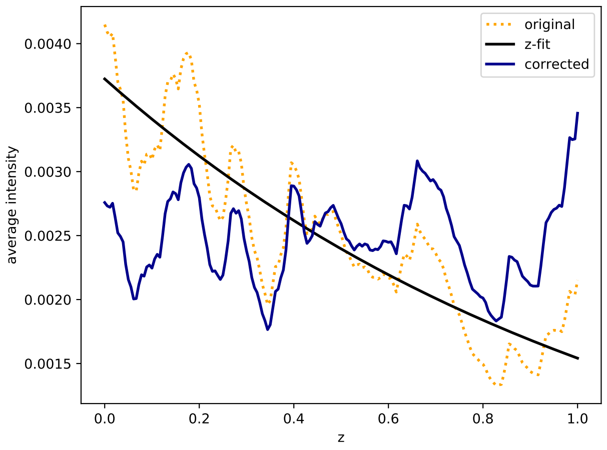

Figure 2. Intensity attenuation correction in the example image of the microvascular network. Yellow - original signal. Blue - corrected signal. Black - intensity fit.

All voxels equal to or greater than a threshold are set to True and all others to False. A dynamic threshold calculation for each stack is performed, permitting an automated workflow. By default, Otsu thresholding, an unsupervised, nonparametric method that tries to maximize the separability of the resultant classes (exactly two in the binary image), by utilizing the zeroth- and first-order moments of the histogram [22], is applied. Other thresholding algorithms can be selected, depending on the image. Alternatively, the threshold can be set directly as a percentage value of the signal intensity.

Morphological operations (optional). The obtained structures in the binarized image stack might not be perfectly solid, depending on the quality of the input data. A combination of dilation steps followed by an equal number of erosion steps fills small holes and compacts the segments' surface. The number of steps is configurable. In this section, small islands caused by imaging artifacts can also be removed based on a threshold set by the user.

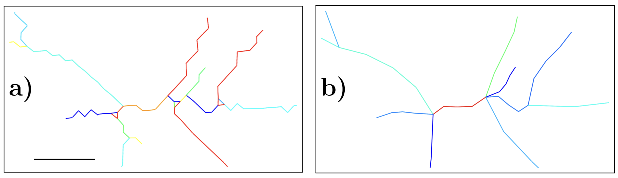

Figure 3. Network optimization. After thinning (a), the network is optimized by replacing tiny segments with more extensive structures and smoothing out voxel artifacts (b). Scale bar: 12.3 µm (10 voxel)

Network reconstruction

Reconstruction by thinning (default). The default network reconstruction approach is based on thinning, a morphological operation to remove selected foreground pixels from binary images. Initially, the image stack is distance transformed and every foreground (*True*) voxel in the stack is assigned the shortest Euclidean distance to a background (*False*) voxel. Subsequently, the Lee-Kashyap algorithm [23] is applied to extract the medial axis, and the binary image is reduced to its skeleton. The remaining foreground voxels, the skeleton, are modeled as a graph using *NetworkX* [24], defined by vertices that are connected by edges. Each foreground voxel represents a vertex, and connecting edges are formed between neighboring voxels. A radius is assigned to each vertex based on the earlier distance transformation. To form *Segments* (see below for details), the graph is reduced to contain only vertices representing end and branch points.

Distinctive edges are often formed along with branch points, sharp bends, or on the network's rim. Such edges occur between vertices that are direct neighbors and the resulting path is particularly jagged (Fig 3 a). This resolution artifact results in an overestimation of the fiber length and volume and an inflated branch point count. To mitigate these drawbacks, edges shorter than six voxels are merged with larger neighbors or removed if isolated and each edge is interpolated using a cubic spline (Fig. 3 b). New points are generated by default at a rate of approximately one point every ten voxels. All edges are fit to a spline with at least five points.

Reconstruction with TEASAR (alternative). Initially, the TEASAR method aimed to generate organ centerlines from 3D imaging generated by MRI, or CT scans [13, 14]. It has been used in a variety of applications, from pore networks in clay rocks [25, 26], to neuronal networks [27, 28] since. `Qiber3D` incorporates the kimimaro[4] implementation of the TEASAR algorithm that was developed to skeletonize neurons. For processing networks that resemble neuronal structures, that is, branching of structures (dendrites) from a cell body (soma), the use of this method is recommended over the thinning-based reconstruction. The output of the skeletonization step is a connected graph, from which we extract the quantitative measurements of the network.

Morphometric measurement

In `Qiber3D` the reconstructed network is represented in a hierarchical structure (Fig. 4). We use the terms *Network*, *Fiber*, and *Segments* to describe the components of the reconstruction. Note that these expressions are purely used conceptually to label `Qiber3D`'s output and that the terms might not refer to the actual structure. A *Fiber* might be a real fiber, an elongated cell, or another object depending on the application.

The largest entity is the *Network*, which represents the entirety of the structure. It is composed of a collection of *Fibers* formed by connected *Segments*, the smallest elements. A *Segment* is described by a collection of sorted points stored along the corresponding radius. The vertices between the points are interpreted as truncated cones. *Segments* end when they reach a branch point (grey points, Fig. 4). Therefore, *Segments* themselves are never branched. A branch point belongs to all *Segments* that it connects.

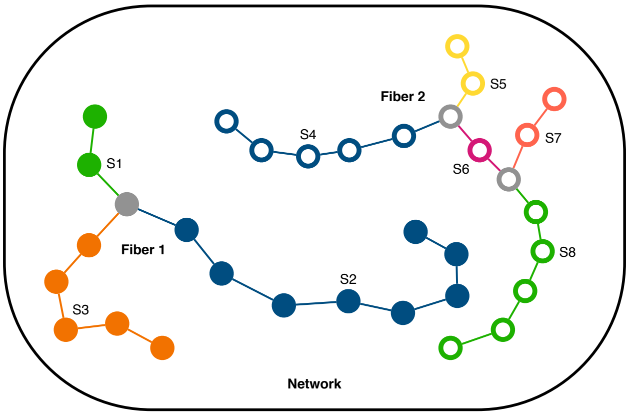

Figure 4. Qiber3D's hierarchical structure. *Segments* S1-S3 generate *Fiber 1* (filled points) and segments S4-S8 *Fiber 2* (hollow points), forming the *Network*. Branch points are colored in gray.

Each element, on the different hierarchical levels, is defined by a unique identifier and several quantitative properties, for example,

[4] `https://github.com/seung-lab/kimimaro`

the volume or the average radius. The average radius can be misleading considering that the distance between the points forming an element can be non-uniform, resulting in a skewed measurement. Therefore, we included the notion of a length-weighted cylindrical radius and return the radius of a cylinder with the same volume and length as the element of interest. While modeling the volume as overlapping truncated cones is sufficient in most cases, an improved volume estimation can be obtained from the rasterized network. As the start and endpoint of a *Fiber* within a given 3D image stack is interchangeable, the directional data is analyzed based on the assumption that all *Fibers* are pointing upwards (positive z-axis). Depending on the application, *Fibers* can be convoluted and the orientation of the *Segments* can be more meaningful in some cases. In both cases, the orientation of each element is described using the azimuth and altitude regarding a half-sphere.

For the *Network* additional measurements, like the number of *Fibers*, *Segments* and branch points, or the bounding box volume, are provided. The *Network* object also stores the relevant metadata of the input.

Visualization

`Qiber3D` uses `vedo`, a lightweight python module, that is based on `VTK` [29] and `numpy` [30], to visualize the network in 3D. The embedded rendering capability allows the users to quickly inspect a network by rotating the camera view and zooming into regions of interest. A linked view of the different reconstruction steps and the resulting skeleton enables the user to examine them in relation to each other. The network's color can be customized to represent different network properties, such as fiber length, volume, or average radius. In addition to the interactive visualization, 3D views can be exported as static images or animations.

Results

To provide a comprehensive overview of the features, `Qiber3D` was applied to the synthetic example image as well as two experimental datasets, an *in vitro* microvascular network and a neuron that was reconstructed elsewhere.

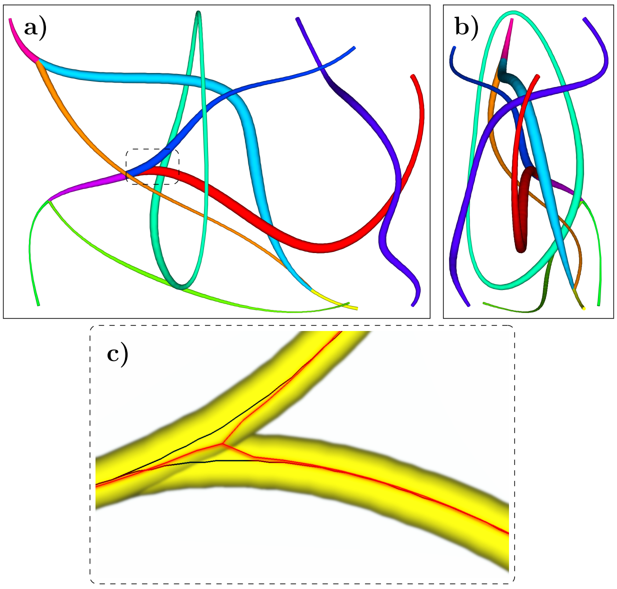

Figure 5. Synthetic network example with a) view on the x/y-plane and b) view on the z/y-plane. c) A branch point of the synthetic network with the original (black) and reconstructed (red) centerlines.

Synthetic example image

The output of the synthetic example image is presented in Fig. 5 and Suppl. Movie 1[5]. The synthetic example network was visualized in 3D and the segments composing the fibers were observed (Fig. 5 a). The measurements of the synthetic network reconstructed with `Qiber3D` were in agreement with the input data (Tab. 1). Interestingly, the branch points of the fibers were slightly displaced (Fig. 5 c) without affecting the measured total volume of the synthetic network (Tab. 1). This discrepancy is due to the thickness of the fibers concealing the original merging points during reconstruction.

Table 1. Comparison of the synthetic network with the output of Qiber3D after reconstruction.

	synthetic network	`Qiber3D` output
Number of fibers	4	4
Total length [µm]	1141.44	1120.84
Total Volume [μm^3]	4688.67	4665.62
Average radius [µm]	0.94	0.96
Cylinder radius [µm]	1.14	1.15

Microvascular network

`Qiber3D` was used to analyze a confocal image of a cellular network derived from microvascular cells grown *in vitro* (Fig. 6 a).

The analysis was performed, including all optional procedures of the workflow (Fig. 6). The application of the median filter resulted in a clearer image with fewer extrema (Fig. 6 b). Upon correction of the intensity attenuation, the signal distribution was found more even along the z-axis (compare Fig. 6 b and c, lower panels). The quantitative observation was confirmed by the distribution of the mean signal intensity slice along the z axis before (Fig. 2, blue line) and after (Fig. 2, orange line) the correction step. If the z-drop correction was switched off, the vessels in the lower part of the image were lost after reconstruction of the microvascular network (Suppl. Fig. 1 b, d-f). Following the intensity attenuation correction, a Gaussian filter resulted in noise reduction and smoothing of the boundaries (Fig. 6 d). After pre-processing the image using the optional filters, image segmentation was performed. Morphological operations, in the form of a combination of dilation and erosion (each with five iterations) and the removal of islands smaller than 100 μm^3, were applied to the binary image (Fig. 6 e). Omitting the morphological operations prior to reconstruction, resulted in the presence of numerous small particles that were not connected to the microvascular network ('islands') (Suppl. Fig. 1 c-e). Finally, the skeleton of the microvascular network was successfully reconstructed from the 3D image stack (Fig. 6 f, Suppl. Movie 2[6]). Each step was visualized interactively while processing the input image and compared together afterwards (Suppl. Movie 3[7]). Removing the optional filter steps for the image of the microvascular-like network led to artifacts in the reconstructed network (Suppl. Fig. 1 b, e-f).

The distribution of network attributes can be visualized in `Qiber3D` in the form of a histogram. In Fig. 7 a the distribution of the cylinder radius in the cellular network is presented as an example. The fiber radii followed a normal distribution between 1 and 10 µm with an average at 6.2 µm. To visualize the directional distribution in 3D, we introduced a spherical histogram. In Fig. 7 b every bin represents a part of a half-sphere. The start point for every network fiber was considered to be at the center of the half-sphere. The segments of each fiber were averaged into a single vector that captures the fiber's dominant direction. As the surface areas of the

5 Suppl. Movie 1 - Synthetic Network 10.6084/m9.figshare.13633802
6 Suppl. Movie 2 - Microvascular network 10.6084/m9.figshare.13633805
7 Suppl. Movie 3 - Compare extraction steps 10.6084/m9.figshare.13633799

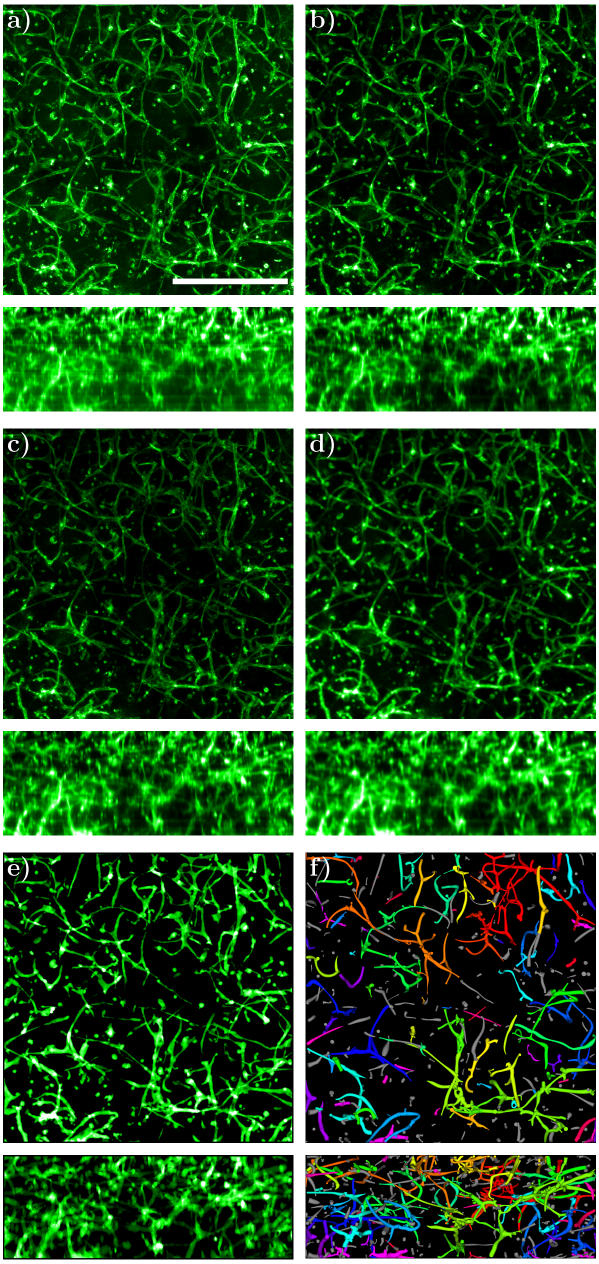

Figure 6. `Qiber3D`'s image processing workflow. An image of each step is shown as a average intensity projection along the z-axis (upper panels) and along the x-axis (lower panels). a) Raw image. Green: AlexaFluor 488-staining of CD31, a surface marker specific for endothelial cells. Scale bar: 500 µm. b) Image after median filter. c) Image corrected for intensity attenuation (z-drop correction). d) Image after Gaussian blur and surface compacting. e) Binarized image. f) Reconstructed microvascular network.

different bins of a half-sphere are not perfectly equal, the number of intersecting vectors was divided by the bin's surface area. Furthermore, the fiber density of each bin was scaled using the average fiber density over the half-sphere to allow for streamlined comparisons between multiple networks. The color scale indicates the scaled fiber density. For the microvascular network, the majority of fibers are located parallel to the x/y-axis (Fig. 7 b).

Processing a 1 GB nd2 file with `Qiber3D` on an Intel Core i7-6700 machine with 16 GB RAM running a Windows 10 (64-bit) operation system took approximately 7.5 minutes. Manual analyzing a similar image takes approximately 8.5 min, not considering the time to switch between various software packages [12]. While this is a slight decrease in processing time of one image, `Qiber3D` can be applied to numerous images without user interaction, making

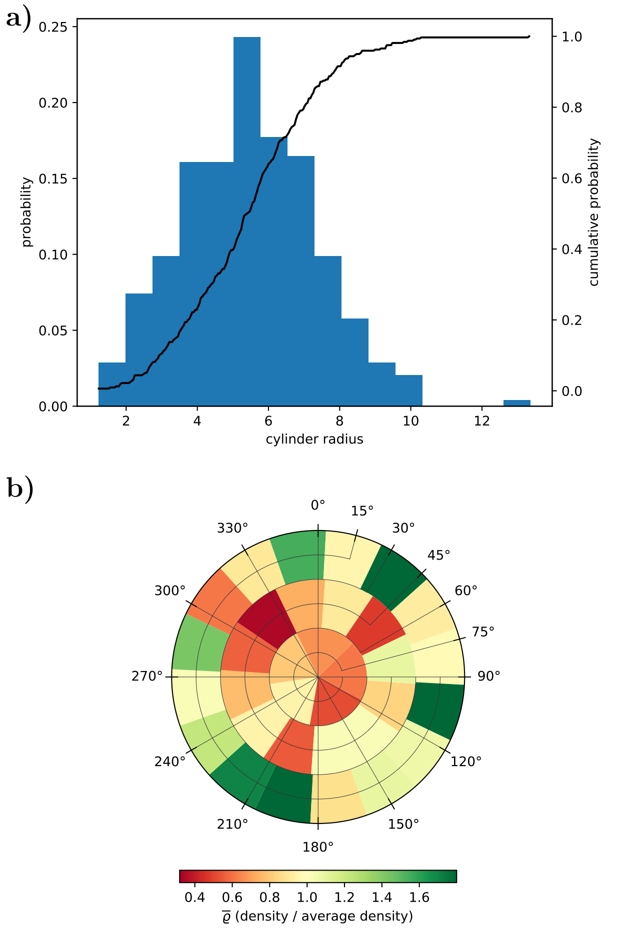

Figure 7. Graphical output of quantitative data from the microvascular network in Qiber3D. a) Distribution of the cylinder radius of the fibers within the network. b) Orientation distribution of the fibers in 3D.

it suitable to analyze large datasets. As `Qiber3D` is designed to run on a single CPU, running multiple processes of `Qiber3D` in parallel will accelerate the average image processing time for large datasets significantly. The use of built-in multiprocessing tools in Python enables straightforward implementation of parallel processing. For larger deployments on HPC clusters, task management using Message Passing Interface (MPI) for Python enables the analysis of vast image datasets. The implementation of `Qiber3D` as a Python package enables the smooth integration with other Python libraries to build customized tools that meet the requirements of varying computational environments, e.g., different HPC centers.

Neuron morphology

We used `Qiber3D` to visualize and measure a reconstructed neuron from a red-necked wallaby [31]. The published `.swc` file was obtained from NeuroMorph.org. We compared the 3D rendering of the neuron in `Qiber3D` with two other methods. The thickness of the structures was clearly visible in the `Qiber3D` visualization (Fig. 8 c, Suppl. Movie 4[8]) similar to the image on NeuroMorph.org (Fig. 8 b). In contrast, in the rendering with NLMorphology Viewer, a commonly used software tool to visualize neuron morphology, all fibers were displayed with the same diameter (Fig. 8 b). The measurements from `Qiber3D` were in agreement with the published data on the NeuroMorph.org website as well as the output from NLMorphology Viewer (Tab. 2). The quantification of the total length in `Qiber3D` excludes the soma of the neuron resulting in a slightly

8 Suppl. Movie 4 - Neuronal network 10.6084/m9.figshare.13633823

Table 2. Comparison of the quantitative output from the NeuroMorph.org website, NLMorphology Viewer software, and Qiber3D.

	NeuroMorph	NLMorphology Viewer	`Qiber3D`
Branch points	30	30	30
Average Diameter [µm]	1.09	na	1.38
Total length [µm]	5097.48	5046.92	4991.83
Total Volume [μm^3]	6362.05	6347.60	6288.30

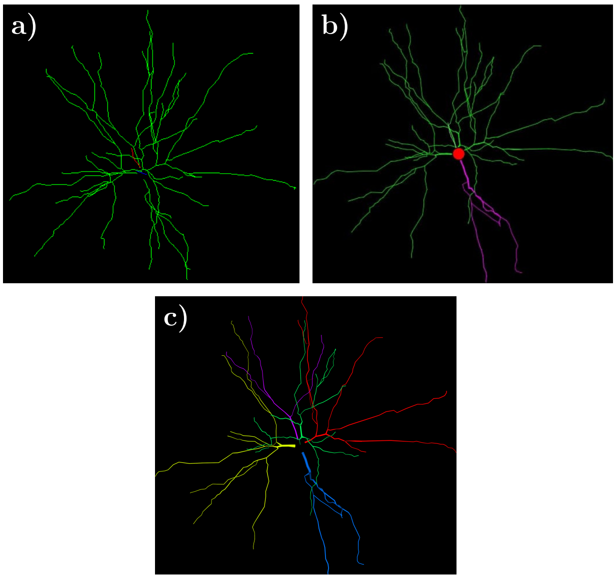

Figure 8. Visualization of the reconstructed neuron in a) NLMorphology Viewer, on b) NeuroMorph.org and c) with Qiber3D. Note, that the single neuron in this example represents exactly one fiber in Qiber3D.

lowered output compared to the measurements with the other tools.

Conclusion

Here we present `Qiber3D`, a toolkit to visualize, reconstruct and quantitatively analyze networks from 3D image stacks. `Qiber3D` combines the tools for a complete analytical workflow, from the raw image input to the morphometric quantification, within a highly configurable ecosystem. However, it can also be used in conjunction with other software packages, and integrated into existing analysis pipelines. By applying a building block principle, `Qiber3D` is developed to be highly customizable and adaptable for a variety of input datasets. By default, `Qiber3D` offers two skeletonization algorithms to cover a variety of input network types. The thinning-based core method of this software package is suitable for reconstructing cell-derived as well as artificial fibrous networks. Additionally, 3D reconstruction based on the kimimaro implementation of the TEASAR algorithm [13, 14] is possible in `Qiber3D`. The embedded visualization capability allows for the inspection of each image processing step to aid optimization of the image processing workflow. While the overall processing time is similar to manual processing, `Qiber3D` is designed to be used entirely hands-off to automate image analysis of large datasets. Running `Qiber3D`-based analysis on high-performance computing clusters makes it suitable for high-throughput processing. `Qiber3D`'s test-driven design within the Python ecosystem allows for long-term evolution of the project. For example, integration with TensorFlow and PyTorch will be of interest in the future to apply machine-learning algorithms for computer vision tasks. In summary, `Qiber3D` is a versatile 3D image analysis toolkit that is accessible for a wide range of research questions.

Methods

Cell culture

Prostate microvascular cells (PrMECs) were obtained from SciEnCell™ (Australian Biosearch, Wangara, WA, Australia) and expanded in endothelial cell medium (ECM) (Australian Biosearch, Wangara, WA, Australia). Cancer-associated fibroblasts (CAFs) were kindly provided by the Prostate Cancer Research Group, Department of Anatomy and Developmental Biology, Monash University [32]. The fibroblasts were cultured in RPMI 1640 media (no phenol red) (Gibco, ThermoFisherScientific, Scoresby, VIC, Australia) supplemented with 10 % fetal bovine serum (FBS) (Gibco, ThermoFisherScientific, Scoresby, VIC, Australia), 1 nM testosterone (Sigma-Aldrich, CastleHill, NSW, Australia), 10 ng mL^{-1} FGF-2 (MiltenyiBiotec, MacquariePark, NSW, Australia), 100 U penicillin, and 100 µg mL^{-1} streptomycin (Gibco, ThermoFisherScientific, Scoresby, VIC, Australia). All cells were maintained at 37 °C in a humidified incubator containing 5 % CO_2, with media changes every 2-3 days.

Preparation of hydrogel cultures

3D co-cultures were obtained using hydrogels comprised of synthetic starPEG and maleimide-functionalized heparin as described previously [33, 34]. Briefly, PrMECs and CAFs were seeded into hydrogels at a density of 6x10^6 and 6x10^5, respectively. Vascular endothelial growth factor (VEGF) (Peprotech, Lonza, MountWaverly, VIC, Australia), human fibroblast growth factor 2 (FGF-2), and stromal cell-derived factor 1 (SDF-1) (MiltenyiBiotec, MacquariePark, NSW, Australia) were included into the gel at a concentration of 5 µg mL^{-1} each. Additionally, 2 mol of RGD-SP (H2N-GCWGGRGDSP-CONH2) were added to the gel. A molar ration of starPEG to heparin-maleimide of 1:0.75 was used to obtain a stiffness of approximately 500 Pa (storage modulus). The starPEG-heparin hydrogels were maintained in ECM for 7 days at 37 °C in a humidified incubator containing 5 % CO^2.

Immunofluorescence of hydrogels

The cell-containing hydrogels were fixed in 4 % (v/v) paraformaldehyde (PFA) (Sigma-Aldrich, CastleHill, NSW, Australia) for 45 min. Blocking and permeabilization were achieved by incubation with 5 % goat serum (Gibco, ThermoFisherScientific, Scoresby, VIC, Australia) and 0.1 % Triton-X100 (MerckMillipore, Bayswater, VIC, Australia) in phosphate-buffered-saline (PBS) for 2 h at room temperature. Primary antibody staining against the endothelial marker CD31 (cat no. bba7, R&D Systems; 1:200 in 1 % goat serum) was performed overnight at 4 °C. Subsequently, the samples were washed in 1 % goat serum in PBS for 8 h with three changes of the washing buffer. Polyclonal goat anti-mouse IgG conjugated to Alexa-Fluor 488 (cat no. A11001, Invitrogen, ThermoFisherScientific, Scoresby, VIC, Australia; 1:300) secondary antibody, Alexa-Fluor 633 conjugated Phalloidin (Invitrogen, ThermoFisherScientific, Scoresby, VIC, Australia; 1:100), and 5 µg mL^{-1} 4',6-diamidino-2-phenylindole (DAPI) in 1 % goat serum/PBS were applied overnight at 4 °C. Images were acquired on a Nikon A1R inverted confocal microscope (Nikon Instruments Inc.; 10x, 1.32 µm px^{-1} x 1.32 µm px^{-1}, z-step size 2.5 µm x 181). Image analysis was performed on the

AlexaFlour-488 (green) channel of the acquired images to analyze the networks formed by the microvascular endothelial cells.

Availability of source code and requirements

- Project name: Qiber3D
- Project home page: https://github.com/theia-dev/Qiber3D
- Operating system(s): Platform independent
- Programming language: Python
- Other requirements: Python ≥ 3.7, for a list of required Python libraries, refer to the project's requirements.txt
- License: MIT
- biotoolsID: qiber3D
- RRID: SCR_021790

Availability of supporting data and materials

The raw images of the microvascular-like network is available as `nd2` and `tif` files at doi:10.6084/m9.figshare.13655606. Snapshots of our code and other supporting data are openly available in the GigaScience repository, GigaDB [35].

Declarations

List of abbreviations

CAF Cancer-associated fibroblasts
CT computed tomography
DAPI 6-diamidino-2-phenylindole
ECM endothelial cell medium
FBS fetal bovine serum
FGF-2 human fibroblast growth factor 2
GUI graphical user interface
HPC high-performance computing
MPI Message Passing Interface
MRI Magnetic resonance imaging
PBS Phosphate-buffered saline
PFA paraformaldehyde
PSF point-spread function
SDF-1 stromal cell-derived factor 1
TEASAR Tree-structure Extraction Algorithm for Accurate and Robust skeletons
VEGF Vascular endothelial growth factor

Ethical Approval

All experiments involving human cells were approved by the Queensland University of Technology Human Research Ethics Committee (Approval number: 1800000502).

Consent for publication

not applicable

Competing Interests

The authors declare that they have no competing interests.

Funding

AJ was supported by a Postgraduate Research Award (International), QUT. LB was supported by a grant from the National Breast Cancer Foundation (PF-16-004) and acknowledges the support of grant 1159637 awarded through the 2018 Priority-driven Collaborative Cancer Research Scheme and co-funded by Cancer Australia and Leukemia Foundation of Australia. Some of the data reported in this work were obtained at the Central Analytical Research Facility (CARF) operated by the Institute for Future Environments, QUT. Access to CARF was supported by the Science and Engineering Faculty, QUT.

Author's Contributions

AJ performed the experiments. HE and AJ developed the toolkit. AJ and HE analyzed and interpreted the data, and wrote the manuscript. AJ, HE and LJB read, edited and approved the final manuscript.

Acknowledgements

HE acknowledges the Duke University, Center for Autonomous Materials Design, for computational support and Prof. Stefano Curtarolo for fruitful discussions.

References

1. Carmeliet P, Jain RK. Angiogenesis in Cancer and Other Diseases. Nature 2000;407(6801):249–257. https://doi.org/10.1038/35025220.
2. Staton CA, Reed MWR, Brown NJ. A Critical Analysis of Current in Vitro and in Vivo Angiogenesis Assays. International Journal of Experimental Pathology 2009;90(3):195–221. https://doi.org/10.1111/j.1365-2613.2008.00633.x.
3. Conchello JA, Lichtman JW. Optical Sectioning Microscopy. Nature Methods 2005;2(12):920–931. https://doi.org/10.1038/nmeth815.
4. Rytlewski JA, Geuss LR, Anyaeji CI, Lewis EW, Suggs LJ. Three-Dimensional Image Quantification as a New Morphometry Method for Tissue Engineering. Tissue Engineering Part C, Methods 2012;18(7):507. https://doi.org/10.1089/ten.tec.2011.0417.
5. Stalling D, Westerhoff M, Hege HC. Amira: A Highly Interactive System for Visual Data Analysis. In: Visualization Handbook Elsevier; 2005.p. 749–767. https://doi.org/10.1016/B978-012387582-2/50040-X.
6. Peng H, Ruan Z, Long F, Simpson JH, Myers EW. V3D Enables Real-Time 3D Visualization and Quantitative Analysis of Large-Scale Biological Image Data Sets. Nature Biotechnology 2010;28(4):348–353. https://doi.org/10.1038/nbt.1612.
7. Fedorov A, Beichel R, Kalpathy-Cramer J, Finet J, Fillion-Robin JC, Pujol S, et al. 3D Slicer as an Image Computing Platform for the Quantitative Imaging Network. Magnetic resonance imaging 2012;30(9):1323–1341. https://doi.org/10.1016/j.mri.2012.05.001.
8. Eliceiri KW, Berthold MR, Goldberg IG, Ibáñez L, Manjunath BS, Martone ME, et al. Biological Imaging Software Tools. Nature Methods 2012;9(7):697–710. https://doi.org/10.1038/nmeth.2084.
9. Schindelin J, Arganda-Carreras I, Frise E, Kaynig V, Longair M, Pietzsch T, et al. Fiji: An Open-Source Platform for Biological-Image Analysis. Nature Methods 2012;9(7):676–682. https://doi.org/10.1038/nmeth.2019.
10. Lee E, Takahashi H, Pauty J, Kobayashi M, Kato K, Kabara M, et al. A 3D in Vitro Pericyte-Supported Microvessel Model: Visualisation and Quantitative Characterisation of Multistep Angiogenesis. Journal of Materials Chemistry B 2018;6(7):1085–1094. https://doi.org/10.1039/C7TB03239K.
11. Nishiguchi A, Matsusaki M, Kano MR, Nishihara H, Okano D, Asano Y, et al. In Vitro 3D Blood/Lymph-Vascularized Human

Stromal Tissues for Preclinical Assays of Cancer Metastasis. Biomaterials 2018;179:144–155. https://doi.org/10.1016/j.biomaterials.2018.06.019.

12. Bonda U, Jaeschke A, Lighterness A, Baldwin J, Werner C, De-Juan-Pardo EM, et al. 3D Quantification of Vascular-Like Structures in z Stack Confocal Images. STAR Protocols 2020;p. 100180. https://doi.org/10.1016/j.xpro.2020.100180.
13. Sato M, Bitter I, Bender MA, Kaufman AE, Nakajima M. TEASAR: Tree-Structure Extraction Algorithm for Accurate and Robust Skeletons. In: Proceedings the Eighth Pacific Conference on Computer Graphics and Applications IEEE Comput. Soc; 2000. p. 281–449. https://doi.org/10.1109/pccga.2000.883951.
14. Bitter I, Kaufman AE, Sato M. Penalized-distance volumetric skeleton algorithm. IEEE Transactions on Visualization and Computer Graphics 2001;7(3):195–206. https://doi.org/10.1109/2945.942688.
15. Virtanen P, Gommers R, Oliphant TE, Haberland M, Reddy T, Cournapeau D, et al. SciPy 1.0: Fundamental Algorithms for Scientific Computing in Python. Nature Methods 2020;17:261–272. https://doi.org/10.1038/s41592-019-0686-2.
16. van der Walt S, Schönberger JL, Nunez-Iglesias J, Boulogne F, Warner JD, Yager N, et al. scikit-image: image processing in Python. PeerJ 2014;2:e453. http://dx.doi.org/10.7717/peerj.453.
17. Kluyver T, Ragan-Kelley B, Pérez F, Granger B, Bussonnier M, Frederic J, et al. Jupyter Notebooks - a publishing format for reproducible computational workflows. In: Positioning and Power in Academic Publishing: Players, Agents and Agendas IOS Press; 2016.p. 87–90. http://dx.doi.org/10.3233/978-1-61499-649-1-87.
18. Abadi M, Barham P, Chen J, Chen Z, Davis A, Dean J, et al. TensorFlow: A System for Large-Scale Machine Learning. In: Proceedings of the 12th USENIX Conference on Operating Systems Design and Implementation USENIX Association; 2016. p. 265–283.
19. Paszke A, Gross S, Massa F, Lerer A, Bradbury J, Chanan G, et al. PyTorch: An Imperative Style, High-Performance Deep Learning Library. In: Wallach H, Larochelle H, Beygelzimer A, d'Alché-Buc F, Fox E, Garnett R, editors. Advances in Neural Information Processing Systems 32 Curran Associates, Inc.; 2019.p. 8024–8035.
20. Loizou CP, Pattichis CS. Despeckle Filtering Algorithms and Software for Ultrasound Imaging. In: Synth. Lect. Algorithms Softw. Eng., vol. 1; 2008.p. 1–166. http://dx.doi.org/10.2200/s00116ed1v01y200805ase001.
21. Thévenaz P, Blu T, Unser M. Image Interpolation and Resampling. In: Handbook of Medical Imaging Elsevier; 2000.p. 393–420. http://dx.doi.org/10.1016/b978-012077790-7/50030-8.
22. Otsu N. A Threshold Selection Method from Gray-Level Histograms. IEEE Transactions on Systems, Man, and Cybernetics 1979;9(1):62–66. https://doi.org/10.1109/tsmc.1979.4310076.
23. Lee TC, Kashyap RL, Chu CN. Building Skeleton Models via 3-D Medial Surface Axis Thinning Algorithms. CVGIP: Graphical Models and Image Processing 1994;56(6):462–478. http://dx.doi.org/10.1006/cgip.1994.1042.
24. Hagberg AA, Schult DA, Swart PJ. Exploring Network Structure, Dynamics, and Function using NetworkX. In: Proceedings of the 7th Python in Science Conference; 2008. p. 11 – 15.
25. Keller LM, Holzer L, Wepf R, Gasser P. 3D geometry and topology of pore pathways in Opalinus clay: Implications for mass transport. Applied Clay Science 2011;52(1-2):85–95. https://doi.org/10.1016/j.clay.2011.02.003.
26. Song Y, Davy CA, Troadec D, Blanchenet AM, Skoczylas F, Talandier J, et al. Multi-scale pore structure of COx claystone: Towards the prediction of fluid transport. Marine and Petroleum Geology 2015;65:63–82. https://doi.org/10.1016/j.marpetgeo.2015.04.004, cited By 46.
27. Evers JF, Schmitt S, Sibila M, Duch C. Progress in Functional Neuroanatomy: Precise Automatic Geometric Reconstruction of Neuronal Morphology From Confocal Image Stacks. Journal of Neurophysiology 2005;93(4):2331–2342. https://doi.org/10.1152/jn.00761.2004.
28. ya Takemura S, Bharioke A, Lu Z, Nern A, Vitaladevuni S, Rivlin PK, et al. A visual motion detection circuit suggested by Drosophila connectomics. Nature 2013;500(7461):175–181. https://doi.org/10.1038/nature12450.
29. Schroeder W, Martin K, Lorensen B, Kitware I. The Visualization Toolkit: An Object-oriented Approach to 3D Graphics. Kitware; 2006.
30. Harris CR, Millman KJ, van der Walt SJ, Gommers R, Virtanen P, Cournapeau D, et al. Array programming with NumPy. Nature 2020;585(7825):357–362. https://doi.org/10.1038/s41586-020-2649-2.
31. Jacobs B, Garcia ME, Shea-Shumsky NB, Tennison ME, Schall M, Saviano MS, et al. Comparative morphology of gigantopyramidal neurons in primary motor cortex across mammals. Journal of Comparative Neurology 2018;526(3):496–536. https://doi.org/10.1002/cne.24349.
32. Lawrence MG, Taylor RA, Toivanen R, Pedersen J, Norden S, Pook DW, et al. A Preclinical Xenograft Model of Prostate Cancer Using Human Tumors. Nature Protocols 2013;8(5):836–848. https://doi.org/10.1038/nprot.2013.043.
33. Tsurkan MV, Chwalek K, Prokoph S, Zieris A, Levental KR, Freudenberg U, et al. Defined Polymer-Peptide Conjugates to Form Cell-Instructive starPEG-Heparin Matrices In Situ. Advanced Materials 2013;25(18):2606–2610. https://doi.org/10.1002/adma.201300691.
34. Bray LJ, Binner M, Holzheu A, Friedrichs J, Freudenberg U, Hutmacher DW, et al. Multi-Parametric Hydrogels Support 3D in Vitro Bioengineered Microenvironment Models of Tumour Angiogenesis. Biomaterials 2015;53:609–620. https://doi.org/10.1016/j.biomaterials.2015.02.124.
35. Jaeschke A, Eckert H, Bray LJ, Supporting data for "Qiber3D - an open-source software package for the quantitative analysis of networks from 3D image stacks". GigaScience Database; 2021. http://dx.doi.org/10.5524/100955.

Figure 1

Click here to access/download;Fig

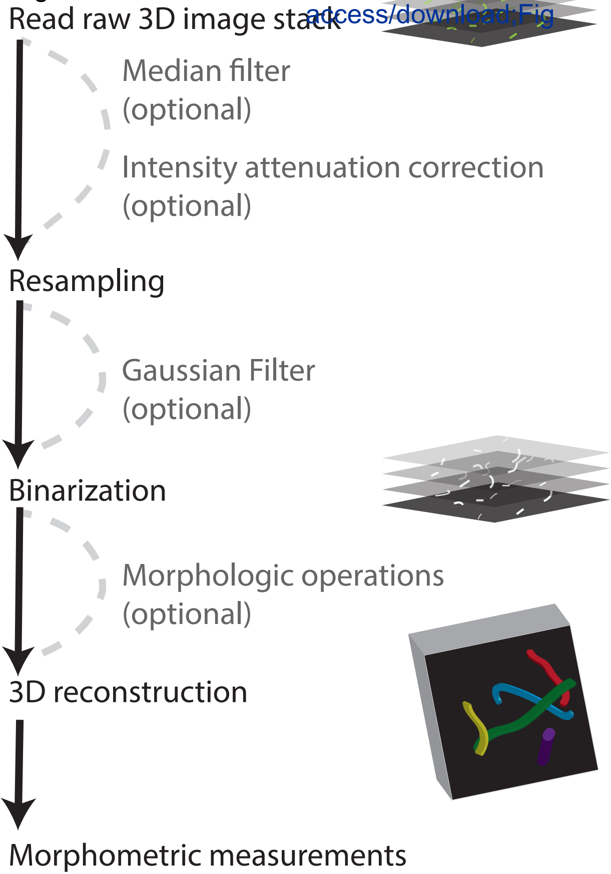

Figure 2

Click here to access/download;Figure;z-drop.pdf

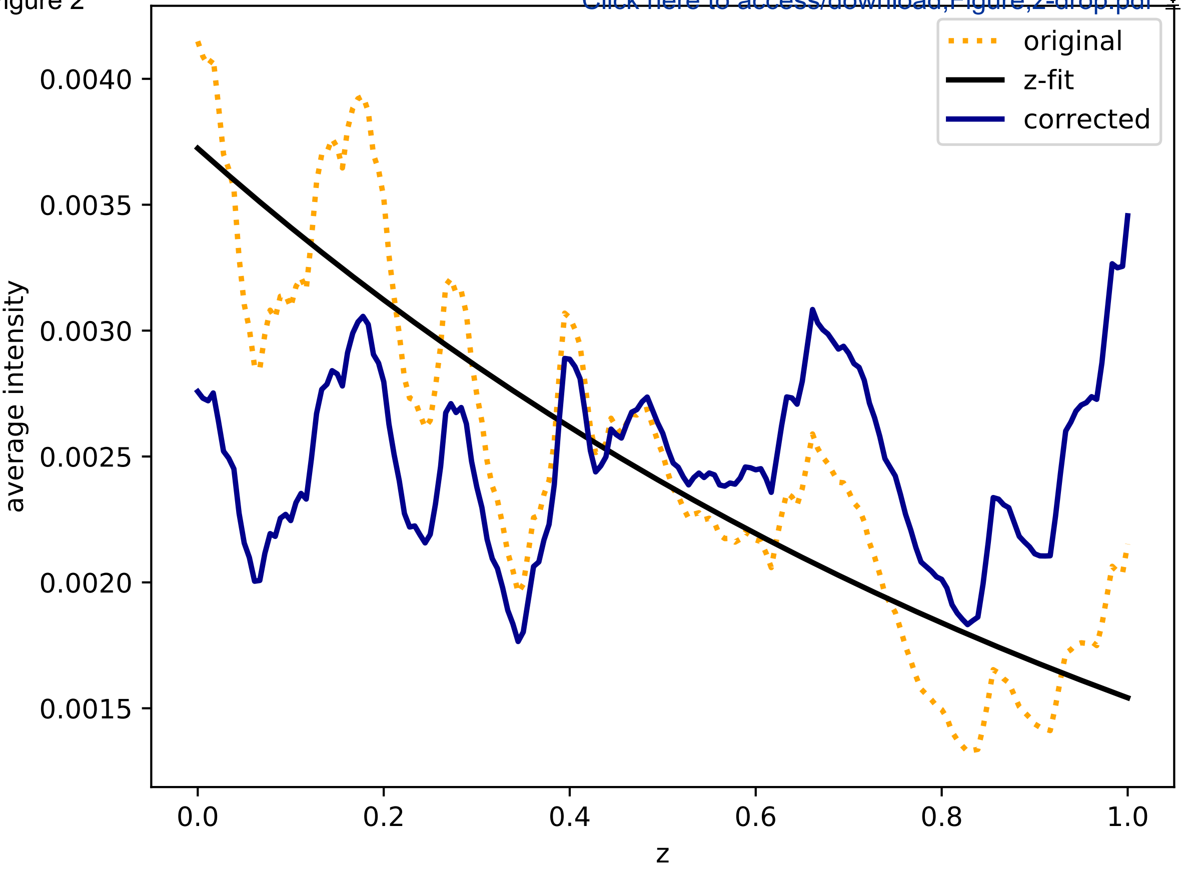

Figure 3

Click here to access/download;Figure;fig_recons

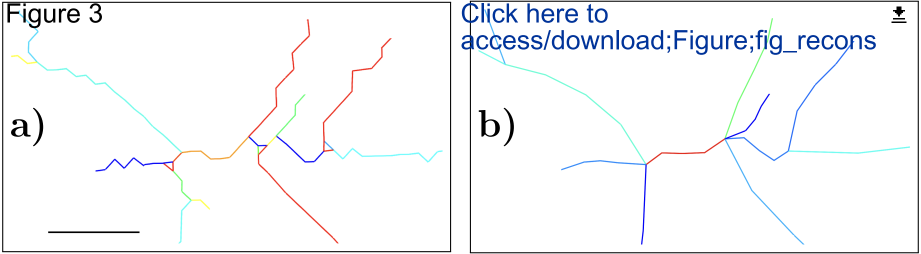

Figure 4

Click here to access/download;Figure;fig_network

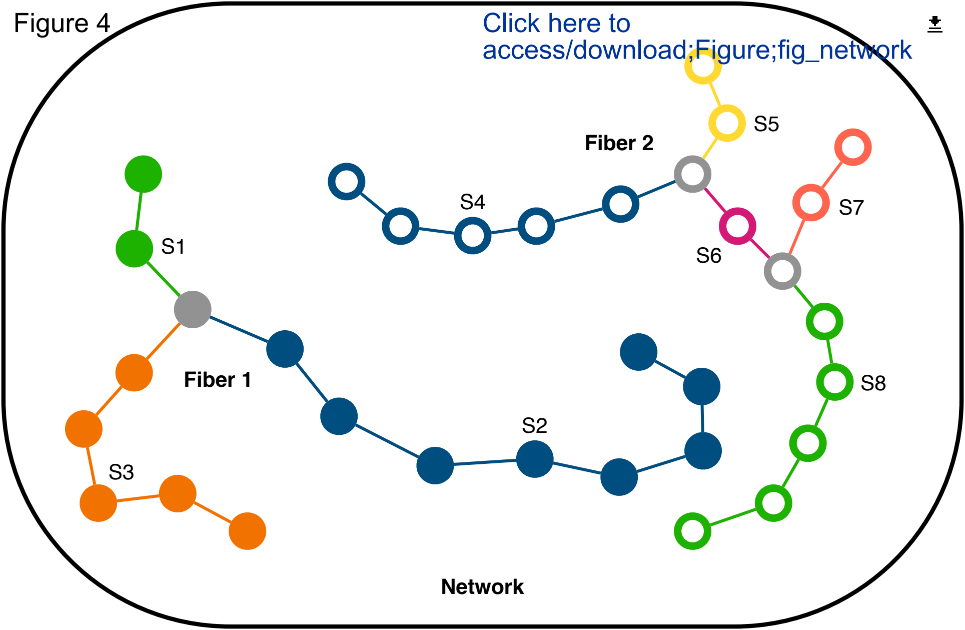

Figure 5

Click here to access/download;Figure;fig_syn_n

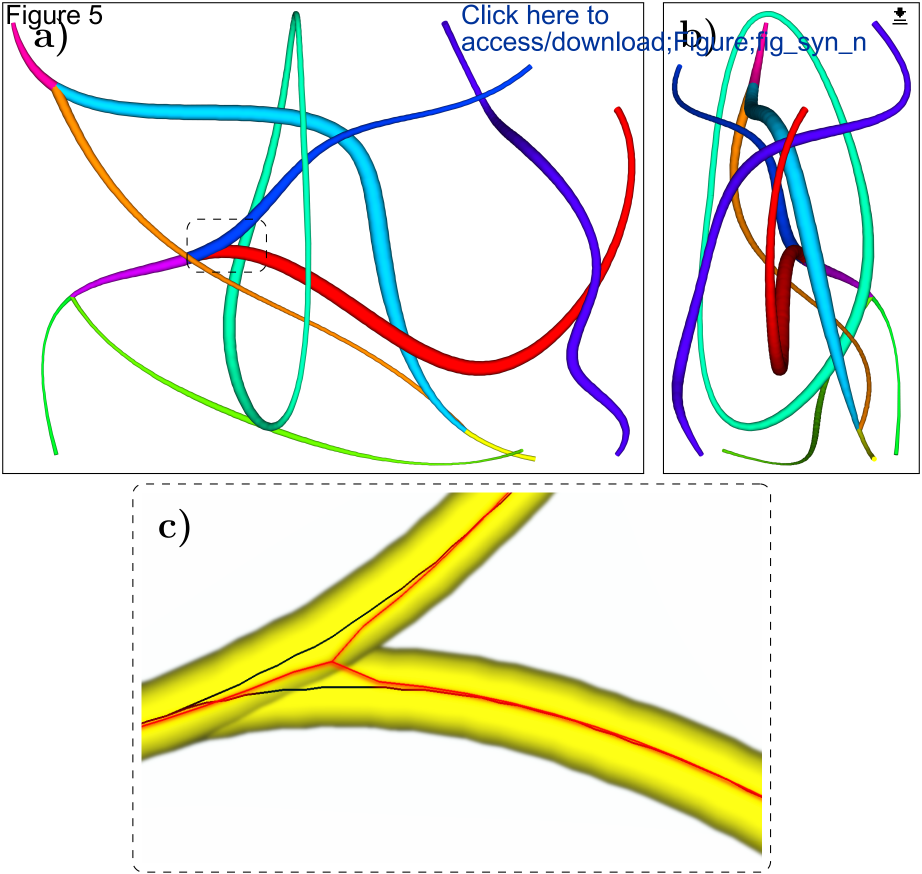

Figure 6

Click here to access/download;Figure;fig1.pdf

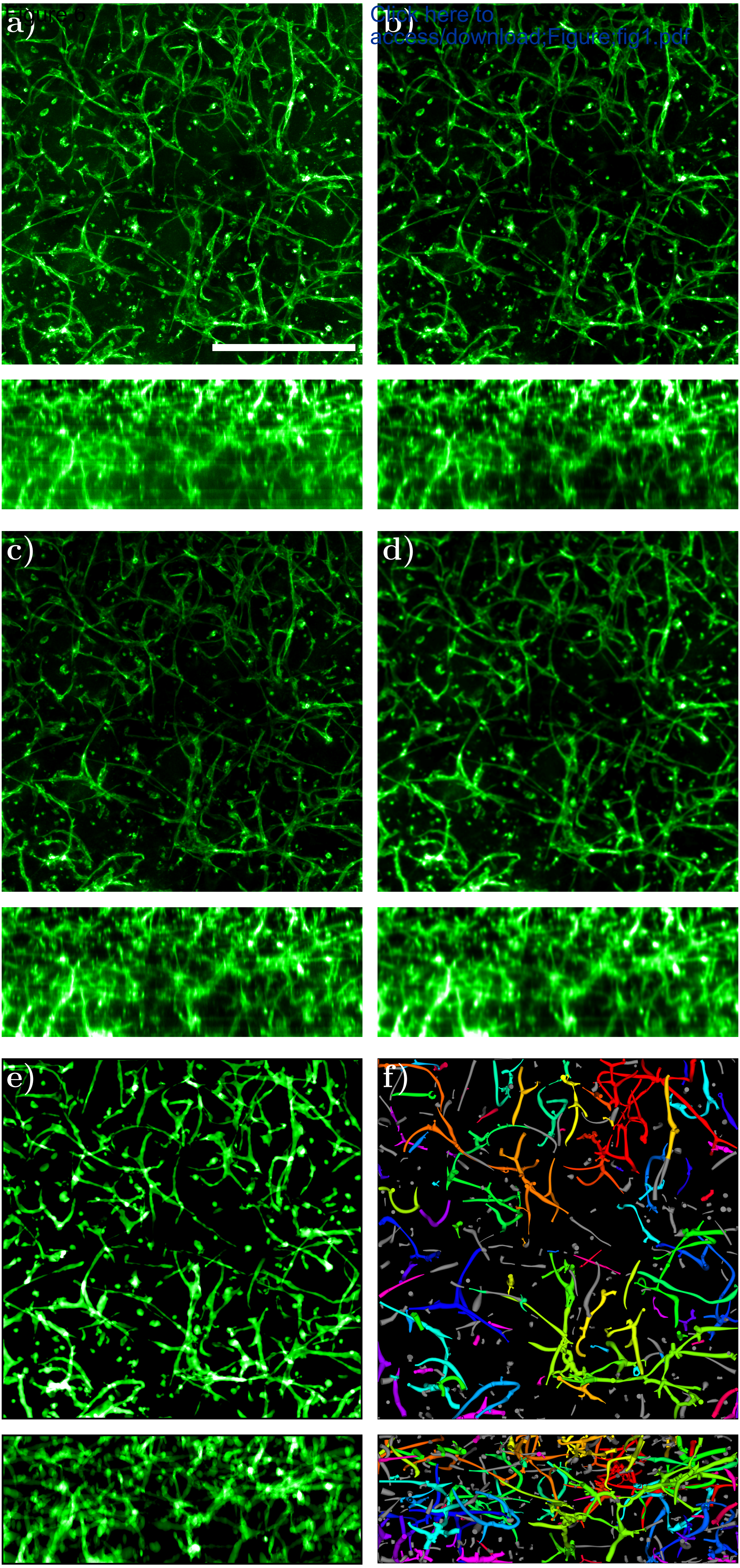

Figure 7

Click here to access/download;Figure;fig_ou

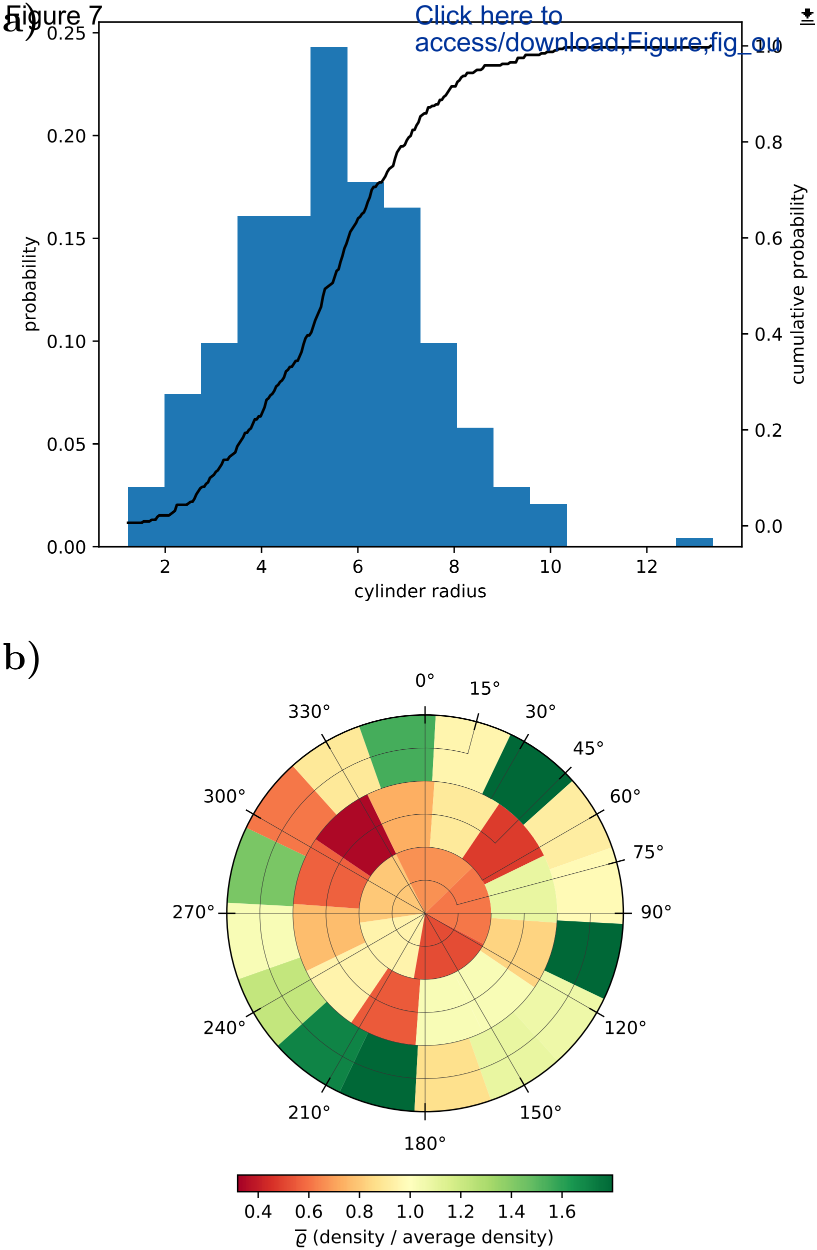

Click here to access/download;Figure;fig_ne

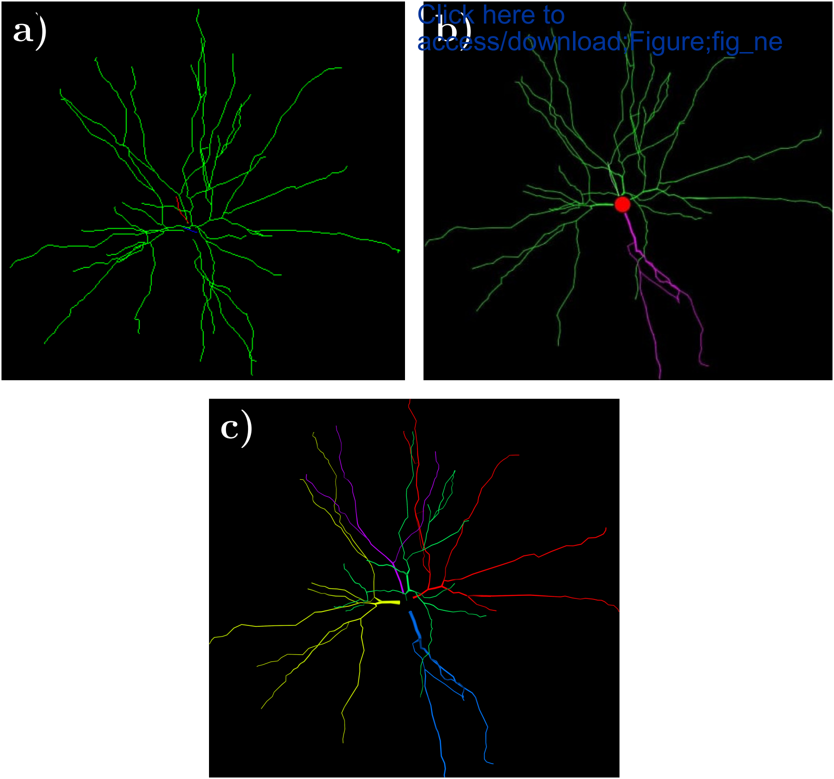

Figure S1

Click here to access/download;Figure;figs1.pdf

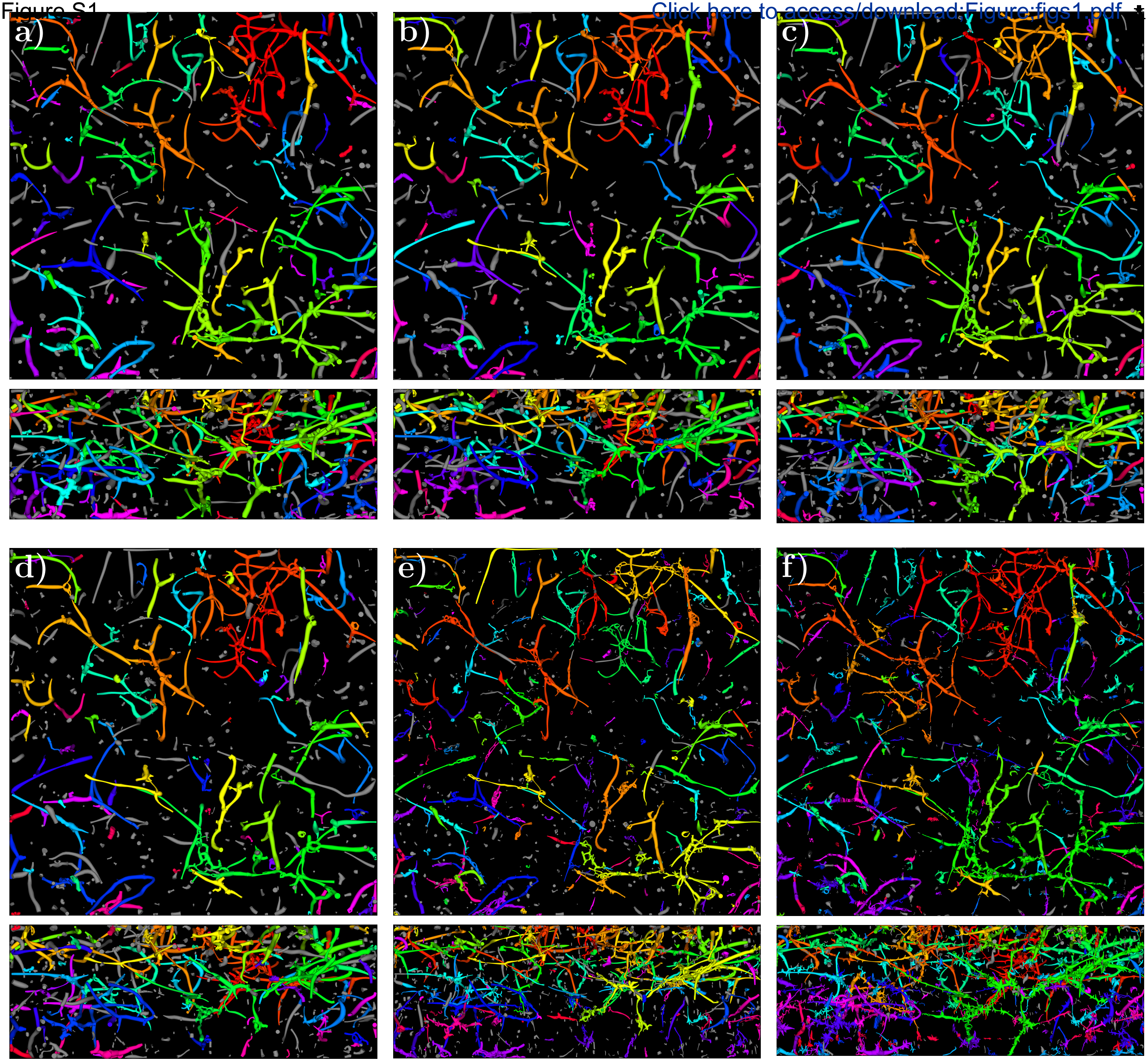

Supplementary Material

Click here to access/download
Supplementary Material
2020_Qiber3D_suppl.pdf
