# Supplementary material for: Qiber3D—an open-source software package for the quantitative analysis of networks from 3D image stacks
Source: Gigascience. 2022 Feb 4;11:giab091. doi: 10.1093/gigascience/giab091 (PMC8848317; doi:10.1093/gigascience/giab091)
Supplement: giab091_Supplemental_File [file giab091_supplemental_file.pdf]

GigaScience, 2017, 1–2

doi: xx.xxxx/xxxx
Manuscript in Preparation
Technical Note

TECHNICAL NOTE

Supplementary Material
Qiber3D - an open-source software package for the quantitative analysis of networks from 3D image stacks

Anna Jaeschke[1,2,3*,†], Hagen Eckert[4,†] and Laura J. Bray[1,2,5]

[1]Centre for Biomedical Technologies, Queensland University of Technology (QUT), Kelvin Grove, Australia and [2]School of Mechanical, Medical and Process Engineering, Science and Engineering Faculty, Queensland University of Technology (QUT), Brisbane, Australia and [3]Mechanobiology Institute, National University of Singapore, Singapore and [4]Department of Mechanical Engineering and Materials Science, Duke University, Durham, NC, USA and [5]ARC Training Centre for Cell and Tissue Engineering Technologies, Queensland University of Technology (QUT), Kelvin Grove, Australia

[*]anna.jaeschke@connect.qut.edu.au

[†]Contributed equally.

Key words: morphometric quantification; confocal imaging; image processing; vascular networks; fibrous networks; neurons

Supplementary Movies

Supplementary Movie 1 - Synthetic Network. 10.6084/m9.figshare.13633802
3D rendering of the synthetic example network coloured by segment, exported from `Qiber3D`.

Supplementary Movie 2 - Microvascular network. 10.6084/m9.figshare.13633805
3D rendering of the microvascular network colored by fiber, reconstructed with `Qiber3D`.

Supplementary Movie 3 - Compare extraction steps. 10.6084/m9.figshare.13633799
Screencast of the comparison view in `Qiber3D` that allows for side-by-side visualization of the raw image, the z-drop corrected image, the binary image and the reconstructed network.

Supplementary Movie 4 - Neuronal network. 10.6084/m9.figshare.13633823
3D rendering of the neuronal example network (red-necked wallaby, 10.1002/cne.24349) colored by segment, exported from `Qiber3D`.

Compiled on: December 3, 2021.
Draft manuscript prepared by the author.

Supplementary Figure

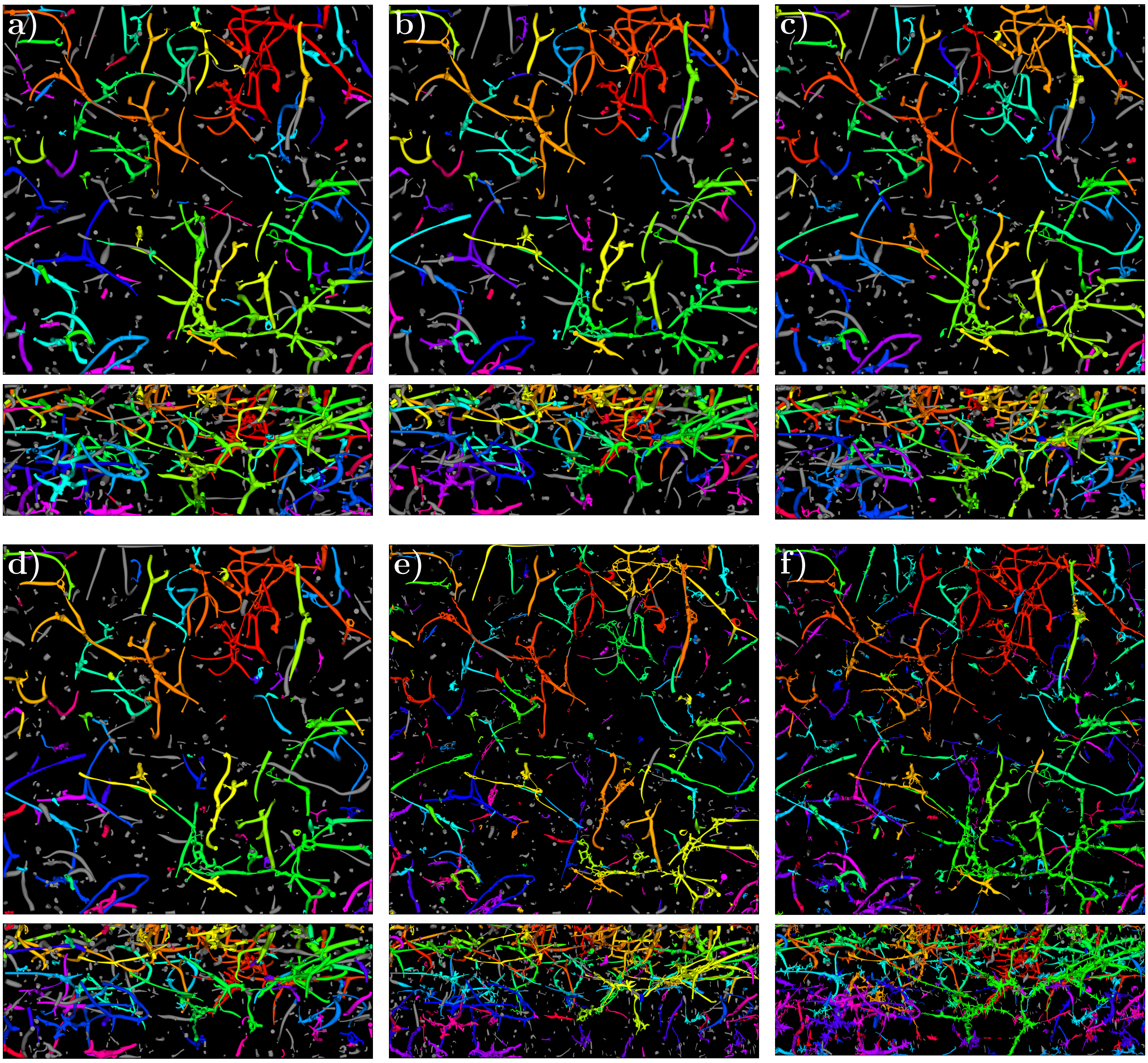

Supplementary Figure S1. `Qiber3D`'s image processing workflow with various combinations of optional steps in comparison to Fig. 6 in the main manuscript. An image of the final reconstructed network is shown as a average intensity projection along the z-axis (upper panels) and along the x-axis (lower panels). a) All optional steps turned on. b) Correction for z intensity attenuation omitted. c) Morphological operations following binarization omitted. d) Optional steps omitted except for Gaussian filter. e) Optional steps omitted except for median filter. f) Optional steps omitted except for morphological operations.
