# Supplementary material for: Qiber3D—an open-source software package for the quantitative analysis of networks from 3D image stacks
Source: Gigascience. 2022 Feb 4;11:giab091. doi: 10.1093/gigascience/giab091 (PMC8848317; doi:10.1093/gigascience/giab091)
Supplement: giab091_Response_to_Reviewer_Comments_Revision_1 [file giab091_response_to_reviewer_comments_revision_1.pdf]

Author's Response To Reviewer Comments

Close

The requested changes were made (reference included)

Close
