# Supplementary material for: Qiber3D—an open-source software package for the quantitative analysis of networks from 3D image stacks
Source: Gigascience. 2022 Feb 4;11:giab091. doi: 10.1093/gigascience/giab091 (PMC8848317; doi:10.1093/gigascience/giab091)
Supplement: giab091_Reviewer_1_Report_Original_Submission — Chris Armit -- 7/16/2021 Reviewed [file giab091_reviewer_1_report_original_submission.pdf]

Reviewer Report

Title: Qiber3D - an open-source software package for the quantitative analysis of networks from 3D image stacks

Version: Original Submission **Date:** 7/16/2021

Reviewer name: Chris Armit

Reviewer Comments to Author:

This interesting Technical Note describes the Qiber3D software tool, which is used for image analysis of 3D image stacks. There is a need to identify contiguous morphological components, such as alveolar airspaces and vascular networks, in 3D confocal image data and the Qiber3D toolkit was developed to address this issue. Importantly, the authors provide illustrative use case scenarios that include: 1) a synthetic example network; 2) a 3D reconstruction of the skeleton of a microvascular network from a confocal image stack of in vitro cultured mouse vascular cells, and; 3) a 3D reconstruction of a marsupial gigantopyramidal neuron. The software is publicly available from GitHub (https://github.com/theia-dev/Qiber3D) where it has been ascribed an Open Source Initiative-approved MIT license. In addition, test confocal image data of a 1GB vascular network used by Qiber3D is available on FigShare (https://doi.org/10.6084/m9.figshare.13655606.v1). These image data are of high quality and the Nikon ND2 files can be read using Fiji / ImageJ with the Bio-Formats plugin. Movies of the 3D reconstructions can be viewed using private sharing links.

Major comment

Can the authors please describe the morphological operations used by Qiber3D? Specifically, I wish to know which morphological operations (erosion, dilation, etc.) were used by Qiber3D to improve segmentation of the contiguous vascular networks in the image stack of in vitro microvasculature that is shown in Figure 6 and Supplementary Figure S1.

Minor comment

I request that the movies of the 3D reconstructions are made publicly available on FigShare. The DOIs for the four movies should be included in the revised GigaScience manuscript.

Level of Interest

Please indicate how interesting you found the manuscript: Choose an item.

Quality of Written English

Please indicate the quality of language in the manuscript: Choose an item.

Declaration of Competing Interests

Please complete a declaration of competing interests, considering the following questions:

- Have you in the past five years received reimbursements, fees, funding, or salary from an organisation that may in any way gain or lose financially from the publication of this manuscript, either now or in the future?
- Do you hold any stocks or shares in an organisation that may in any way gain or lose financially from the publication of this manuscript, either now or in the future?
- Do you hold or are you currently applying for any patents relating to the content of the manuscript?
- Have you received reimbursements, fees, funding, or salary from an organization that holds or has applied for patents relating to the content of the manuscript?
- Do you have any other financial competing interests?
- Do you have any non-financial competing interests in relation to this paper?

If you can answer no to all of the above, write 'I declare that I have no competing interests' below. If your reply is yes to any, please give details below.

I work for GigaScience where I perform checks on the quality of image data and the image analysis approach. My funding is not dependent on the outcome of this review, and I declare that I have no competing interests.

I agree to the open peer review policy of the journal. I understand that my name will be included on my report to the authors and, if the manuscript is accepted for publication, my named report including any attachments I upload will be posted on the website along with the authors' responses. I agree for my report to be made available under an Open Access Creative Commons CC-BY license (http://creativecommons.org/licenses/by/4.0/). I understand that any comments which I do not wish to be included in my named report can be included as confidential comments to the editors, which will not be published.

Choose an item.

To further support our reviewers, we have joined with Publons, where you can gain additional credit to further highlight your hard work (see: https://publons.com/journal/530/gigascience). On publication of this paper, your review will be automatically added to Publons, you can then choose whether or not to claim your Publons credit. I understand this statement.

Yes Choose an item.
