# Supplementary material for: Qiber3D—an open-source software package for the quantitative analysis of networks from 3D image stacks
Source: Gigascience. 2022 Feb 4;11:giab091. doi: 10.1093/gigascience/giab091 (PMC8848317; doi:10.1093/gigascience/giab091)
Supplement: giab091_Reviewer_2_Report_Original_Submission — Lucas Daniel Lo Vercio, Ph.D. -- 7/19/2021 Reviewed [file giab091_reviewer_2_report_original_submission.pdf]

Reviewer Report

Title: Qiber3D - an open-source software package for the quantitative analysis of networks from 3D image stacks

Version: Original Submission **Date:** 7/19/2021

Reviewer name: Lucas Daniel Lo Vercio, Ph.D.

Reviewer Comments to Author:

The authors are proposing an open software package for segmenting and quantifying networks in the biology domain. The article is well organized and they present two examples of usage.
Major concerns:
1. The authors state that the method works for different kind of biological networks. However, sometimes it is confusing in the text when they are describing the general problem and the particular problems. I recommend to carefully read and re-write for clarity. Maybe using a acronym when referring to general networks?
2. Figure 1 is comprehensive to understand the general workflow. However, as the article is presenting a software tool, it must present a diagram that shows the architecture of the library, and how the existing libraries cooperate. An UML-like diagram like class diagram or component diagram is suggested.
3. In the GitHub repository, I only found pre-defined networks as examples. I strongly suggest to add an example with a multi-page tiff (or other 3D format), due to that is the output of confocal images (among other flurorescene microscopy methods). With processed and/synthetic networks, it is not possible to assess the capacity of the library to deal with this data.
Minor comments:
1. Line 93: "We included a method to create synthetic network images" Which method? Is an existing method?
2. Line 113: The statement about the median filtering is correct. However, a reference must be included to support it. For example: Loizou and Pattichis. Despeckle Filtering Algorithms and Software for Ultrasound Imaging .2008
3. Line 125: Add a citation to support that method to have an isotropic volume.
4. Line 130: The gaussian filter homogenize neighbouring pixels. However, it diffuses the edges. The expression "more consistent boundaries" should be clarified.
5. Line 145: it says "erosion and dilation [...] to fill small holes". I think that first a dilation and then the erosion is the correct order to perform that filling.
6. Figure 6: The caption should clarify what image is being shown in the figure. I understand that are the microvascular cells.
7. Line 365: I am confused here. Are the Proste microvascular cells or the Cancer-associated fibroblasts used in this article? I only found that the first one are shown.
8. Line 522: Year is missing in the reference.

Level of Interest

Please indicate how interesting you found the manuscript: Choose an item.

Quality of Written English

Please indicate the quality of language in the manuscript: Choose an item.

Declaration of Competing Interests

Please complete a declaration of competing interests, considering the following questions:

- Have you in the past five years received reimbursements, fees, funding, or salary from an organisation that may in any way gain or lose financially from the publication of this manuscript, either now or in the future?
- Do you hold any stocks or shares in an organisation that may in any way gain or lose financially from the publication of this manuscript, either now or in the future?
- Do you hold or are you currently applying for any patents relating to the content of the manuscript?
- Have you received reimbursements, fees, funding, or salary from an organization that holds or has applied for patents relating to the content of the manuscript?
- Do you have any other financial competing interests?
- Do you have any non-financial competing interests in relation to this paper?

If you can answer no to all of the above, write 'I declare that I have no competing interests' below. If your reply is yes to any, please give details below.

I declare that I have no competing interests

I agree to the open peer review policy of the journal. I understand that my name will be included on my report to the authors and, if the manuscript is accepted for publication, my named report including any attachments I upload will be posted on the website along with the authors' responses. I agree for my report to be made available under an Open Access Creative Commons CC-BY license (http://creativecommons.org/licenses/by/4.0/). I understand that any comments which I do not wish to be included in my named report can be included as confidential comments to the editors, which will not be published.

Choose an item.

To further support our reviewers, we have joined with Publons, where you can gain additional credit to further highlight your hard work (see: https://publons.com/journal/530/gigascience). On publication of this paper, your review will be automatically added to Publons, you can then choose whether or not to claim your Publons credit. I understand this statement.

Yes Choose an item.
