# Supplementary material for: Qiber3D—an open-source software package for the quantitative analysis of networks from 3D image stacks
Source: Gigascience. 2022 Feb 4;11:giab091. doi: 10.1093/gigascience/giab091 (PMC8848317; doi:10.1093/gigascience/giab091)
Supplement: giab091_Reviewer_3_Report_Original_Submission — Alexandr Kalinin -- 7/26/2021 Reviewed [file giab091_reviewer_3_report_original_submission.pdf]

Reviewer Report

Title: Qiber3D - an open-source software package for the quantitative analysis of networks from 3D image stacks

Version: Original Submission **Date:** 7/26/2021

Reviewer name: Alexandr Kalinin

Reviewer Comments to Author:

The paper presents Qiber3D, a new Python toolkit for the quantitative analysis of fiber-like structures in 3D microscopic images. The library includes a number of image preprocessing operations, two different methods for the network reconstruction, hierarchical feature extraction, as well as input/output (IO) and visualization capabilities. Provided examples include applications of Qiber3D to both synthetic and real-world data, demonstrating the ability of the toolbox to extract meaningful representations of different types of networks (microvascular, neuron morphology) and extract features that are close to previously known measures.

While reviewing existing solutions for network quantification, the authors identified a few disadvantages: (1) most of these tools rely on 2D representations of 3D networks, which is not optimal; (2) most of the tools that support 3D data are often focused on the visualization aspect; (3) methods for processing and analysis steps are often available in different software packages, which makes building high-performance workflows for analyzing large datasets harder. The proposed solution is claimed to address these limitations.

The proposed solution is mostly implemented as a glue code for a number of Python libraries, including scikit-image for image processing, PIMS for IO, kimimaro for alternative skeletonization, NetworkX for building a graph representation of the network, etc. Therefore, the main contribution of this work is rather of an engineering type, as there are no contributions of novel methods or algorithms. However, in my opinion, the proposed toolbox does provide value for network analysis from 3D images in Python. The proposed solution enables straightforward construction of end-to-end pipelines from raw 3D image stack to tables of extracted features and visualizations.

While the paper is overall well-written and the methodological component of the approach is technically sound, the manuscript can be improved to strengthen the support for the claims made in the text. While reviewing relevant tools and literature, the authors talk about disadvantages of proprietary tools or 3D open source packages that focus on visualization and then cite Fiji. It should be clarified that Fiji does not primarily focus on 3D visualizationâ€”in fact, it supports (almost) all image processing operations provided in Qiber3D and supports skeletonization and skeleton analysis via plugins [3]. It seems like almost the whole Qiber3D can be implemented in Fiji and automated via macros for high-performance processing of large datasets. Similarly, CellProfiler also supports (almost) all of these operations, including analyzing skeletons [4] and running workflows in a parallel mode (Distributed CellProfiler). It would be helpful to stress some benefits of building a network quantification pipeline using Qiber3D instead of one of these tools. For example, the authors could discuss in more detail the issues with Java/Fiji and HPC systems, or the rapidly growing ecosystem of Python tools for image

analysis, an ability to run examples in the cloud with a Jupyter notebook, or better accessibility of GPU-enabled libraries if further speed up is needed. This will help to better identify the existing gap that Qiber3D is aiming to bridge and make its contributions to the community more clear.

Generally, when the main contribution of the paper is software, it is helpful if the authors identify some core design principles that they followed when developing this toolbox (e.g. flexibility vs. speed vs. ease-of-use, etc.). It helps the reader to follow the authors' thinkingâ€"how they dealt with the trade-offs they faced and design decisions they made during the process. For example, see discussions like this in [1] and [2].

The Findings (Implementation) section could benefit from better structure, because it reads more like a technical documentation when all operations are provided as a list. I suggest the authors group individual functions into topical subheading, e.g. "Image input", "Image pre-processing", "Segmentation", "Network reconstruction", etc. For example, it's confusing that the median filter and the Gaussian filter are separated by other operations.

For available operations that have some parameters fixed, it would be great to provide justification for the chosen parameter values. For example, why rescaling to isotropic voxels support only upscaling in Z, but not downscaling in XY (to the larger voxel size, which can be useful for processing speed up when dealing with large structures that are well resolved).

For filter operations, it'd be helpful to mention the choice of the filter shape (cube/ball) or the reasoning behind choosing parameters for merging jagged segments in the network optimization (e.g. do those depend on the size of the network?). Similarly for binarization, scikit-image supports at least 6 different methods for thresholding out-of-the-box (of which Li's and Triangle are often used for cell segmentation), so it is unclear why the authors only provide Otsu's method. I think making such decisions is fine, but the reasoning behind them should be made clear to the reader.

It is great that besides the thinning algorithm, the authors also provide TEASAR as an alternative. It would be great to see both of these algorithms applied to the same data to illustrate the difference between them. It is also not clear whether TEASAR was used for reconstruction in the Neuron morphology application.

Although confocal microscopy eliminates most of the out-of-focus light, images still exhibit background noise and spherical aberrations. Axial smearing directly affects morphological analysis of 3D structures, from their relative locations to their volumes. Assessment of these effects can be made by computing FFT of the image or modeling the PSF of the microscope. As the authors note in their own recently published STAR Protocols paper [5], image deconvolution is an important pre-processing step to combat these artifacts. Therefore, I am surprised to not find the deconvolution module in Qiber3D, especially since open source Python implementations of the common deconvolution algorithms are available, e.g. in scikit-image (which is already a dependency) or in FlowDec [6]. If the authors have their reasons to not include deconvolution in Qiber3D, this decision should be discussed in the main text.

Similarly, other popular methods for preprocessing include background removal and/or uneven illumination correction [7]. The authors may consider including them in the toolbox or mention why these are not often needed in network analysis pipelines.

It'd be great to have some example Jupyter notebooks that could be run in the cloud (with inline visualizations instead of opening a separate window).

Finally, I strongly encourage the authors to cite other open source tools that they used in Qiber3D [8].

They typically have the corresponding info on their GitHub page or in the documentation.

References:
[1] Paszke, Adam, et al. "Pytorch: An imperative style, high-performance deep learning library." Advances in neural information processing systems. 2019.
[2] Buslaev, Alexander, et al. "Albumentations: fast and flexible image augmentations." Information 11.2 (2020): 125.
[3] https://imagej.net/plugins/analyze-skeleton/
[4] https://cellprofiler-manual.s3.amazonaws.com/CellProfiler-4.0.4/modules/measurement.html?highlight=skeleton#measureimageskeleton
[5] Bonda, Ulrich, et al. "3D Quantification of Vascular-Like Structures in z Stack Confocal Images." STAR protocols 1.3 (2020): 100180.
[6] https://github.com/hammerlab/flowdec
[7] Singh, Shantanu, et al. "Pipeline for illumination correction of images for highâ€throughput microscopy." Journal of microscopy 256.3 (2014): 231-236.
[8] https://ilovesymposia.com/2019/05/02/why-you-should-cite-open-source-tools/

Level of Interest

Please indicate how interesting you found the manuscript: Choose an item.

Quality of Written English

Please indicate the quality of language in the manuscript: Choose an item.

Declaration of Competing Interests

Please complete a declaration of competing interests, considering the following questions:

- Have you in the past five years received reimbursements, fees, funding, or salary from an organisation that may in any way gain or lose financially from the publication of this manuscript, either now or in the future?
- Do you hold any stocks or shares in an organisation that may in any way gain or lose financially from the publication of this manuscript, either now or in the future?
- Do you hold or are you currently applying for any patents relating to the content of the manuscript?
- Have you received reimbursements, fees, funding, or salary from an organization that holds or has applied for patents relating to the content of the manuscript?
- Do you have any other financial competing interests?
- Do you have any non-financial competing interests in relation to this paper?

If you can answer no to all of the above, write 'I declare that I have no competing interests' below. If your reply is yes to any, please give details below.

I declare that I have no competing interests'

I agree to the open peer review policy of the journal. I understand that my name will be included on my report to the authors and, if the manuscript is accepted for publication, my named report including any attachments I upload will be posted on the website along with the authors' responses. I agree for my report to be made available under an Open Access Creative Commons CC-BY license (http://creativecommons.org/licenses/by/4.0/). I understand that any comments which I do not wish to be included in my named report can be included as confidential comments to the editors, which will not be published.

Choose an item.

To further support our reviewers, we have joined with Publons, where you can gain additional credit to further highlight your hard work (see: https://publons.com/journal/530/gigascience). On publication of this paper, your review will be automatically added to Publons, you can then choose whether or not to claim your Publons credit. I understand this statement.

Yes Choose an item.
